# Batch-effect detection, correction and characterisation in Illumina HumanMethylation450 and MethylationEPIC BeadChip array data

**DOI:** 10.1186/s13148-022-01277-9

**Published:** 2022-04-29

**Authors:** Jason P. Ross, Susan van Dijk, Melinda Phang, Michael R. Skilton, Peter L. Molloy, Yalchin Oytam

**Affiliations:** 1 Human Health Program, Health and Biosecurity, CSIRO, Sydney, Australia; 2 Charles Perkins Centre, The University of Sydney, Sydney, Australia; 3 Sydney Medical School, The University of Sydney, Sydney, Australia; 4 Sydney Institute for Women, Children and Their Families, Sydney Local Health District, Sydney, Australia; 5 Clinical Insights and Analytics Unit, South Eastern Sydney Local Health District, Sydney, Australia

**Keywords:** Methylation, Infinium, Batch-effect, Clustering, EWAS, False positives, SNP, ComBat, Harman

## Abstract

**Background:**

Genomic technologies can be subject to significant batch-effects which are known to reduce experimental power and to potentially create false positive results. The Illumina Infinium Methylation BeadChip is a popular technology choice for epigenome-wide association studies (EWAS), but presently, little is known about the nature of batch-effects on these designs. Given the subtlety of biological phenotypes in many EWAS, control for batch-effects should be a consideration.

**Results:**

Using the batch-effect removal approaches in the ComBat and Harman software, we examined two in-house datasets and compared results with three large publicly available datasets, (1214 HumanMethylation450 and 1094 MethylationEPIC BeadChips in total), and find that despite various forms of preprocessing, some batch-effects persist. This residual batch-effect is associated with the day of processing, the individual glass slide and the position of the array on the slide. Consistently across all datasets, 4649 probes required high amounts of correction. To understand the impact of this set to EWAS studies, we explored the literature and found three instances where persistently batch-effect prone probes have been reported in abstracts as key sites of differential methylation. As well as batch-effect susceptible probes, we also discover a set of probes which are erroneously corrected. We provide batch-effect workflows for Infinium Methylation data and provide reference matrices of batch-effect prone and erroneously corrected features across the five datasets spanning regionally diverse populations and three commonly collected biosamples (blood, buccal and saliva).

**Conclusions:**

Batch-effects are ever present, even in high-quality data, and a strategy to deal with them should be part of experimental design, particularly for EWAS. Batch-effect removal tools are useful to reduce technical variance in Infinium Methylation data, but they need to be applied with care and make use of post hoc diagnostic measures.

**Supplementary Information:**

The online version contains supplementary material available at 10.1186/s13148-022-01277-9.

## Background

Batch-effects can be defined as systematic differences between the measurements of different batches of experiments which artificially inflate within-group variances, thereby reducing experimental power and potentially creating false positive results. While high throughput genomic technologies are known to exhibit batch-effects, it is postulated they are also present in low throughput assays—it is the data resolution of high throughput technologies that offer the ability to identify and characterise them [1]. Batch-effects may arise from multiple factors, encompassing the laboratory environment, operating procedures, preanalytic sample variables and human factors such as fastidiousness and experience. Factors of particular concern to high throughput genomic technologies include: (1) sample quality, preservation and shipment, (2) the choice of methods or procedures, including nucleic acid isolation techniques and wash/clean-up conditions, (3) ambient conditions, including room temperature and ozone levels, (4) differences in scanner/other hardware and 5) lot-to-lot variance (discussed further in [2]).

In the instance of microarrays in particular, batch-effects often arise due to intra-batch differences in fluor labelling efficiency, dye bias (for two colour arrays), dye photobleaching, pipetting accuracy, buffer salt concentration, hybridisation temperature and time, array scanner variability and artefacts such as air bubbles in the hybridisation solution. As array scanning is not instantaneous, a time or position effect can also arise—with latter samples more subject to photodegradation or the damaging effects of ozone. It is known that Cy5 dye is more prone to photobleaching [3] and ozone degradation [4, 5] than Cy3 dye. Some features on the arrays are more prone to batch-effects. Known susceptibility factors include probe GC-content, DNA secondary structure, G stacking [6] and melting temperature [7]. For methylation arrays, the efficiency of DNA bisulphite conversion also needs to be considered.

At a global level, methylation estimates based on the Illumina Infinium technology are known to have high assay reproducibility [8]; however, a subset of probes have low correlation across technical and biological replicates [9, 10]. As with other genomic data, Infinium HumanMethylation450 (450K) and MethylationEPIC (EPIC) BeadChip arrays are subject to significant batch-effects. With 450K arrays, there are 12 discrete BeadChips on a glass slide and 8 discrete BeadChips for EPIC slides. Each glass slide is processed and scanned in parallel, so it is intuitive to define a batch structure as one slide. Best-practice laboratory methods seek to limit batch-effects by processing large sets of samples from multiple experimental groups together, with processing by experienced users employing multi-channel pipettes and/or automated liquid handlers. In a service facility, it is common to also process several of these slides across one day, thus creating a larger superset of batches by processing run (superbatch).

**Fig. 1:**
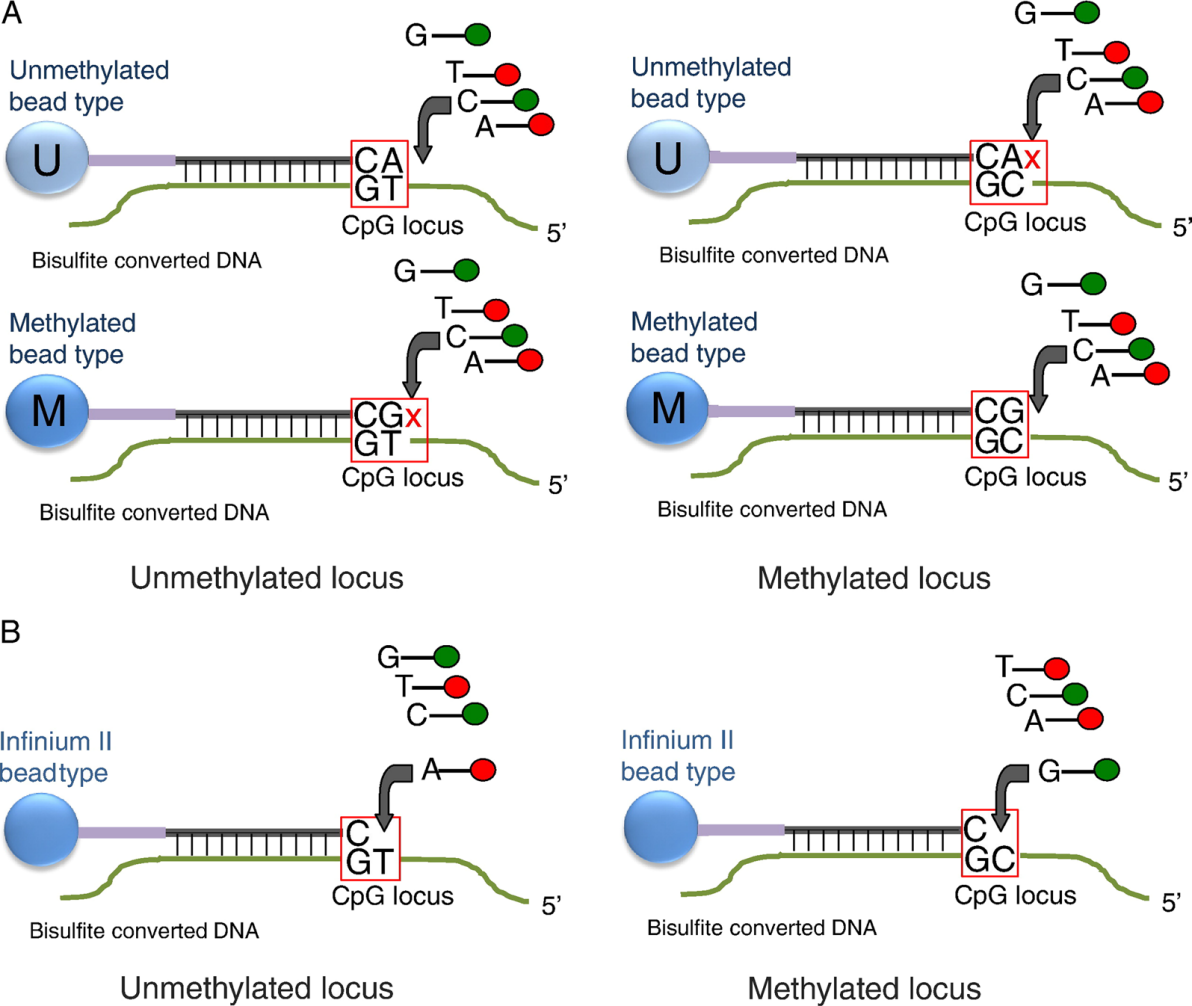
Infinium Methylation Assay scheme. **a** Infinium I assay. Two bead types correspond to each CpG locus: one bead type—to methylated (C), another bead type—to unmethylated (T) state of the CpG site. Probe design assumes same methylation status for adjacent CpG sites. Both bead types for the same CpG locus will incorporate the same type of labelled nucleotide, determined by the base preceding the interrogated ‘C’ in the CpG locus, and therefore will be detected in the same colour channel. **b** Infinium II assay. One bead type corresponds to each CpG locus. Probe can contain up to 3 underlying CpG sites, with degenerate R base corresponding to C in the CpG position. Methylation state is detected by single-base extension. Each locus will be detected in two colours. In the current version of the Infinium II methylation assay design, labelled ‘A’ is always incorporated at unmethylated query site (‘T’), and ‘G’ is incorporated at methylated query site (‘C’). Reproduced with permission from Bibikova et al. [8]

With Infinium methylation designs, probes designed for bisulphite-converted DNA bind to methylated and unmethylated alleles, which then allows for single-base extension with a labelled nucleotide across from the CpG site of interest. Subsequent staining of the extended template allows generation of a measurable fluorescence signal (Fig. 1). There are two probe types, Infinium I and Infinium II, which have different technical characteristics (Fig. 1). For Infinium I, the 3′ end of the probe is positioned directly across from the cytosine to be inspected and for Infinium II, immediately adjacent to the cytosine. Cyanine dye channel intensities are assigned as methylated and unmethylated signal depending upon the given probe design. For the Infinium I design probes, there are two probes in the same colour channel to quantify methylated and unmethylated alleles with the colour channel determined by the nucleotide immediately adjacent to the target cytosine (green fluorescing Cy3 for G/C and red fluorescing Cy5 for A/T). The Infinium II design uses only one probe to quantitate methylation with single-base extension from the 3′ end of the probe sequence resulting in either a red or green signal depending on whether the query site was unmethylated or methylated, respectively. So, unlike Infinium I probes, Infinium II probes confound red/green channel signals with methylation measurement. In addition, Infinium II probes also show a reduced dynamic range of measured methylation values as compared with Infinium I probes, presumably due to using a single bead for both alleles where the methylated and unmethylated signals become prone to residual emission by the other dye [11]. While Infinium II probes confound colour with methylation and have less signal dynamic range, they are far more common on the 450K and EPIC array designs due to their economy in measurement (one probe versus two probes).

It is also common for a probe sequence to span other CpG sites before the site under inspection. Infinium I probe pairs are designed assuming that all CpG sites spanning the probe are the same state as the measured CpG site: all methylated or all unmethylated. So, we could expect reduced signal at loci that are partially methylated. Infinium II designs use degenerate bases throughout the probe at CpG cytosine positions. This reduces bias against partial methylation at the expense of probe uniqueness and issues with cross-hybridisation. As such, Infinium II designs are used where there are fewer CpG sites traversed by a probe. This also confounds probe type with biology, as the Infinium I design will be used more often within CpG islands. Considering the known technical biases of 450K and EPIC data, it is important to undertake normalisation to account for this. Normalisation methods can either be within-sample or between-sample methods. It is also common to include background correction and dye-bias correction as part of this process.

Even with ideal experimental design and array normalisation, some residual technical noise will remain. It is common to use multi-variate techniques such as principal components analysis (PCA) to identify batch-effects and then to use specialised batch-effect removal software to adjust the data [1]. Given the expectation of batch-effects, it is important to design an experiment where the experimental factor to be considered is not completely confounded with the batch. If so, it becomes very difficult to separate biological variance (experimental factor) from technical variance (batch), as the latter must be identified, characterised and isolated to remove it. In an ideal situation, biological variance is orthogonal to technical variance which readily allows identification and separation.

There are caveats with batch-effect correction of methylation data. It is prudent to consider sources of sample-wise or feature-wise biological epigenetic variance which is potentially unrelated to the main experimental factor. If the source of variance has high population prevalence, it will often present by chance with unequal representation across each batch. If the biological variance is undeclared, batch-effect correction methods will naïvely mistake this as array feature technical variance and moderate batches with unequal representation towards the global mean. In DNA methylation studies, obvious sources of sample-wise biological variance are gender and cellular composition. In females, one of the X chromosome copies is inactivated [12] and there are also imprinted regions and other DNA gender-associated methylation differences on the autosomes [13]. When considering feature-wise biological variance on methylation arrays, a key consideration is genotype. This is often a result of a single nucleotide polymorphism (SNP) at the cytosine within the measured CpG site. C-to-T transversion events at CpG sites are the most frequent SNPs in the genome [14] and after bisulphite conversion, this inherited genotype is misrepresented as epigenetic state—an inherited ‘T’ allele and a bisulphite-treated unmethylated ‘C’ allele are identical. CpG sites might also have methylation rates influenced by a proximal or distal SNP. These cis-acting DNA polymorphisms can create allele-specific DNA methylation (ASM) [15]. In addition, nearby DNA repetitive elements may also be factors of influence and result in metastable epialleles, epigenetic modifications established stochastically during early development leading to variable expression in genetically identical individuals [16]. While sources of sample-wise biological variance can be declared a priori to a batch-effect correction algorithm, feature-wise biological variance is problematic. Filtering the features by lists of proximal SNPs does not account for unknown relationships between methylation state and distal SNPs, nor metastable epialleles or other abstruse mechanisms. To optimally apply batch-effect correction, we must empirically identify the features where the algorithm is distorting the data.

A further caveat is the choice of methylation metric. The quantification of methylation can be expressed as the log-transformed ratio of methylated over unmethylated signal (M) or the ratio of methylated over total (methylated plus unmethylated) signal (β) [17]. It is important to use M values when batch-effect correcting Infinium methylation data as these are unbounded. By definition, β is constrained between 0 and 1, and after correction there is no guarantee the values will still be within this range. M values can be batch-adjusted and then transformed back into the more readily interpretable β methylation values by a simple inverse logit transformation. With this transformation, very large negative and positive M values become asymptotic to β of 0 and 1, respectively.

To characterise batch-effects on Infinium Methylation BeadChip arrays, we took advantage of two of our in-house datasets studying the epigenetics of the developmental origins of health and disease (DOHaD) in neonates and employed two complementary correction methods; our batch-effect removal software, Harman [18], and the well-established ComBat package [19]. In the DOHaD space, variance due to biological phenotype can be subtle, which brings technical factors to the fore. To counter for this, cohort sizes are often relatively large. From the outset we planned for the identification and removal of batch-effects by employing a blocked experimental design. The two datasets examined were, (1) 450K arrays run on 369 neonate peripheral bloods, part of the EpiSCOPE (Epigenome Study Consortium for Obesity primed in the Perinatal Environment) study which makes use of samples from the DOMInO (Docosahexaenoic Acid (DHA) to Optimise Mother Infant Outcome) trial investigating the effect of in utero fish oil exposure [20] and (2), 169 EPIC arrays from neonate salivas as part of the Body Fatness and Cardiovascular Health in Newborn Infants (BFiN) study, a cohort investigating newborn body fatness and vascular health in the offspring [21, 22]. For the EpiSCOPE blood samples, gender and in utero fish oil exposure were orthogonal to batch (slide). In the BFiN saliva study, the samples were partitioned across slides to balance representation of gender and the body fatness distribution.

The scale of these experiments and the careful experimental design allowed a great opportunity to study the nature of batch-effects on Infinium Methylation arrays. Our blocked design allows orthogonality between biological variance and technical variance giving us the best opportunity to identify and eliminate batch-effects. Importantly, this lack of confounding allowed us to best identify which probes are most susceptible to batch-effects and to characterise the factors contributing to them. To understand the reproducibly of our findings, we considered a further three large public datasets and for each of the five datasets, we generated sets of erroneously corrected and batch-effect susceptible probes and created a reference matrix for all 450K and EPIC probes. This reference matrix will allow investigators to identify erroneously corrected and batch-effect susceptible CpG probes in their own EWAS projects.

## Results

### Experimental design and processing steps

For the EpiSCOPE study [20], DHA supplementation and gender were balanced as much as possible across the 12 450K BeadChips on each glass slide, with these factors also randomly distributed over the 6 rows and 2 columns of 31 slides (Additional file 1: Fig. S1). Blood DNA was extracted from Guthrie card blood spots and the 369 arrays were processed according to our blocking plan in six processing runs (superbatches) at the Australian Genome Research Facility (AGRF), Melbourne, Australia.

Similarly, the BFiN study [22] employed a blocked design over the 8 EPIC arrays on a glass slide. Apart from gender, the experimental factors of interest were continuous, so the samples were blocked over each slide to ensure (as much as possible) equal representation of gender and similar body fatness distribution across the 22 slides (Additional file 2: Fig. S2). As with the EpiSCOPE study, the position was randomised to avoid correlation with slide position. DNA was extracted from neonate saliva and the 169 arrays were processed in accord with our blocking plan across two processing runs (superbatches) at the Australian Cancer Research Foundation (ACRF) Cancer Genomics Facility, Adelaide, Australia. For both studies, further detail on the design and processing is given in the methods sections here and in the relevant study publication.

### Technical variation evident in control probes

The Infinium technology incorporates a number of control probe families to measure the efficiency and quality of the many steps in the protocol (the control classes are described by Illumina in their BeadArray Controls Reporter Software Guide document). Technical effects were evident across particular slides of both the EpiSCOPE and BFiN datasets. There was little commonality in the nature of these technical effects across the two datasets.

**Fig. 2:**
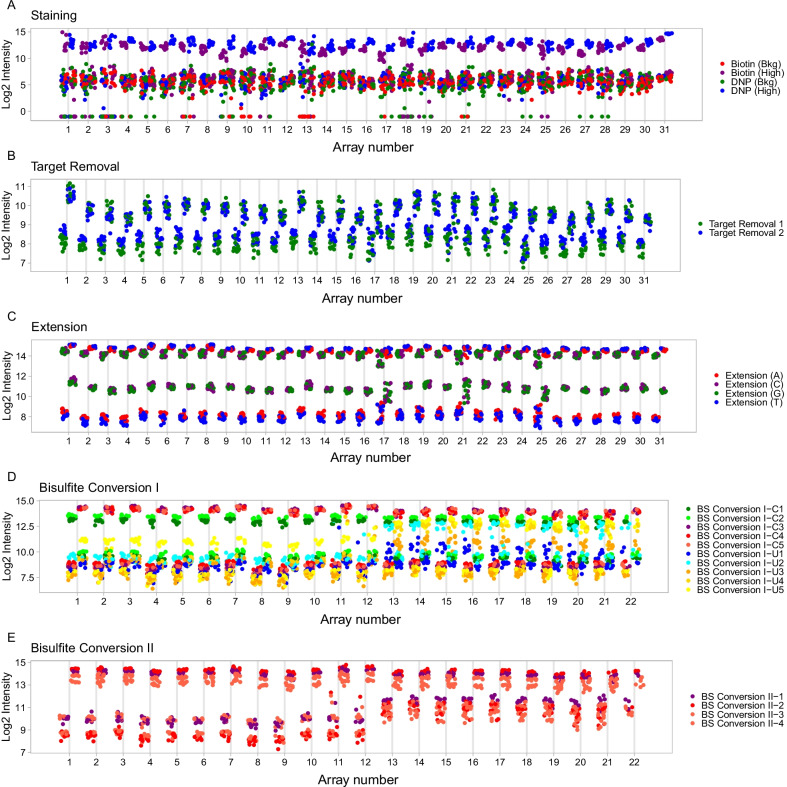
Selected Infinium control probes. Across the EpiSCOPE study data, outlier slides were observed for the staining (**a**), target removal (**b**), and **c** extension control probes. For the BFiN study data, the Type I (**d**) and Type II (**e**) bisulphite conversion control probes showed evidence of reduced bisulphite conversion efficiency for slides 13–22

Across the EpiSCOPE 450K data, we observed slides 9 and 17 had outlier probes for the staining controls with reduced fluorescence in the expected high intensity biotin beads (Fig. 2a). Slide 1 exhibited rather high intensity staining for the target removal probes (Fig. 2b). In the extension controls, some arrays on slides 17, 21 and 25 exhibited increased variances (Fig. 2c). Arrays from slides 17 and 25 were also outliers more broadly and exhibited less fluorescence for the hybridisation, extension, stripping, specificity and non-polymorphic controls, as well as the two bisulphite controls sets (Additional file 3: Fig. S3). Collectively, the EpiSCOPE study control probe analyses show some arrays on slides 17 and 25 have increased variability across a wide range of control metrics. This suggests slides 17 and 25 will also have increased technical variance for the experimental probes, which will be observed as a batch-effect.

Within the BFiN EPIC data, a pronounced difference between each superbatch was observed. Slides 13–22 (the entirety of superbatch 2) had large increases in intensity for the Type I unmethylated control probes (Fig. 2d) and Type II green channel probes (Fig. 2e). These control probe results suggest a reduced efficiency in the bisulphite conversion rate in superbatch 2. Some negative control probes on the red and green channels also had high fluorescence intensity for superbatch 2 (Additional file 4: Fig. S4). Otherwise, the control probes exhibited uniformity across slides. Collectively, this suggests the EPIC BeadChips were of high quality and that increased technical variance leading to batch-effect will more likely be across slides 13–22. With decreased efficiency of bisulphite conversion, probes with many CpG sites may mismatch across their length and there will be a bias in calling very low rates of methylation, as this requires high conversion efficiency.

### Colour balance

**Fig. 3:**
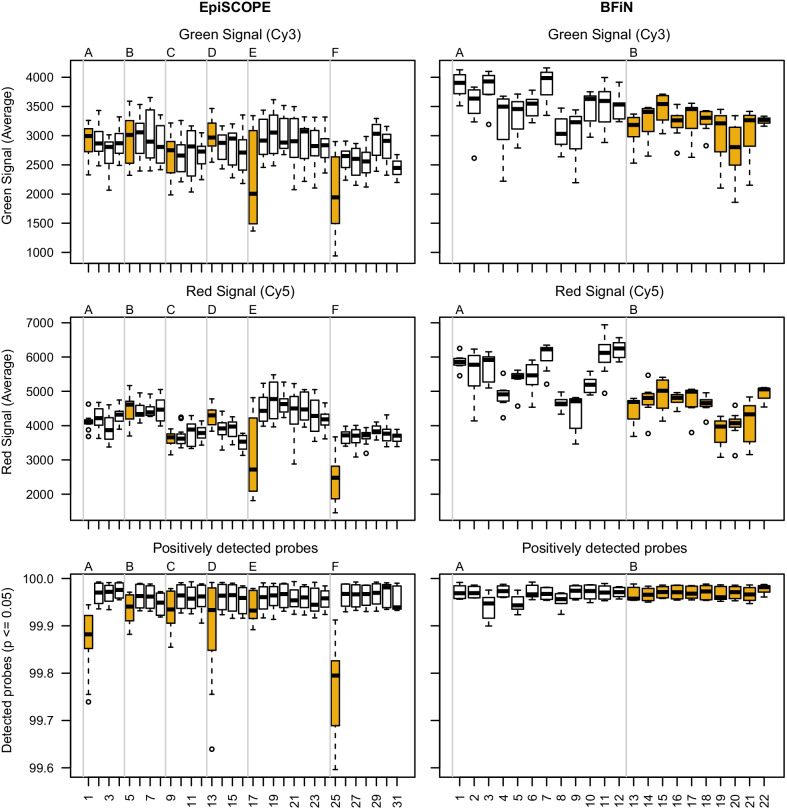
Correlation of processing run with signal intensity and positively detected probes. Green and red channel intensities and positively detected probes grouped by slide and processing order are presented for the EpiSCOPE and BFiN studies. The EpiSCOPE study was composed of 31 slides processed in six processing runs (superbatches A–F). The BFiN study was composed of 22 slides processed in two processing runs (superbatches A–B). Slides highlighted in gold illustrate the variation observed across superbatches. The first slide of the day for the EpiSCOPE study (slides 1, 5, 9, 13, 17, 25) and superbatch B for the BFiN study (slides 13–22)

Across the green (Cy3) and red (Cy5) signal intensities, we observed notable dye bias on both the 450K and EPIC array designs. For Cy3, there was consistently lower mean fluorescence intensity and less variability than Cy5 (Fig. 3).

When the same Cy3 and Cy5 mean fluorescence intensity data are considered by slide, a correlation with superbatch was also observed. We note the first EpiSCOPE slide in each of the six processing runs (superbatches A–F) typically had more or less Cy3 and Cy5 signal and a reduced fraction of positively detected probes (Fig. 3). Across the two BFiN processing runs (superbatches A–B), the second superbatch had less variability in Cy3 and Cy5 signal than the first. Slides 3 and 5 had a slightly reduced fraction of positively detected probes compared to the other slides.

**Fig. 4:**
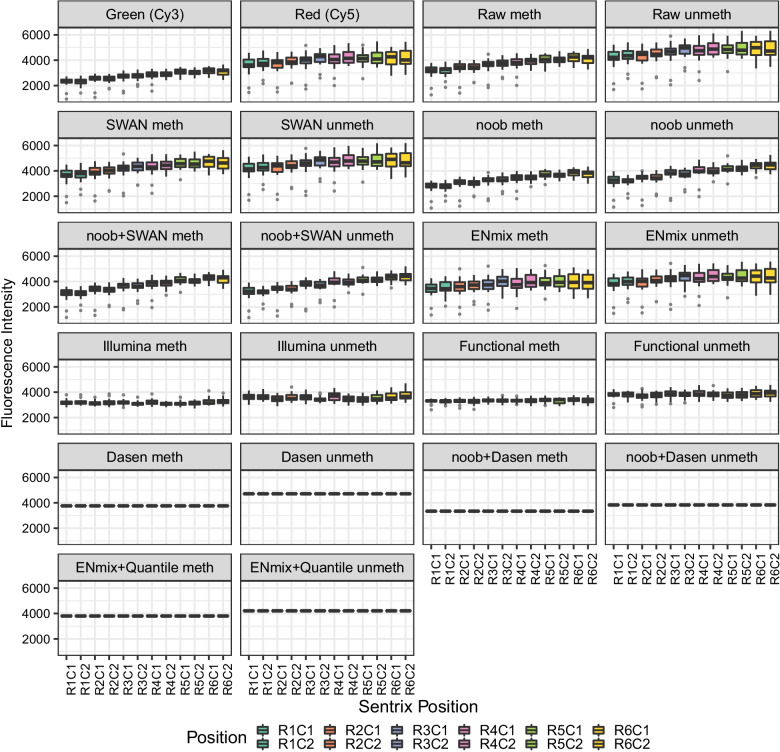
EpiSCOPE fluorescence intensity slide positional effect is reduced with preprocessing methods. Infinium green (Cy3 dye) and red (Cy5 dye) fluorescent intensities are formulated into methylated (meth) and unmethylated (unmeth) signals. These meth and unmeth signals are used to calculate β and M values. If the 369 BeadChips in the EpiSCOPE set are grouped by row (R) and column (C) position on the glass slide, there is evidence that the distribution of fluorescent intensities is associated with this position. The position effect diminishes with preprocessing methods. For some between-array methods no variation in mean is observed, as all the BeadChips have had mean fluorescent intensities moderated to be the same

A bias associated with BeadChip array position on the glass slide was also observed (Fig. 4 and Additional file 5: Fig. S5). There was a distinct trend of lower fluorescence intensity in the first 2 or 3 rows compared to the latter rows. The EPIC array had reduced Cy3 and Cy5 fluorescence (Additional file 5: Fig. S5), while for the 450K array (Fig. 4), the effect was largely confined to the Cy3 channel. We also noted that for the 450K design, in which the slides are laid out as a matrix of six rows by two columns, the Cy3 channel column 1 intensities were slightly higher than the corresponding column 2 intensities. As the Sentrix scanner is scanning arrays from row 1 onwards, the lowered fluorescence is not consistent with photodegradation. Instead this could possibly be due to the flow-through chambers in the Infinium XStain protocol, where XStain reagents flow from the last row to the first row (Melinda Ziino, personal communication).

The position bias should particularly affect Infinium II probes, as unbalanced falls in cyanine dye intensity will directly translate to biases in the methylated or unmethylated signal. The evident dye bias by array position implies that correction for dye bias is important in removing a source of batch-effects and that across slides, experimental factors of interest should not be structured by rows (and columns for the 450K array design).

### Preprocessing methods and the removal of position and dye bias

A total of 12 preprocessing methods were used to normalise the Infinium BeadChip data. Some of these methods incorporate dye bias correction within the process and some can accept data previously corrected for dye bias. The normalisation methods fall into three broad groups: raw preprocessing, within-array and between-array methods. Raw preprocessing directly translates Cy3 and Cy5 intensities into methylation and unmethylation signal, so there is no correction for probe design or dye bias. Within-array methods consider factors such as probe type and background correction and adjust each array individually. The six methods used were Subset-quantile Within Array Normalisation (SWAN) [23], Exponential–Normal mixture signal intensity background correction (ENmix) [24], Beta MIxture Quantile dilation (BMIQ) [25], Normal–exponential out-of-band convolution (noob) background and dye correction [26], SWAN performed on noob-corrected data (noob + SWAN) and similarly, BMIQ on noob-corrected data (noob + BMIQ). The between-array methods consider similar factors to within-array methods but seek to make the set of arrays identical in statistical properties by conditioning the data using techniques such as quantile normalisation. Applying quantile normalisation results in methylation arrays all scaled to have the same mean methylation and unmethylation signal. This large moderation in signal is best suited to circumstances where the biological variation in DNA methylation across arrays is modest, with most probes invariant. When the global invariance assumption is not met, larger global changes in distribution will be lost and false positives can be introduced from erroneous adjustment of features. The five between-array methods used were standard Illumina preprocessing [27], Dasen [28], Dasen performed on noob-corrected data (noob + Dasen), ENmix with subsequent quantile normalisation (ENmix—Quantile) and Functional normalisation [29].

For the ten methods that process and return methylation and unmethylation channel data, we examined the removal of position or dye bias (Fig. 4, Additional file 5: Fig. S5). We found obvious bias with raw preprocessing, while all the other normalisation approaches greatly improved the data quality. The within-array normalisation SWAN method randomly selects discrete subsets of Type I and Type II probes with similar CpG content, performs subset quantile normalisation on these subsets and interpolates the remaining probes to define new intensities. The SWAN method increased the methylation mean signal and dispersion to be nearer that of the unmethylated channel, which removes a large amount of position bias. The noob method uses normal–exponential convolution for background correction on DNA methylation arrays using the out-of-band fluorescence intensities of Type I probes on the array, as well as the control probes. This greatly increases the amount of data to model background fluorescence. The noob method was observed to compress the intensity data with the unmethylated signal having a mean and dispersion similar to the original Cy3 signal. The noob methylated and unmethylated signals appeared more uniform in comparison with SWAN-normalised data. This equalisation removes bias as the formation of M values or β is less impacted by dye or position. The combination of SWAN and noob was subtle with marginally more methylated signal dispersion. The ENmix preprocessing method models the methylation signal intensity with a flexible exponential–normal mixture distribution, together with a truncated normal distribution to model background noise and is an extension of robust multi-array-average (RMA) and noob. ENmix increased the mean and dispersion of the methylated signal to be more like the Cy5 signal, resulting in removal of much of the position bias.

For the between-array normalisation methods, we found Illumina preprocessing method scales all the fluorescence intensities from arrays towards an array from row 1. This scaling removes obvious position and dye bias from the data with a small amount of dispersion in array means remaining. Dasen combines background adjustment with a between-array normalisation method that is applied separately to methylated and unmethylated channels further subdivided into Type I and Type II probes. Dasen does not perform dye bias correction, so it is reasonable to also normalise data previously corrected by noob. Dasen moderated the means of all the arrays to be the same so no dispersion of BeadChip means remained. A large difference in fluorescence intensity still existed between the methylated and unmethylated channel but this disappeared with the use of Dasen in combination with noob. The ENmix authors developed a quantile normalisation method which can be run on ENmix corrected data. This hybrid ENmix-quantile normalisation approach, like the Dasen and noob combination, also equalised the methylated and unmethylated fluorescence intensities. Functional normalisation is typically coupled with prior noob background correction. The method extends quantile normalisation and only removes variation explained by a set of covariates associated with technical variation, such as the first two principal components of Infinium control probes. This PCA-based approach has similarities to Harman batch-effect removal but is more limited in scope. Functional normalisation returned results similar to Illumina preprocessing; however, there was less position bias.

In summary, the use of preprocessing to remove probe type, background and dye bias was highly effective in removing much of the obvious glass slide position bias, with methylated and unmethylated signal largely equalised. The means of the methylated and unmethylated signals could be further moderated together using between-array normalisation methods.

### Global methylation overview and batch-effect model specification

**Fig. 5:**
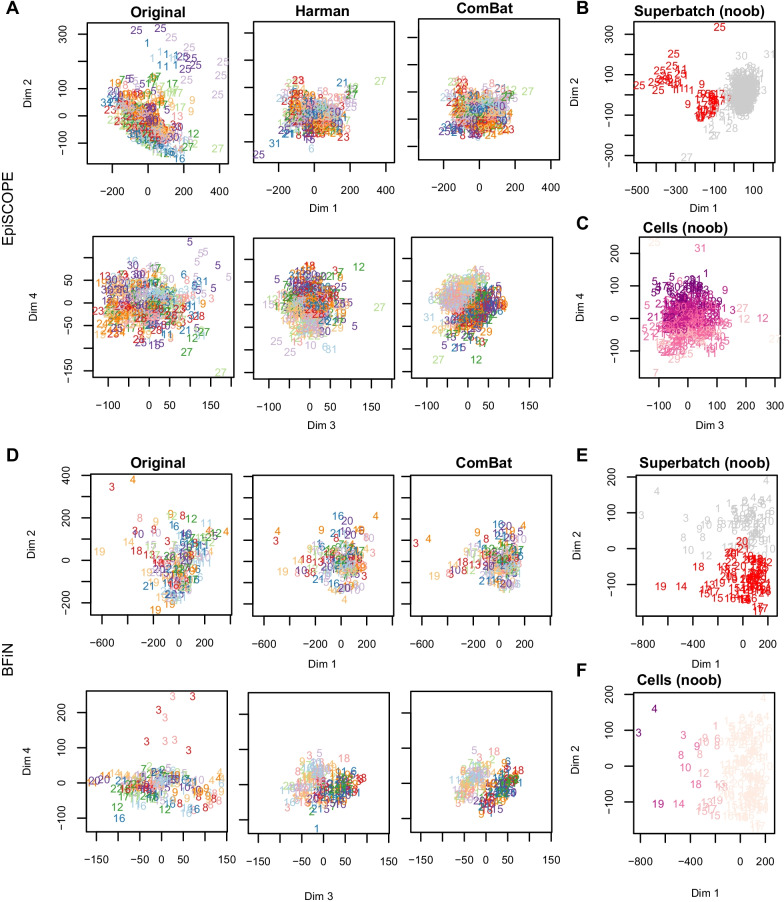
Principal component analysis of the EpiSCOPE and BFiN data. PCA was conducted on the original raw preprocessed M values and again after correction via Harman or ComBat. The data are presented with the number and colour signifying BeadChip slide identifier and the bold and pastel shading signifying male and female gender, respectively. PCA was also conducted on noob preprocessed data and coloured by slides of note across processing runs (those slides highlighted in gold in Fig. 3), or estimated cellular fraction. For the EpiSCOPE data (**a**), dimensions 1 and 2 of the PCA plots show the data to separate by slide. This was particularly evident in slides 1 and 25 and less so for slides 9 and 17. Arrays from slide 5 separated out discretely on dimension 4. The PCA plots of Harman or ComBat corrected data show the absence of data separation by slide; instead the corrected data show a strong separation by gender in principal dimensions 3 and 4, despite the data being limited to autosomal probes only. Separation of the data by DHA supplementation (experimental treatment) was not apparent in the principal components examined. **b** Consistent with the control probe findings, the 450K slides with high technical variation (slides 1, 5, 9, 17, 25) are the first arrays processed in each processing run. **c** Some separation of the data on the fourth dimension by the estimated proportion of neutrophils in the blood sample was observed. In the BFiN data PCA analysis (**d**), there was not obvious separation of the raw preprocessed data by slide identifier on dimensions 1 and 2. However, slide 3 clearly separated out on dimension 4. Batch correction via Harman or ComBat was sufficient to remove the separation of slide 3. The PCA plots of noob preprocessed data illustrate the two largest factors influencing the autosomal probes; **e** the eigenvalues for dimension 2 showed two clouds of samples—one for slides 1–12 and the other, slides 13–22 and **f** cellular composition—with saliva samples containing a higher immune cell component separating out on dimension 1. Within each of these two clouds there was further structure, with samples from some slides clustering together. For the BFiN data, the technical (batch) variation is largely due to processing run (superbatch) and less so, the individual slides

Unsupervised learning via principal component analysis (PCA) was used to examine global methylation patterns and determine the impact of different normalisation methods on the data, to gauge the influence of biological factors and to identify batch-effects (Fig. 5). Coordinated increased DNA methylation due to X chromosome inactivation in females (reviewed in [12]) is often a large source of correlation across the samples, so to allow easier inspection of technical effects in the data, we removed all X and Y chromosome probes from the PCA analysis. Both the PCA analysis and batch correction used M values. Post-correction, the M values were converted to β via a logit transform.

To identify and remove batch-effects using Harman or ComBat, a batch variable needs to be declared to the algorithm, as well as sources of biological variance that the algorithm should seek to retain. The EpiSCOPE data PCA analysis illustrated that arrays are often separating in groups composed of the sets of 12 arrays on the same glass slide, so we may consider this to be a ‘batch’. The superbatch structure would be each of the six processing runs. With the balanced design of the experiment, there was a near equal representation of gender and DHA supplementation across each slide. For Harman, the experimental variable to keep was declared as a compound variable of gender with DHA supplementation, while with ComBat, gender and DHA supplementation were the two covariates specified in the linear model. For both batch-correction methods the largest source of technical variation to remove was slide number (Fig. 5a, b). A minor impact of blood cellularity could be found in PCA dimension 4 of the noob-normalised EpiSCOPE data (Fig. 5c).

The BFiN data PCA analysis showed the greatest structure in the data due to technical effects was the processing run (Fig. 5d, e) and that cellular composition of the samples was a large source of biological variance and separated the data across PCA dimension 1 (Fig. 5f). Harman supports only categorical factors, so this cell component estimate was used to cut the samples into two groups; low and high immune component with a cut point at 6% estimated immune cell fraction. The BFiN data also had a balanced design across the slides by gender. For Harman, gender and the two immune component groups were combined as a compound variable specifying the biological variance to keep. With ComBat, gender and the immune component specified as a continuous variable were given to the linear model. Like with the EpiSCOPE data, for both batch-correction methods the source of technical variation specified to remove was slide number. Removing other sources of technical variance were also tested including using superbatch as the batch variable, or a combination of first removing variance associated with superbatch and then slide number. Specifying just the slide number was found to be sufficient to also remove the superbatch structure (analysis not shown).

### Removal of batch-effects

As observed from PCA plots (Fig. 5a, d), Harman and ComBat were highly successful in removing the batch-effect across samples. Both methods removed the separation of the data by slide (technical variance) and importantly did not remove the separation of declared biological factors of interest (such as gender and cell composition). Next, for each normalisation method, the amount of batch correction made to individual β values was examined as a measure of method performance. A β difference matrix was computed by taking the difference between the batch-corrected and original β matrixes, and then, for each individual CpG probe the absolute value of the interval between the minimum and maximum β difference was calculated. Large values in this maximal probe-wise β difference statistic highlight probes where one or more batches required a large amount of correction relative to others. The distribution of the maximal probe-wise β difference statistic across CpG sites can be examined to understand the effect of the various normalisation methods. Further adjustment made by batch-effect correction software is on the residual technical variance left after preprocessing. We propose that superior normalisation approaches should have more probes with smaller maximal probe-wise β difference statistic, as this suggests reduced technical variance across all batches.

**Fig. 6:**
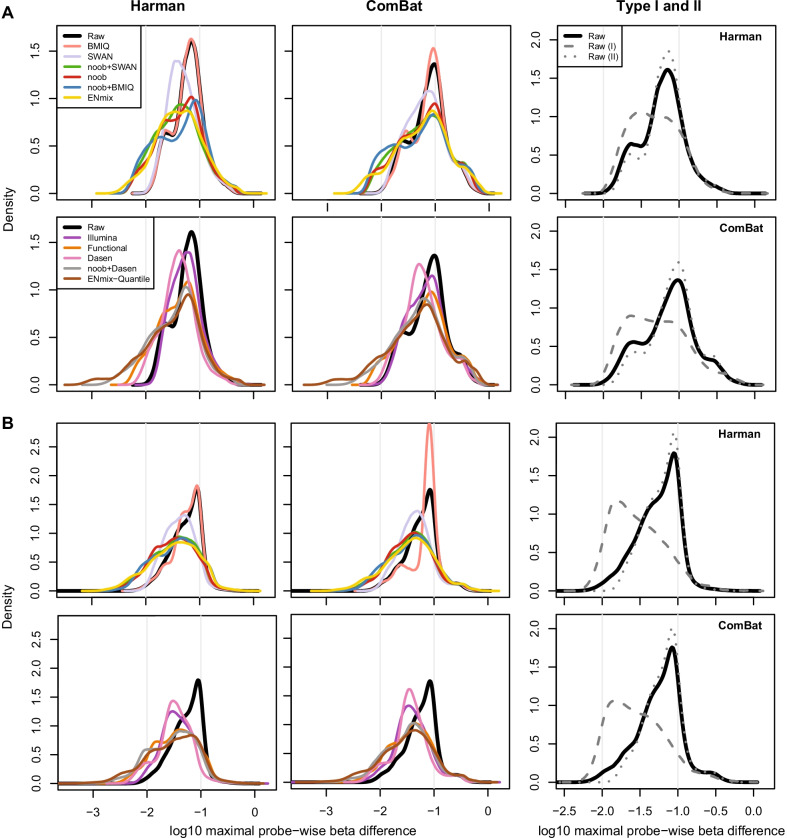
A global analysis of batch-effect corrections made after various preprocessing. Across each probe in the EpiSCOPE (**a**) and BFiN study (**b**), the maximal probe-wise beta difference after batch-effect correction was determined and a density plot constructed. The area under the curve illustrates the maximal level of adjustment across the probe distribution. The two vertical lines highlight probes with 10% and 1% maximal probe-wise beta difference (− 1 and − 2, respectively, in log scale). This is consistent with the segmentation used in Table 1. The rightmost column illustrates that if raw preprocessed data are subset into Type I (grey dashes) and Type II (grey dots) probes, many of the Type I probes had less batch-effect correction adjustment

On both the 450K and EPIC array formats, Type I probes were found to require far less batch correction than Type II probes (Fig. 6). This fits with expectation, as Type II probes are far more prone to colour bias and by extension, position bias. The maximal probe-wise β difference statistic was used to group the probes into those requiring low (< 0.01 β), moderate (between 0.01 and 0.10 β) and high (> 0.10 β) levels of adjustment for batch-effect on one or more slides. ComBat more aggressively adjusted the data with more probes in the high adjustment group across the two array designs and for all the preprocessing methods, except for the Illumina preprocessing method on the EPIC array, which was approximately equivalent (Table 1, Fig. 6).

**Table 1:** Beta differences after batch-effect removal across and datasets and preprocessing methods

Dataset	Method		Maximal probe-wise β difference		Groups (%)		
	Correction	Normalisation	Mean	Median	Low	Moderate	High
EpiSCOPE (450K)	Harman	Raw	0.074	0.062	0.1	80.4	19.5
		Illumina	0.074	0.055	0.0	81.8	18.1
		noob	0.065	0.045	7.3	75.1	17.6
		SWAN	0.064	0.046	0.1	85.0	14.9
		noob + SWAN	0.061	0.040	8.4	75.4	16.1
		Functional	0.063	0.044	7.1	77.1	15.8
		BMIQ	0.073	0.062	0.1	80.8	19.2
		noob + BMIQ	0.072	0.050	10.3	66.8	22.9
		ENmix	0.067	0.041	8.0	73.1	18.9
		ENmix-Quantile	0.059	0.038	16.6	68.8	14.7
		Dasen	0.052	0.040	3.0	88.0	9.0
		noob + Dasen	0.055	0.038	14.0	73.2	12.8
	ComBat	Raw	0.100	0.079	0.2	63.9	35.9
		Illumina	0.100	0.069	0.2	68.1	31.8
		noob	0.100	0.065	6.1	60.4	33.6
		SWAN	0.096	0.066	0.1	69.2	30.7
		noob + SWAN	0.099	0.061	5.9	61.5	32.7
		Functional	0.101	0.066	4.8	62.9	32.4
		BMIQ	0.102	0.082	0.2	62.9	36.9
		noob + BMIQ	0.101	0.060	9.5	57.8	32.7
		ENmix	0.105	0.064	8.1	58.2	33.7
		ENmix-Quantile	0.094	0.052	12.8	60.2	26.9
		Dasen	0.083	0.055	0.9	76.0	23.1
		noob + Dasen	0.090	0.054	8.0	64.9	27.1
BFiN (EPIC)	Harman	Raw	0.065	0.060	1.8	71.6	26.6
		Illumina	0.053	0.038	5.0	75.6	19.4
		noob	0.049	0.034	18.6	61.2	20.2
		SWAN	0.052	0.042	1.3	83.8	14.9
		noob + SWAN	0.050	0.037	20.5	58.5	21.1
		Functional	0.051	0.036	17.8	60.3	21.9
		BMIQ	0.066	0.061	1.9	71.2	26.9
		noob + BMIQ	0.055	0.038	21.3	51.5	27.2
		ENmix	0.055	0.037	24.4	48.5	27.1
		ENmix-Quantile	0.050	0.033	31.1	47.5	21.5
		Dasen	0.041	0.033	14.7	78.1	7.2
		noob + Dasen	0.045	0.032	30.1	53.9	15.9
	ComBat	Raw	0.072	0.062	2.3	67.5	30.2
		Illumina	0.056	0.039	7.3	73.4	19.3
		noob	0.058	0.038	14.2	62.3	23.5
		SWAN	0.059	0.044	1.9	79.0	19.2
		noob + SWAN	0.058	0.039	16.6	59.8	23.7
		Functional	0.060	0.040	13.7	61.0	25.3
		BMIQ	0.081	0.077	2.2	62.5	35.3
		noob + BMIQ	0.062	0.040	19.1	52.7	28.2
		ENmix	0.062	0.039	22.3	49.4	28.4
		ENmix-Quantile	0.058	0.036	25.7	48.7	25.6
		Dasen	0.050	0.036	12.0	73.6	14.4
		noob + Dasen	0.055	0.036	21.8	56.3	21.9

It is expected that normalisation methods accounting for bias due to factors such as colour, position and probe type should need less correction for variation across slides. The within-array preprocessing methods of ENmix, noob and the combination of noob and SWAN or BMIQ were effective in shifting probes requiring a moderate adjustment with raw preprocessing to the low adjustment group (a shift from maximal probe-wise beta difference between 0.01 and 0.10 β to under 0.01 β). For the between-array methods, the noob and Dasen combination or the ENmix-Quantile methods were effective at increasing the proportion of probes requiring low adjustment. The use of Dasen (without the addition of noob) was particularly effective in reducing the number of probes in the high adjustment group (Table 1, Fig. 6).

For the remainder of our analyses, noob-normalised data were used. The noob method has good performance in correcting for batch-effect and underlies, or can be combined with, several other normalisation methods. It also makes few assumptions about the data and is a within-array method, so is suitable for experiments with high biological variation, where quantile normalisation might not be appropriate.

### Identifying clustered methylation

The distribution of β at particular CpG sites may be modal in nature, which results in a clustered methylation pattern across samples. In practice, clustered methylation profiles can be due to technical or biological factors, and batch correction should ideally remove the former but preserve the latter. As biological variance to keep is declared per sample, rather than per feature, batch-effect removal software is prone to erroneously attributing methylation clustering to technical factors. When the distribution of biologically relevant clustered methylation is unbalanced across batches, then batch-effect removal software will inappropriately seek to converge the means of each batch. It is important to identify the CpG sites subject to such erroneous correction as this adjustment can profoundly destroy biologically meaningful clustering of the data. Which CpG sites naturally exhibit clustered methylation in populations is not well characterised, so a data-driven empirical approach was employed to find them.

To investigate, an optimal univariate *k*-means clustering method was used to identify multi-modal features in noob-normalised data, with each cluster having at least 5 samples and a β difference between the cluster centroids of 0.1. When first defining the number of clusters for each probe, samples arising from slides with the greatest batch-effects (EpiSCOPE slides 1, 5, 9, 17, 25 and 30 and BFiN slide 3) were set aside to reduce the impact of technical factors inflating the number of clusters. For each probe, the optimal number of clusters (*k*) was identified using Bayesian information criterion (BIC). Next, the previously removed samples were added back and clustering was undertaken again using the previously determined value for k. For each multi-modal probe (*k* > 1), the association between gender, batch, superbatch and cell composition was examined using statistical tests (described in Methods). Clustered methylation can often be due to allele-specific methylation (ASM), so for autosomal CpG probes with 2 or 3 clusters, we examined if the assortment across clusters fit with expectations from population genetics by employing a test for deviation from Hardy–Weinberg equilibrium (HWE). For all the described tests, a stringent FDR-moderated cut-off of *p* = 0.001 was used for establishing associations.

For the EpiSCOPE 450K data (Additional file 6: Fig. S6), a total of 22,281 CpG probes were found to be clustered, with 19,816 CpG probes having 2 clusters and 2379 and 86 for 3 and 4 clusters, respectively. There were 11,733 (52.7%) of clustered probes in HWE with 6086 (27.3%) measuring CpG methylation at the site of a common SNP and another 1919 (8.6%) having the probed CpG site within 10 bp of a common SNP (SNP-proximal). As expected, often the probes in HWE also overlapped with those found to be SNP-mapping (4786 probes) or SNP-proximal (848 probes). Of the probes in HWE, 4045 could not be linked to a nearby SNP. Another 7337 (32.9%) probes were clustering in association with gender with the great majority of these positioned on the *X* or *Y* chromosomes (6595 and 378 probes, respectively). Only 281 (1.3%) of probes clustered due to variability in the immune cell component, while a total of 1477 (6.6%) probes associated with batch and none with superbatch. The association with batch was expected to be low as the cluster discovery stage excluded the most batch-effect prone slides (further details in Methods). A total of 2011 (9.0%) of probes were cross-hybridising probes documented previously [30]. Finally, 1539 clustered probes could not be associated with any of the factors examined.

The distribution for the 80,752 clustered probes in the BFiN EPIC data was 76,041, 4588 and 123 for clusters of 2, 3 and 4, respectively (Additional file 7: Fig. S7). A large proportion were linked to variability in the immune cell component, with 48,762 (60.4%) probes associated. A total of 69,001 (85.4%) clusters were in HWE. We expect this high proportion is not due to a link with ASM but is due to the small number of samples with a high immune cell content. With 48,491 (99.4%) of the cell component-associated probes having two clusters and largely a small number of samples in the second cluster, we can expect that HWE will be upheld as the data will have a distribution similar to that for a rare allele. Consistent with this expectation, almost all probes associated with cellular component are in HWE (48,528 in total). There were 10,703 and 6160 probes at the site of a common SNP or SNP-proximal, respectively, with strong overlap with the clustering SNP-associated probes in the EpiSCOPE data, with 4530 of 6086 (74.4%) common SNP probes and 984 of 1919 (51.3%) SNP-proximal probes also clustering in the BFiN data. A total of 8304 probes in HWE could not linked to a nearby SNP, which is around twice the number in the EpiSCOPE data. For the 10,386 (12.7%) of probes clustering in association with gender, 9723 and 439 probes were on the *X* or *Y* chromosomes, respectively, and 5,630 (76.7%) were also associated with gender in the EpiSCOPE data. Only 2 probes associated with batch (slide number), whereas 1481 associated with superbatch. A total of 1813 were cross-hybridising probes, and 476 remaining probes could not be associated with any of the factors considered.

### Evaluating batch correction for each probe

While several biological factors were found to be associated with the phenomenon of multi-modal methylation, these factors cannot be used with certainty to identify all erroneously adjusted probes. For example, well-studied metastable epialleles such as nc886 have a robust imprinting-like clustering pattern but are not associated with a proximal SNP, nor parent-of-origin such as with imprinting [20, 31].

To better characterise the biologically relevant clustering set, we employed an empirical technique to evaluate the performance of batch-effect removal algorithms on individual probes. The intention of batch-effect correction is to remove technical variance; it follows that if the process is working correctly, a reduction in the sample variance (or standard deviation) of features should be observed after correction. A simple reduction in total variance cannot be used as a metric, as batch correction may highly disrupt biologically meaningful clustering but will reduce the overall dispersion of the data. Instead, the metric needs to be generalised for probes with either a unimodal or multi-modal distribution. For this purpose, we created a cluster-aware dispersion metric.

For the CpG features which form a multi-modal distribution, partial sums of squares were computed for each individual cluster using the dispersion from the mean for the members of a given cluster. The partial sums of squares were then collated across clusters to form a sample variance (as described in Methods). This modified calculation of sample variance will account for both the removal of assumed technical variance by Harman or ComBat and the preservation of a biologically meaningful clustering pattern. The log-base-2 of the ratio of sample variance precorrection over post-correction (log-variance ratio, LVR) was used as an empirical measure that batch correction was performing on multi-modal probes as intended. An LVR > 0 signifies that variance was inflated for a given probe after the application of the Harman or ComBat methods, whereas an LVR < 0 demonstrates that batch-effect correction is reducing the variance as intended.

**Fig. 7:**
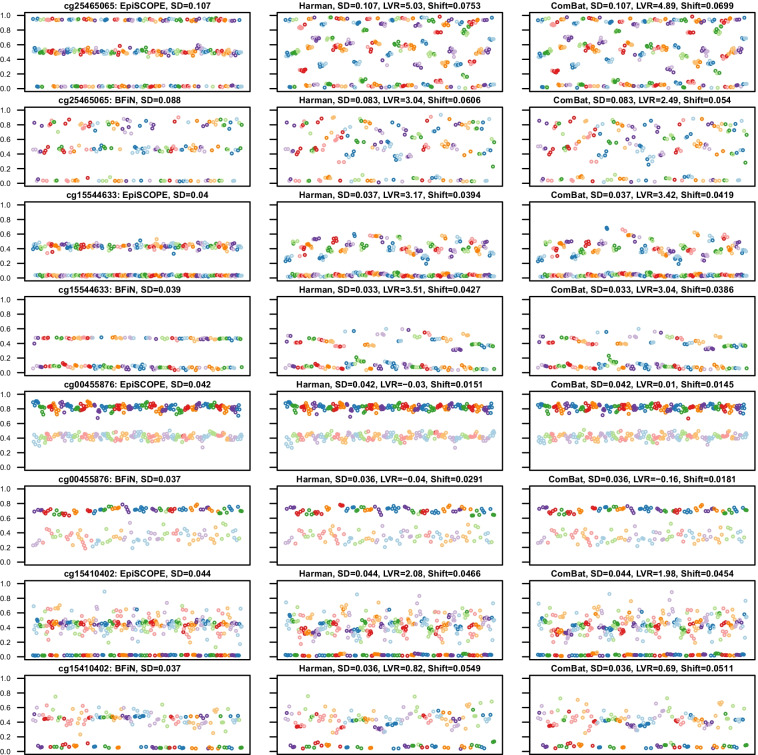
Biologically meaningful methylation clustering. Some probes with biologically meaningful methylation were erroneously corrected. Four example probes are illustrated. Each scatter plot compares methylation across slides (*X* axis) with the methylation *β* value (*Y*-axis). The datapoints are from each of the 369 Beadchip arrays, with the data sorted and coloured by slide number. The panel is ordered column-wise from left to right as original, Harman-corrected and ComBat-corrected data. It was observed that the standard deviation (SD) of the data remained the same or less; however, the log-variance ratio (LVR) was elevated considerably above 0. The mean *β* shift (Shift) is the mean change in *β* across all the 369 arrays induced by erroneous batch correction. The mapping of common SNPs falling within CpG sites can be used to identify CpG sites which should not be batch corrected. An example of this the probe cg25465065, which has the common C/T SNP rs3768276 positioned at the cytosine and as expected, the frequencies in each cluster are consistent with expectations of the Hardy–Weinberg equilibrium. However, the methylation as measured by probe cg15544633 on chromosome 2 is clustered to two groups: intermediate methylation and no methylation. This clustering is not in Hardy Weinberg equilibrium (*p* = 9.520 × 10^−9^), yet the clustering is likely influenced by genetics as the common SNP rs2516834 is immediately adjacent to the assayed CpG site. In the example with the Y chromosomal probe cg00455876, there is clearly a higher methylation state in males and this is clustering is still apparent after batch correction as gender was declared as biological variance to preserve. However, more complex gender associations may arise, in which batch-effect correction performs poorly. One of the alleles for the *X* chromosomal cg15410402 probe is inactivated in females, but the methylation state in males is complex, with almost half of the males having intermediate methylation and half no methylation. This may well be due to an interaction between gender and genetics, likely due to the influence of the commonly deleted sequence 5’-GGAGCTAGGCCG (rs66532084) 12 bp upstream from the measured CpG site

We observed that in instances of unintended removal of biologically meaningful methylation clustering (Fig. 7), such as arising from ASM (examples being probes cg25465065 and cg15544633), batch correction reduced the overall dispersion of the data (resulting in a smaller standard deviation) but greatly increased the LVR statistic above 0. However, in cases where the clustering had a biologically meaningful association with gender (such as probe cg00455876), the prior declaration of gender to the batch-effect algorithm as variance to preserve was sufficient to keep the LVR near 0. We also uncovered more complex methylation clustering associations with both gender and genetics (cg15410402). Despite the batch correction algorithm seeking to preserve gender-associated variance, the dual associations led to erroneous correction and an LVR above 0.

**Fig. 8:**
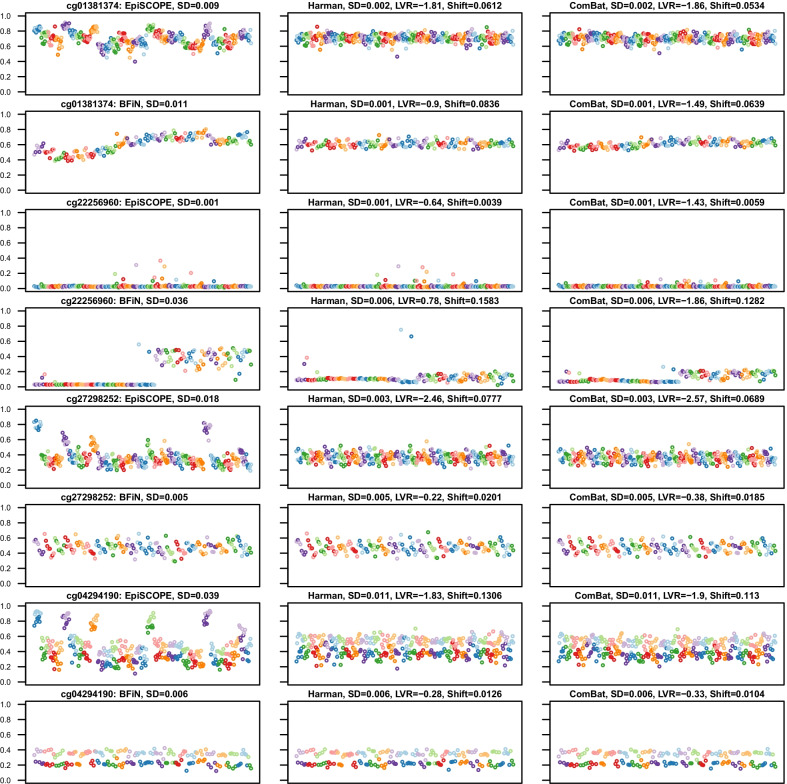
Examples of probes exhibiting obvious batch-effect. In some instances, probes had clear batch-effects which were corrected by application of Harman or ComBat. The panel layout is consistent with that in Fig. 7, with four examples of batch-effect prone probes illustrated. After batch correction, the SD was typically reduced and the computed LVR was considerably less than 0. Typically, as is the case with cg01381374, the particular influence of these probes on the data was idiosyncratic to the dataset. In other instances, there was high technical variance in one dataset but not the other. In the case of cg22256960, the batch-effect is limited to the EPIC superbatch 2 data. The example of cg27298252 highlights that batch-effect can be found both across arrays and by the position in the array. In particular, the EPIC data illustrate clear positional bias. The cg04294190 probe demonstrates that both technical and biological factors can contribute to methylation clustering. In this case, the data are clustered both by gender and within the 450K data, by slide number

In instances where a probe had batch-effects removed as intended, both the overall standard deviation (SD) was lower, as well as the LVR falling below 0. Figure 8 provides various examples of the type of batch-effects discovered in the data. These include probes with obvious batch-effects in both the 450K and EPIC data (cg01381374), to those limited predominantly to one of the sets (cg22256960), to slide-based batch-effects in one but showing a positional batch-effect in both (cg27298252), and clustering associated with both gender and batch-effect (cg04294190).

The above examples illustrate the need for a discovery-based approach. We found the LVR statistic was particularly useful at identifying CpG sites having biologically relevant clustered methylation that should not be subjected to batch-effect correction (erroneously corrected probes). When applying the LVR statistic to identify probes prone to batch-effect (batch-effect susceptible probes), we found that fully methylated or unmethylated CpG sites often had very small corrections made to them by Harman or ComBat, but given the initial small variance, this resulted in large changes in ratio. The LVR statistic was combined with the mean difference between corrected and uncorrected data to correlate changes in variance and shifts in beta values (Figs. 7, 8). A shift in beta cut-off was introduced to focus towards those probes with a large shrinkage in LVR and an appreciable difference to β after correction.

**Fig. 9:**
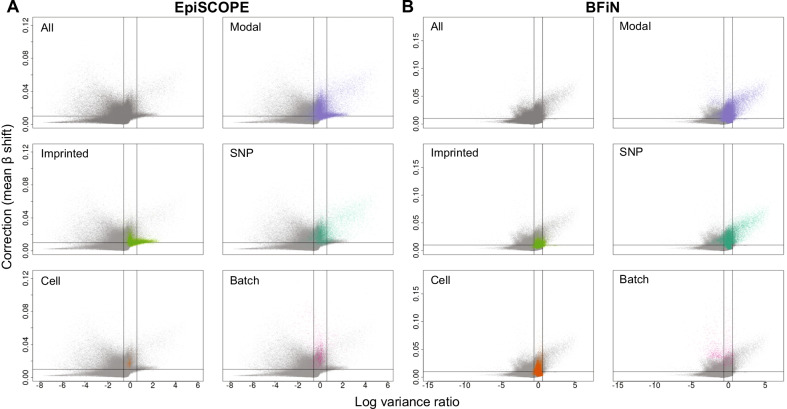
Isolating erroneously corrected and batch-effect susceptible probes via Log-variance ratio and mean *β* shift. For all probes in the **a** EpiSCOPE and **b** BFiN datasets, the change in variance after batch correction (expressed as log-variance ratio) relative to the degree of batch correction (expressed as mean β shift) was plotted. The same data were then highlighted for clustered probes which were modal in distribution. These modal probes were in turn subdivided into probes which were modal and associated with imprinting, a single nucleotide polymorphism at the measured CpG site, cellular component and batch-effect. The vertical lines are plotted at LVR of 0.584 and − 0.584 and the horizontal line at a mean *β* shift of 0.01

To classify erroneously corrected and batch-effect susceptible probes, we used a cut-off of 50% variance increase (log2(1.5), LVR = 0.584) or decrease (log2(1/1.5), LVR =  − 0.584), respectively, and mean β shifts of at least 0.01. How these cut-offs interacted with known clustered methylation associations was also characterised (Fig. 9). As anticipated, modal probes with a known common SNP at the measured CpG site often had LVRs much greater than 0 and larger mean β shifts. In the intersection between the 427,274 probes shared across the EpiSCOPE and BFiN datasets, there were 31,449 erroneously corrected and 7,478 batch-effect susceptible probes common to both datasets.

### Validation

Analysis of our unconfounded high-quality 450K and EPIC data established two sets of probes: one that was prone to batch-effect and another erroneously modified by batch-effect algorithms. It is useful to further explore the generality of these sets. Inclusion of further data from different studies and geographies may allow discovery of methylation clustering associated with less common SNPs or certain populations, and batch-effect susceptible probes needing significant correction in multiple datasets. To explore the universality of our findings, we searched for additional large 450K and EPIC datasets with these selection criteria: (1) hundreds of samples and an EWAS design, (2) with raw IDAT files in the public domain, having (3) the original Illumina Sentrix identifiers to enable the study of slide and positional effects, with (4) the experimental variable(s) and gender made known, such that biological variance to preserve can be declared to the batch-effect software, also (5) with reasonably subtle phenotype to make technical factors obvious and finally (6) the slide identifier and biological phenotype not being overly confounded.

For the 450K array validation set, we selected an 845 participant (657 women and 188 men) methylation sub-study of peripheral blood as part of the European Prospective Investigation into Cancer and Nutrition (EPIC-Italy), a molecular epidemiology project on diet and cancer [32]. For EPIC array validation, we selected two US originating datasets: 536 EPIC arrays prepared from buccal DNA samples from infants born less than 30 weeks postmenstrual age, as part of the Neonatal Neurobehavior and Outcomes in Very Preterm Infants (NOVI) Study [33], and 392 arrays from cord blood and peripheral blood at 7 years old from 196 children, as part of the Urban Environment and Childhood Asthma (URECA) study [34]. In the NOVI dataset, two arrays were removed: one with a truncated and corrupted IDAT file and another had > 5% failed probes. What remained were data from 235 female and 299 male neonates. The URECA data also had one array removed due to > 5% failed probe count leaving data from a total of 192 female and 199 male children.

The 450K EPIC-Italy cohort was divided across 72 slides. The control probes suggested a reduction in the quality of the data after slide 28, with many slides showing unfavourable control probe intensities across the broad range of sample dependant and independent control sets (Additional file 8: Fig. S8). The NOVI array set was spread across 78 EPIC slides and had good results for the sample independent controls, but high signal in a subset of green negative control probes and a number of outlier fluorescent intensities in the specificity I and II controls. Collectively, this suggests some non-specific extension (Additional file 9: Fig. S9). The URECA data were composed of 49 slides with a well-balanced design, having gender and tissue split evenly across arrays. The control probes generally signified high quality, except for three slides (Additional file 10: Fig. S10).

PCA was used to identify slides subject to batch-effect and the influence of biological factors, such as cell composition. As before, when identifying the optimal k for each CpG probe cluster, the most batch-effect prone slides were excluded for this step. In total, 3, 19 and 25 slides were set aside for cluster identification in the URECA, EPIC-Italy and NOVI datasets, respectively. During batch-effect correction, the biological factors of interest for the EPIC-Italy dataset were specified as gender and neutrophil composition, for NOVI, gender and the proportion of immune component cells and URECA, gender and tissue source (cord or peripheral blood). In each of the three cases, the batch-effect variable specified was the slide identifier.

### Universality across datasets

The combination of mean β shift and LVR was used to identify probes shifted substantially by batch correction and also exhibiting appreciable decreases in LVR (batch-effect susceptible) and increases in LVR (erroneously corrected) (Additional file 11: Fig. S11). The same mean β shift of 0.01 and 50% change in LVR cut-offs as the EpiSCOPE and BFiN data were employed.

A total of 427,160 probes were cross-examined across the five datasets; this constitutes 452,453 probes common to all the datasets, less the 25,293 multi-modal distributed probes having a strong association (*p* = 0.001) with cell composition in one or more of the datasets. This cell composition set was removed as erroneous correction due to unbalanced within-batch sample cell composition is particular to each study and driven by the nature of the biosample. In particular, the BFiN saliva samples and NOVI buccal swabs are a mixture of epigenetically distinct immune and non-immune cells, resulting in a large increase in probes having a multi-modal distribution. While the cell composition differences were modelled and declared to the batch-effect algorithm, this may not be sufficient to prevent erroneous correction in all instances.

Lists of batch-effect susceptible and erroneously corrected probes were generated and compared across datasets. In total, 229,681 (53.8%) of probes are batch-effect susceptible in at least one of the datasets; however, only 15,094 (3.5%) are batch-effect susceptible in at least 4 of 5 datasets and 4,649 (1.1%) in all datasets examined (Table 2). The number of batch-effect susceptible probes unique to one of the datasets was proportional to the size of the dataset and the quality of the data control probe metrics (Additional file 12: Fig. S12). The high-quality BFiN dataset only had 17,922 such probes with 3169 (17.7%) unique to that dataset.

**Table 2:** Batch-effect susceptible and erroneously corrected probes across datasets

Set	Total probes	Number of datasets considered as batch-effect susceptible, total and percentage					
	n	0	1	2	3	4	5
*Batch-effect susceptible*							
All common probes	427,160	197,479 (46.2)	121,657 (28.5)	65,881 (15.4)	27,049 (6.3)	10,445 (2.4)	4649 (1.1)
Cross-hybridising	26,796	7446 (27.8)	5761 (21.5)	4692 (17.5)	3956 (14.8)	3097 (11.6)	1844 (6.9)
Multi-mappers	30,543	9092 (29.8)	6947 (22.7)	5575 (18.3)	4253 (13.9)	3032 (9.9)	1644 (5.4)
Type I	117,436	65,934 (56.1)	27,564 (23.5)	12,228 (10.4)	6445 (5.5)	3517 (3)	1748 (1.5)
Type I (≤ 2 internal CpG)	31,528	13,656 (43.3)	8528 (27)	4518 (14.3)	2779 (8.8)	1449 (4.6)	598 (1.9)
Type II	309,724	131,545 (42.5)	94,093 (30.4)	53,653 (17.3)	20,604 (6.7)	6928 (2.2)	2901 (0.9)
Type II (0 internal CpG)	131,010	43,578 (33.3)	42,855 (32.7)	27,389 (20.9)	11,862 (9.1)	3785 (2.9)	1541 (1.2)
Tm < 70 °C	7961	671 (8.4)	1426 (17.9)	2214 (27.8)	2424 (30.4)	1095 (13.8)	131 (1.6)
*Erroneously corrected*							
All common probes	427,160	418,405 (98.0)	8755 (2.0)	5202 (1.2)	2860 (0.7)	1620 (0.4)	856 (0.2)

A much smaller set was found for erroneously corrected probes, with 8,755 probes (2.0%) in at least one of the datasets, 1620 (0.4%) erroneously corrected in at least 4 of 5 datasets and 856 (0.2%) in all datasets examined (Table 2, Additional file 13: Fig. S13). Overwhelmingly, the erroneous adjustment of probes is related to multi-modal CpG site methylation arising from the influence of a genetic variant at the CpG site (see SNP-associated multi-modal probes in Additional files 6, 7: Figs. S6, S7). As such, it is expected that the identification of erroneously corrected probes is a function of (1) cohort size, (2) genetic distinctness of the population and (3) quality of the array data.

There were 992 probes unique to URECA, despite it having less than half of the cohort size of EPIC-Italy. The URECA cohort was composed of 75% African American, 20% Hispanic and 4% admixed participants. African ancestry populations are known to have more genetic diversity than non-African populations [35]. The 968 and 571 unique probes in the EpiSCOPE and BFiN cohorts are high given the cohort size. There were also another 1,490 probes shared exclusively between EpiSCOPE and EPIC-Italy (Additional file 13: Fig. S13).

The NOVI cohort is relatively large (*n* = 534) and has a multi-ethnic genetic background with 22% African American, 7% Asian and 7% Hawaiian or Pacific Islander background. However, the number of unique probes (144) was the lowest of all the five cohorts (Additional file 13: Fig. S13). We posit this is due to data quality and use of buccal cell biosamples, with the large variances in the data masking some genetic effects on the data. In support of this notion, we note that NOVI had the highest number of probes (359) not shared with the four other cohorts. This can be interpreted as the clusters being harder to identify.

Finally, we note that of 856 probes common to all the datasets, according to dbSNP v151, 768 (89.7%) of these are directly at the site of a SNP and another 20 (2.3%) have the measured CpG site within 10 bp of a SNP.

### Lists of troublesome probes

With large EWAS designs, we suggest that beta values should be characterised empirically for clustering and for changes in variance before and after batch correction using the LVR statistic. However, this approach is not suitable for small studies as sufficient arrays are required to identify clusters. The need for larger datasets is magnified for ASM-associated clustering involving SNPs with small minor allele frequencies. Therefore, we provide reference matrices containing the LVR statistic and mean β differences (rounded to 4 decimal places) for all the arrays examined in this study (Additional file 15: Table S1). This list comprises data from 1214 450K and 1094 EPIC arrays from regionally diverse and multi-ethic populations across Australia, the USA and Italy and spanning multiple commonly collected biosamples (blood, buccal cells and saliva). This reference matrix will allow investigators to identify erroneously corrected and batch-effect susceptible CpG probes in their study, particularly for rarer SNPs in the population under inspection. For ease of use, this matrix is also distributed as part of the HarmanData package available on Bioconductor and can be called directly into an R session.

### The nature of probes more subject to batch-effects

Four factors were considered for batch-effect susceptibility: cross-hybridisation due to highly homologous sequences, Infinium design (Type I or II), the number of CpG sites internal to the probe and probe melting temperature (Tm).

Previously, Chen et al. identified a list of 29,233 probes in the 450K design which are highly homologous with 47–50 bases matching to a cross-reactive target [30]. Often this is a result of probes targeting repetitive genomic sequences or genes that have pseudogenes or homologous genes [36]. In a related exercise, Benton et al. mapped probe sequences to the human genome using BOWTIE2 [37] and identified 33,457 probes aligning greater than once [38]. There is a large overlap between the Chen and Benton set, with 21,361 probes shared between the sets. Both of these were overlapped with the sets of probes considered as batch-effect susceptible (Table 2). While these sets were minor contributors to the total number of probes batch-effect susceptible in 1–3 datasets, cross-hybridisation and multi-mapping were a large factor in probes batch-effect susceptible in 4 or 5 datasets and constituted 39.7% and 35.4%, respectively, of the 4649 probes found in all datasets.

Infinium design also held some influence. Type I and II probes constituted 1748 (37.6%) and 2901 (62.4%), respectively, of the probes universally considered batch-effect susceptible, whereas they were 27.4% and 72.6% of the total set of 427,160 probes. There is a significant bias towards Type II probes being batch-effect susceptible in all sets (Fisher exact *p* < 2.2e−16). Interestingly, the sets were enriched for those Type I probes having 2 or less internal CpG sites and Type II probes having no internal CpG sites (Table 2).

**Fig. 10:**
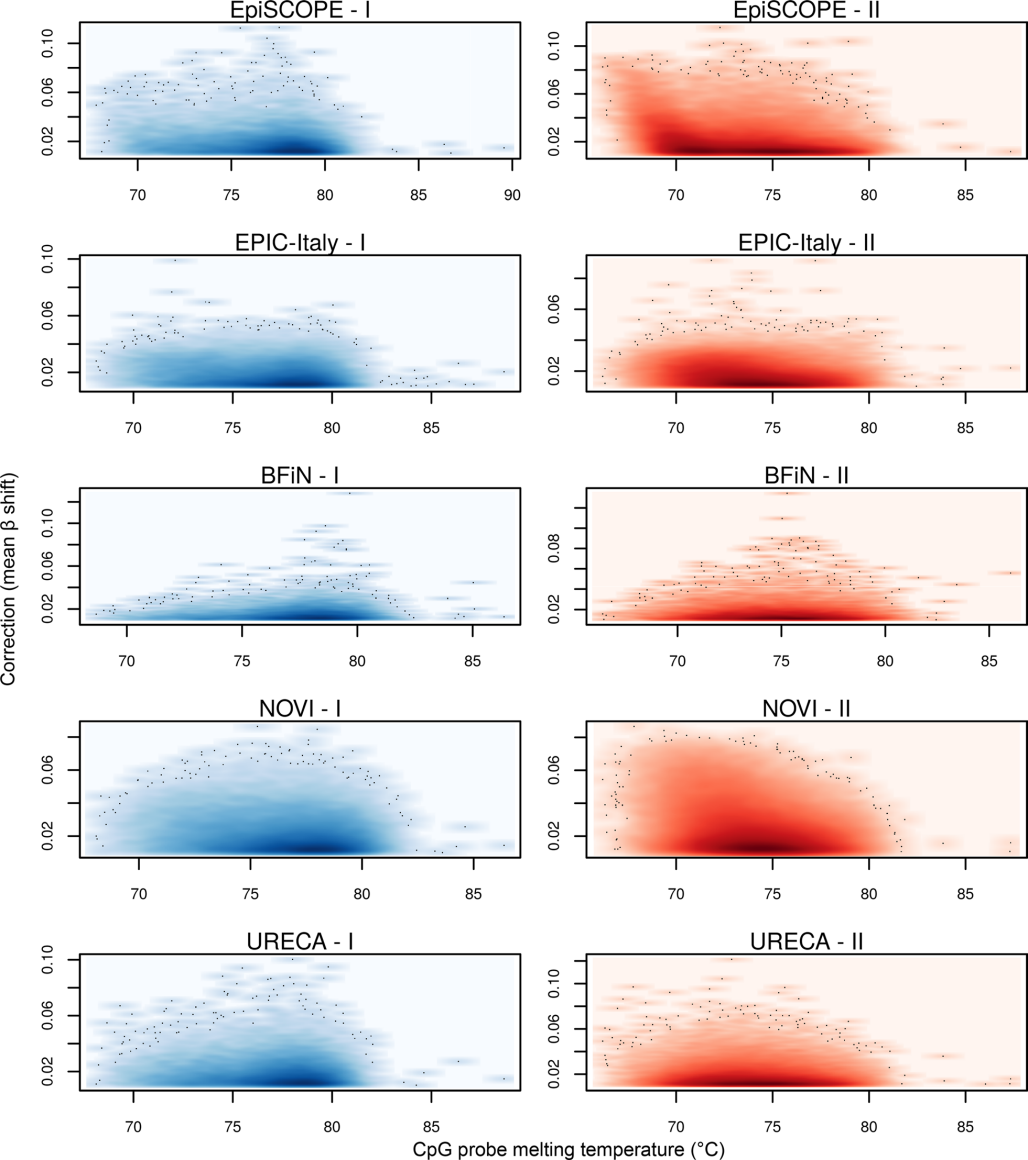
The influence of probe melting temperatures on batch-effect. For each of the five studies and two probe types (I and II), the relationship between probe oligonucleotide melting temperature (Tm) and batch correction (mean *β* shift) was examined. A subset of Type II probes in the EpiSCOPE and NOVI study data were observed to require more batch correction when the probe Tm is low

A relationship between sensitivity to a batch-effect and probe melting temperature (Tm) could also be found for the EpiSCOPE and NOVI datasets in particular (Fig. 10). While most probes have an in silico determined Tm of ~ 75 °C, the 7,961 probes (from the set of 427,160) with a Tm of less than 70 °C show high between-batch correction. The limitation to the EpiSCOPE and NOVI datasets might be due to factors such as a difference in salt concentration in the hybridisation buffer, the oven set temperature or fluctuations from that, or the length of hybridisation time. Interestingly, for the EpiSCOPE set, the sets requiring the most correction were the first set processed each day (communication with service provider).

In summary, probe batch-effect susceptibility is multi-factorial in nature and was strongly associated with all the four factors examined. We note the low Tm set is largely different to the cross-hybridisation and multi-mapping sets, with only 198, 98 and 414 probes shared between the Benton, Chen and all sets, respectively. The set of batch-effect susceptible probes described here has overlap with the previously published sets described by Chen et al. and Benton et al. but is discrete.

### Findings of false positive probes in published EWAS studies

To gauge the impact in the literature of batch-effects on the outcomes of EWAS studies, a search was conducted across PubMed for appearances of the 4649 probes identified as batch-effect prone in all the 5 studies examined here. The R library easyPubMed (2.13) was used to query titles and abstracts in PubMed [39] for the term ‘EWAS’ in conjunction with one of these probe identifiers. In total, 3 studies were found.

In an EWAS considering Parkinson's disease, Moore et al. [40] listed cg11963436 as one of the top-12 loci and interestingly found the locus to also replicate using Sequenom EpiTYPER for 219 individuals. We observe this probe to show profound batch-effects in the EpiSCOPE and URECA data (Additional file 14: Fig. S14). Another EWAS from Chen et al. [41] considering reduced kidney glomerular filtration rate in 567 HIV-positive and 117 HIV-negative men describe cg18368637 as one of the top-3 loci. For this probe, there are obvious batch-effects across the 5 datasets we examined (Additional file 14: Fig. S14). Finally, in a recently published re-analysis of existing gestational diabetes mellitus (GDM) EWAS data, Liu et al. [42] found cg22385669 to be one of 62 significant CpG methylation sites and 1 of the 6 probes in their SVM model predicting GDM occurrence. This probe has a more subtle batch-effect than the previous two examples but is still readily apparent (Additional file 14: Fig. S14).

All three examples had batch-effects readily observable via visual examination in the 5 datasets considered here. This illustrates the importance of plotting β ordered and coloured by slide number as a diagnostic to find potentially spurious associations.

## Discussion

The Illumina HumanMethylation array is a popular epigenomic technology, and certainly the technology of choice for EWAS given the coverage, cost per unit and overall accuracy of the methylation calls. However, there are known small subsets of probes which are problematic and may give rise to false positive or negative associations [30, 38].

Using high-quality data with a balanced-block design from the EpiSCOPE and BFiN cohorts, this study sought to identify which probes are prone to batch-effects, to investigate the factors that contribute to batch-effects and to characterise the effects of various preprocessing steps and batch-effect procedures. We found that batch-effects are predominantly linked to larger processing batches but using slide identifier as a batch variable is sufficient to capture and correct for batch-effect. Despite the arrays being run in service facilities by experienced operators using liquid handler robotics, multi-channel pipettes and other methods to control for uniformity, some batch-effects are persistent. There is also technical variance associated with position on each slide, but this can be largely managed by appropriate preprocessing methods.

Across the two studies, we identified sets of batch-effect prone probes, the optimal preprocessing steps to reduce batch-effects and the factors that may have contributed to the technical variance observed in the data. The nature of the batch-effect was different in each dataset, with many of the troublesome probes not overlapping. To validate and further generalise our observations, we considered a further three large EWAS datasets from the literature. Consideration of all five datasets confirmed that the nature of the batch-effect was different in each experiment; however, there was a consistent set of 4,649 batch-effect prone probes that needed a high amount of correction. Alarmingly, we found three published EWAS studies that had reported one of these batch-effect prone probes as a top CpG site of interest.

For future studies, to assist investigators in eliminating batch-effect prone probes we provide reference data characterising the batch-effects observed across the 2308 450K and EPIC arrays investigating in this current study. This reference data will have utility for anybody undertaking an EWAS. Acknowledging that the exact character of batch-effects is unique to each study, troublesome probes are not provided as a final list, rather a set of reference data specifying the moderation required for each probe across each of the five studies considered here. This matrix will allow simple identification of probes commonly exhibiting high levels of technical variance across multiple studies, including new studies.

Also, as part of this investigation, we also uncovered and characterised a set of probes which should not be batch-effect corrected. These erroneously corrected probes have biologically meaningful clustering of methylation which cannot be declared a priori to current batch-effect removal tools, and this biological variance is erroneously attributed as technical variance. While it is common to remove probes associated with SNPs [12], we demonstrate that empirical measures are needed to identify all probes with biological variation that should be preserved and provide a statistic to undertake this. This statistic takes the clustering into account, and after batch-effect correction, the statistic is applied a posteriori to determine the sets of erroneously corrected and batch-effect susceptible probes. This procedure can be undertaken for any study, with the limitation that the study must be of sufficient size for the clusters to be identified. Smaller studies can instead filter on our list. We also suggest the list is a useful reference to discover novel regions in the epigenome with profound methylation clustering due to biological factors other than a proximal SNP or association with gender, such as metastable epialleles [20, 31].

This work also illustrates a process to minimise technical variation in methylation data. In agreement with prior recommendations, we suggest the first and foremost step to address this is careful study design, where the biological factors of interest are distributed across batches [1]. Next, an optimal preprocessing method should be used to remove as much technical variation as possible. We find noob to be a good generalisable choice which makes little assumptions on the data. If a between-array normalisation suits the study design, Dasen is particularly effective in reducing the proportion of probes requiring a high amount of downstream correction, with ENmix-Quantile, and noob in combination with Dasen, for increasing the proportion of probes requiring very little correction. In deciding whether to perform within-array or between-array normalisation methods, it is possible to use an empirical testing framework such as Quantro [43].

Many comparisons of Infinium preprocessing methods exist in the literature. The value of this current comparison is the focus on technical variance in an EWAS context. EWAS often consider subtle biological phenotypes with small effect sizes, so technical variation (batch-effects) can be particularly troublesome. In studies with large between-group effects, such as cancer and normal tissue, some batch-effect can be tolerated as the biological variance is much greater than the technical variance. The metric used to compare preprocessing methods was maximal probe-wise β difference, a measure of residual technical variance remaining after processing. There was some overlap with the findings of previous comparisons. Shiah et al. compared 11 preprocessing methods on a large 450K prostate cancer dataset and considered technical replicate variances and differences, within-batch clustering and inter-array correlations. They found Dasen and noob to be preprocessing methods which minimised technical differences [44]. Liu and Siegmund [45] compared 9 methods across 4 datasets and found the combinations of noob with SWAN or BMIQ were optimal within-array methods and functional normalisation, subset quantile normalisation (SQN) and Dasen optimal between-array methods. Recently, another comparison of 8 methods across 2 datasets found ENmix and noob offered the highest correlation between duplicates, lowest absolute differences and smallest deviations from true methylation levels [46].

If batch-effect correction is to be employed after preprocessing, then multi-variate visualisation, such as PCA or multidimensional scaling, should be used to understand global patterns in the data and determine the largest sources of covariance. Also, as recommended by others [47], cell-composition analysis, if possible, should be undertaken on the methylation data. Uncovered sources of biological variance from these analyses will inform specification of the batch-effect correction model. After batch-effect correction, the corrected data should be compared with the original data at a probe-wise level to determine the set of erroneously corrected probes. For this set, it is appropriate to use the original data instead of corrected data. We present LVR, a statistic that robustly identifies erroneously corrected probes. Finally, we provide examples of the effects of inappropriate correction, and incidences in the literature of likely false positive results due to the absence of batch-effect correction.

We show that the ComBat and Harman batch-effect removal methods are largely comparable at the global scale. ComBat offers advantages in more complex model specification and allows for multiple categorical or ordinal variables, whereas Harman only allows a single categorical variable. Harman can remove or preserve multiple factors in one pass using compound variables. In principle, Harman should preserve more biological variance as it optimises for maximal preservation of biological variation when removing batch-effect. Consistent with the conservatism of Harman, we find that ComBat has more probes with maximal probe-wise β difference in the high moderation set (> 10% β moderation between batches). Over-correction can potentially introduce false positive findings. It has been found that while ComBat could remove contrived batch-effects introduced into randomly generated data, it also resulted in profound inflation of Type I error in certain use cases. In balanced or random designs, the degree of false positive results was reduced by increasing sample numbers. However, the unbalanced simulation showed increasing false positive results with increasing sample size [48]. This is also in agreement with earlier cautionary work advocating that ComBat can introduce false positive results with uneven study designs [49]. Harman has not been tested in such comparisons.

Given the caveats and potential shortcomings identified here of applying batch-effect correction to EWAS data, some investigators may dismiss the use of batch-effect correction. However, if no correction is applied, this leaves a study open to false positives as a result of batch-effect. Investigators might consider using post hoc analyses, such as Cook’s distance (Cook’s D), instead of batch-effect correction to identify spurious associations driven by outlier datapoints with high leverage. Cook’s D is particularly effective at identifying spurious associations as a result of rare ASM events due to the large β change within individuals harbouring that SNP. However, batch-effects are typically more subtle with smaller changes in β and all the arrays within a batch moving as a group. Therefore, some spurious associations are likely to be missed by Cook’s D and other diagnostic tests which detect highly influential observations.

## Conclusions

Illumina Infinium BeadChips are empowering large epigenome-wide association studies in human populations. The technology is largely robust, but not ideal. We find that despite utilising various forms of bias removal and normalisation, some batch-effects persist. This residual batch-effect is associated with the day of processing, the individual glass slide and the position of the array on the slide. The individual array features most affected differed substantially across each of the five datasets considered. We also find probes that are erroneously adjusted by the correction procedure. We provide reference matrices of (1) CpG probes which are prone to high technical variance (batch-effect) and (2) CpG probes which are erroneously adjusted by batch-effect removal approaches. In addition, we suggest workflows and statistics which can help identify these two sets of probes in any moderately sized study.

While it is important to limit technical variance and false positive results in a study, batch-effect removal approaches should be used with some caution and employ post hoc diagnostics to understand the impact of the correction and identify any erroneous correction of particular sets of probes. The best time to consider the control of technical factors is during the experimental design phase. If a study highlights differential methylation in probes requiring substantial correction, these putative CpGs should be validated by a second independent methylation quantification approach, such as next generation sequencing, pyrosequencing or MALDI-TOF mass spectrometry.

## Methods

### Participant characteristics and data acquisition

Participant characteristics for DOMInO samples in the EpiSCOPE study with 450K methylation data are described in detail elsewhere [20], as is the EPIC methylation study of Body Fatness and Cardiovascular Health in Newborn Infant samples [22]. Some of the EpiSCOPE phenotype and methylation data are also present on the NCBI Gene Expression Omnibus (GEO) service with accession GSE89278. EPIC-Italy, NOVI and URECA study data can be downloaded from GEO with accessions GSE51032, GSE128821 and GSE132181, respectively.

### Sample preparation and processing

The EpiSCOPE HumanMethylation450 (450K) arrays were processed by the Australian Genome Research Facility Ltd (AGRF). DNA extracted from Guthrie card blood spots was bisulphite converted by the Zymo EZ DNA Methylation kit (Zymo Research, Orange, CA, USA). All arrays with hybridised for 18 h at 48 °C with the oven temperature monitored with data loggers to ensure there were no temperature fluctuations during the hybridisation step. All arrays were scanned with same Illumina iScan® System. Extension and staining steps of the bead chip protocol were performed with a Tecan liquid handler robot.

The BFiN cohort isolated DNA from infant saliva samples collected at birth using the Oragene OG-250 kit (Genotek). DNA was bisulphite converted and Human MethylationEPIC (EPIC) arrays processed at the Australian Cancer Research Foundation (ACRF) Cancer Genomics Facility in Adelaide, South Australia.

For EPIC-Italy samples [50], DNA was extracted from buffy coats or blood cell fractions via the QIAsymphony DNA Midi Kit (Qiagen, Crawley, UK) with 500 ng of DNA bisulphite converted using the Zymo EZ-96 DNA Methylation-Gold Kit and 450K arrays processed by the Human Genetics Foundation (HuGeF) in Turin, Italy.

In the NOVI study [33], genomic DNA was extracted from buccal swab samples, collected near term-equivalent age in neonates, using the Isohelix Buccal Swab system (Boca Scientific). DNA samples were plated randomly across 96-well plates and the DNA converted using the Zymo EZ DNA Methylation kit and EPIC arrays processed by the Emory University Integrated Genomics Core.

The URECA [34] samples were from frozen paired cord blood mononuclear cells (CBMCs) and peripheral blood mononuclear cells (PBMCs) at age 7 and DNA extracted using a Qiagen AllPrep kit (QIAGEN, Valencia, CA) with EPIC arrays run at the University of Chicago Functional Genomics Facility (UC-FGF).

### Preprocessing

All of the Infinium methylation data across the projects was reprocessed for this current work from raw IDAT files, with data analysis performed in the R statistical computing environment (4.0.3). All the scripts for the entire analysis are available in a Github repository (https://github.com/JasonR055/Batch_correcting_methylation_data). The EPIC-Italy and NOVI study originating data were downloaded from the Gene Expression Omnibus (GEO), with the Bioconductor GEOquery library (2.58.0) used to download the phenotype data and the raw IDAT files downloaded via FTP. All files were preprocessed and normalised using Bioconductor (3.12). The binary IDAT files were read into R using minfi (1.36.0) with preprocessing via the raw, SWAN, noob, Illumina and Functional normalisation methods implemented in minfi, the Dasen and BMIQ methods from the package wateRmelon (1.34.0) and ENmix from ENmix (1.26.6). Cell composition estimates as the authors of the EpiDISH package (2.6.0) used this for their reference generation. The ‘epidish’ method within the EpiDISH library was used to estimate the fractions of immune and epithelial component of the blood, saliva and buccal samples. We used the blood fraction and epithelial, fibroblast and immune component reference files supplied by the authors in their paper [51]. BMIQ-normalised data were used as input as this was consistent with the reference files.

Small sets of probe values were also moderated to handle particular rare edge cases. For example, it is possible for raw, Illumina, Dasen and Functional normalisation to produce values of exactly 0 for one or both of the methylated (Meth) and unmethylated (Unmeth) signals. When processed into betas (*β* = Meth/(Unmeth + Meth)) or *M* values (M = log2(Meth/Unmeth)), Meth = 0 yields *β* = 0 and *M* =  − ∞, and Unmeth = 0 yields *β* = 1 and *M* = ∞. If both Meth and Unmeth signals are 0, then *β* and *M* are recorded as missing. The other preprocessing methods slightly moderate 0 values, yielding *β* approximating to 0 or 1 and non-infinite *M* values. For consistency across the preprocessing steps, values were modified to eliminate the generation of infinite *M* values. After normalisation, *β* were extracted and shifted from exactly 0 or 1, to approximate 0 or 1 by the ‘shiftBetas’ function in Harman. If both Meth and Unmeth signals were 0, these were changed to *β* = 0.5, such that *M* = 0 when transformed using the ‘logit2’ function of minfi. Missing *β* were also converted to *β* = 0.5 and upon logit transformation will become *M*-values of 0. This is consistent with the approach of SWAN. A very small amount (sd = 1e−8) of random normally distributed noise was also added, as features having every *M*-value exactly 0 will terminate ComBat with an error due to the variance also being 0. This strategy was also used for the EPIC array probe cg06180910, which was found to have every M value being 0.

### Batch correction

For batch correction, the R implementation of Harman (1.18.0) and the ComBat function in the Bioconductor library sva (3.38.0) were applied to data resulting from each normalisation method. For each, the default settings were used. For Harman, the default confidence limit of 0.95 implies that Harman will remove technical variance across each slide, with the probably of also removing biological variance arising from gender and treatment effects on DNA methylation constrained to 0.05. Harman corrected PCA values were transformed back into corrected M matrices using the ‘reconstructData’ function. For both Harman and ComBat, the returned corrected M matrices were transformed into β values using the ‘ilogit2’ function in minfi.

### Detection of modal CpG probes

An optimal univariate clustering method provided in the ‘Ckmeans.1d.dp’ R library (4.3.3) was used to discover multi-modal features. In the initial cluster discovery step, to reduce the impact of technical effects inflating the number of clusters for each array feature, samples from the 450K slides 1, 5, 9, 17 and 25 were removed and similarly, slides 14–22 left out for the EPIC data. The Bayesian information criterion (BIC) statistic was used to select the two most optimal cluster numbers (*k*). The data were then re-clustered with the top two values of k and specifying that each cluster must have at least 5 samples with a minimum distance between cluster centroids of 10% methylation (*β* difference of 0.1). The largest value of k meeting these criteria was retained. A total of 8 and 11 CpG probes on the 450K and EPIC arrays, respectively, had computed optimal values of k above 4. These were manually recoded as *k* = 4. Clustering was then repeated with the inclusion of all samples and using the optimal value of *k* for each probe.

### Statistical testing of corrected data

Modal CpG probes were examined for associations with several biological factors and batch-effect. To examine the association between cell composition and clustering, a simple linear model was used on the estimated proportions of neutrophils for blood data and total immune system cells for saliva or buccal swab data. The associations between clustering and gender, batch and superbatch were examined with Fisher exact tests. In the instance of superbatch and 450K data, Chi-square tests with simulated p values were used instead due to the computational limitations of Fisher exact on larger contingency tables. For autosomal CpG probes on with 2 or 3 clusters, departure from Hardy–Weinberg equilibrium was also examined using an exact test from the ‘HardyWeinberg’ R library (1.7.1). The *p* values for each test were moderated for multiple testing using the false discovery rate (FDR) procedure of Benjamini and Hochberg [52] implemented in the R method ‘p.adjust’. A conservative FDR-moderated *p* value < 0.001 was used to classify a probe as associated with a factor. This cut-off was determined after visual examination of the distributions of the returned p values. Modal probes were further annotated as being on chromosome X or the site of a common single nucleotide polymorphism (SNP). The ‘snp151Common’ table from UCSC was used for SNP annotation. This is table of SNPs from dbSNP (version 151) having a minor allele frequency of at least 1% in any of five super-populations. Also, matches to the list of potentially cross-hybridising as determined by Chen et al. [30] were recorded.

The sample variance in β for each probe was calculated for the original and Harman and ComBat corrected data. In instances where the methylation data for a given probe were clustered (*k* > 1), the sample variance was calculated by adding together the sum of squares for each cluster and then dividing by the total number of samples, less one.

### Estimation of probe melting temperature

The ‘calcTm’ function within the Bioconductor ‘HELP’ library (1.48.0) was used to estimate the melting temperature (Tm) for each probe in the 450K and EPIC designs. This function uses the nearest-neighbour melting temperature estimates of Allawi et al. [53] but does not also include correction for mono- or divalent cation concentrations.

Infinium I has A and B probes, and the Tm for each was calculated and averaged. Infinium II probes will contain a degenerate R (purine) base opposite cytosine in a CpG context. So, Tms were calculated where all R bases were A or G, respectively. From these two Tm values, an average probe Tm was calculated.

## Supplementary Information

**Additional file 1: Figure S1.** EpiSCOPE study blocking plan. Across the 31 slides, 29 were completely balanced for gender with 6 male (coloured in powder blue) and 6 female (pink) replicates, while slides 30 and 31 were partially balanced with 5 females to 7 males and 5 females to 4 males, respectively. With regards to DHA supplementation, slides 1–23 and slide 30 were completely balanced with 6 replicates having DHA supplementation (square) and 6 controls (circle), with slides 24–29 having 5 controls to 7 DHA supplemented and slide 31, 5 controls to 4 DHA supplemented. Within a slide, the samples order was randomised to avoid correlation of position with gender or DHA supplementation.

**Additional file 2: Figure S2.** Body Fatness in Newborns (BFiN) study blocking plan. Across the 22 slides, 15 were completely balanced for gender with 4 male (coloured in powder blue) and 4 female (pink) replicates. Slides 5, 6, 13, 14, 19 were partially balanced with 5 females and 3 males, while slide 12 had 3 females and 5 males. Slide 22 had the 3 remaining samples. The blocking was also structured by percentage bodyfat. As this variable is continuous, the samples were mapped to yield approximately equivalent distributions across slides. On the figure, the lowest, middle and upper tertiles of percentage bodyfat are represented by a downward-facing triangle, a lozenge and upwards-facing triangle, respectively. Within a slide, the samples order was randomised to avoid correlation of position with gender or percentage bodyfat.

**Additional file 3: Figure S3.** Full set of control probes for the EpiSCOPE study. Further detail on the controls is provided by Illumina in the BeadArray Controls Reporter Software Guide document.

**Additional file 4: Figure S4.** Full set of control probes for the BFiN study. Further detail on the controls is provided by Illumina in the BeadArray Controls Reporter Software Guide document.

**Additional file 5: Figure S5.** BFiN fluorescence intensity slide positional effect is reduced with preprocessing methods. Infinium green (Cy3 dye) and red (Cy5 dye) fluorescent intensities are formulated into methylated (meth) and unmethylated (unmeth) signals. These meth and unmeth signals are used to calculate β and M values. If the 169 Beadchips in the BFiN set are grouped by row (R) position on the glass slide, there is evidence that the distribution of fluorescent intensities is associated with position. As for the EpiSCOPE dataset, this effect diminishes with preprocessing methods (see Fig. 4).

**Additional file 6: Figure S6.** EpiSCOPE modal probe associations. Each probe identified as having a modal distribution within the EpiSCOPE data was tested for association with various factors. This upset plot groups the probes significant for each factor and by their most numerous intersections across factors. The factors considered were superbatch (Super), estimated cell composition (Cell), chromosome X or Y location (ChrX, ChrY), remaining batch-effect after the most batch-effect prone slides were removed (Batch), a common single nucleotide polymorphism at the CpG site (SNP) or within 10 bp proximal (SNP_10 bp), cross-hybridisation prone (Crosshyb), gender (Gender) and probes in Hardy-Weinberg equilibrium (HWE). A proportion of modal probes could not be associated with any factor considered (Unknown). The two most common intersections were modal probes on the X chromosome and associated with gender (presumably as a result of X chromosome inactivation) and CpG sites at the site of a common SNP having ratios consistent with the Hardy–Weinberg principle for expected allele frequencies.

**Additional file 7: Figure S7.** BFiN modal probe associations The upset plot for BFiN data. Refer to Additional file 6: Fig. S6 for further description of the factors.

**Additional file 8: Figure S8.** Full set of control probes for the EPIC-Italy study. Further detail on the controls is provided by Illumina in the BeadArray Controls Reporter Software Guide document.

**Additional file 9: Figure S9.** Full set of control probes for the NOVI study. Further detail on the controls is provided by Illumina in the BeadArray Controls Reporter Software Guide document.

**Additional file 10: Figure S10.** Full set of control probes for the URECA study. Further detail on the controls is provided by Illumina in the BeadArray Controls Reporter Software Guide document.

**Additional file 11: Figure S11.** Log-variance ratio and mean β shift plots for all five datasets. Refer to Fig. 9 for further description of the plots. The panels are the data from EpiSCOPE (**a**), BFiN (**b**) as presented in Fig. 9, as well as EPIC-Italy (**c**), NOVI (**d**), and URECA (**e**).

**Additional file 12: Figure S12.** Intersection of batch-effect susceptible probes across the five datasets. The upset plot presents the number of batch-effect susceptible probes per dataset as well as their intersections. A total of 4649 probes were common to all datasets.

**Additional file 13: Figure S13.** Intersection of erroneously corrected probes across the five datasets. The upset plot presents the number of erroneously corrected probes per dataset as well as their intersections. A total of 856 probes were common to all datasets.

**Additional file 14: Figure S14.** Three batch-effect susceptible probes reported as top EWAS findings. The three probes, (cg11963436, cg18368637, cg22385669), show obvious batch-effect in all the five datasets considered. Each scatter plot compares methylation across slides (X axis) with the methylation β value (Y-axis). The datapoints are the totality of the arrays from each study, sorted and coloured by slide number. The panel is ordered column-wise from left to right as original, Harman-corrected and ComBat-corrected data. In each instance, the standard deviation (SD) of the data was reduced, the log-variance ratio (LVR) was considerably below 0 and the mean β shift (Shift) was greater than 0.01.

**Additional file 15. Table S1.** Reference log-variance ratio and mean *β* shift values for the five datasets.

## Data Availability

The EpiSCOPE datasets analysed during the current study are available at the Gene Expression Omnibus (GEO) under accession GSE89278. For the BFiN study, the data are not available as the participants have not consented to this. GEO accessions for the three validation datasets are presented in the methods. Output data and methods for this work are available on Bioconductor via the Harman and HarmanData packages.
