# Supplementary figures and images for: Batch-effect detection, correction and characterisation in Illumina HumanMethylation450 and MethylationEPIC BeadChip array data
Source: Clin Epigenetics. 2022 Apr 29;14:58. doi: 10.1186/s13148-022-01277-9 (PMC9055778; doi:10.1186/s13148-022-01277-9)

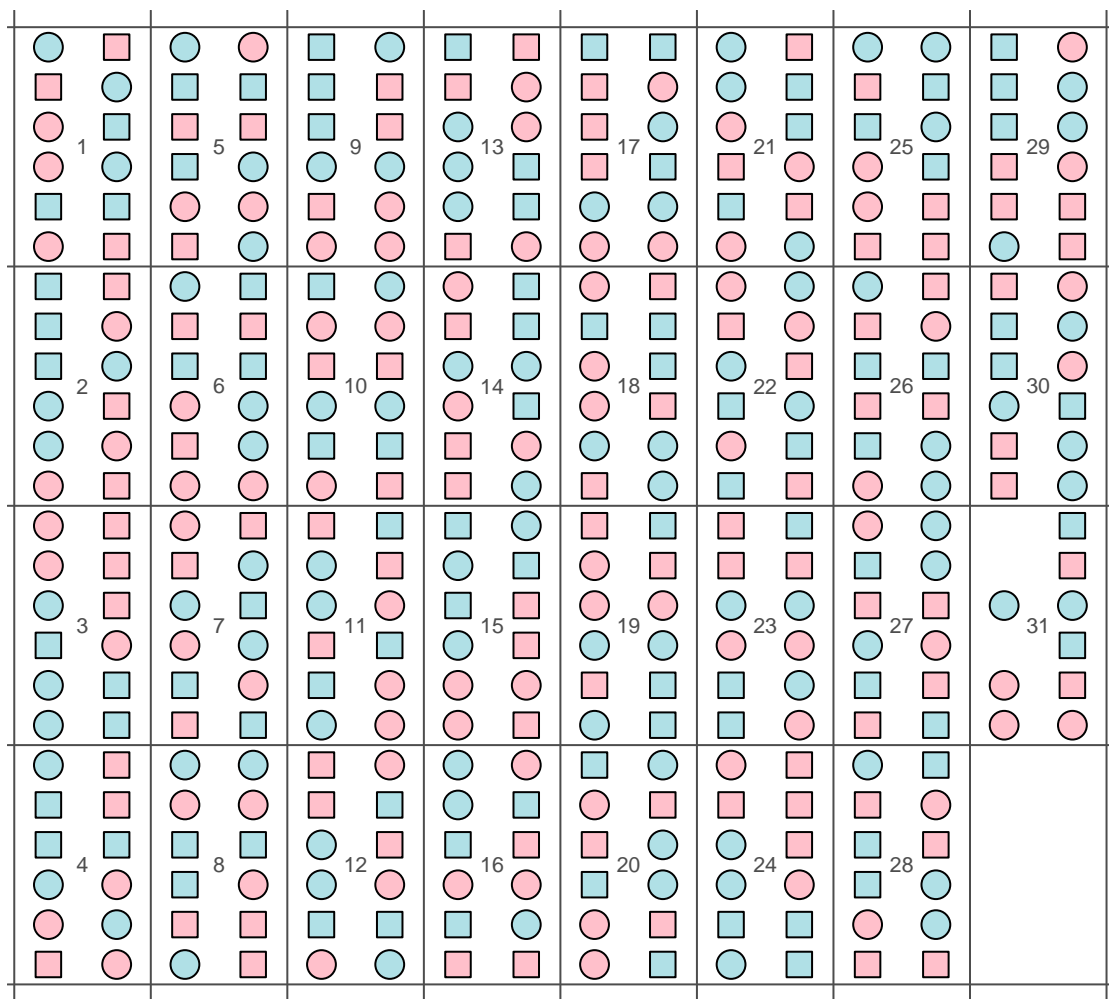

Supplement: Supplementary file 1 — Additional file 1: Figure S1. EpiSCOPE study blocking plan. Across the 31 slides, 29 were completely balanced for gender with 6 male (coloured in powder blue) and 6 female (pink) replicates, while slides 30 and 31 were partially balanced with 5 females to 7 males and 5 females to 4 males, respectively. With regards to DHA supplementation, slides 1–23 and slide 30 were completely balanced with 6 replicates having DHA supplementation (square) and 6 controls (circle), with slides 24–29 having 5 controls to 7 DHA supplemented and slide 31, 5 controls to 4 DHA supplemented. Within a slide, the samples order was randomised to avoid correlation of position with gender or DHA supplementation. [file 13148_2022_1277_MOESM1_ESM.pdf]

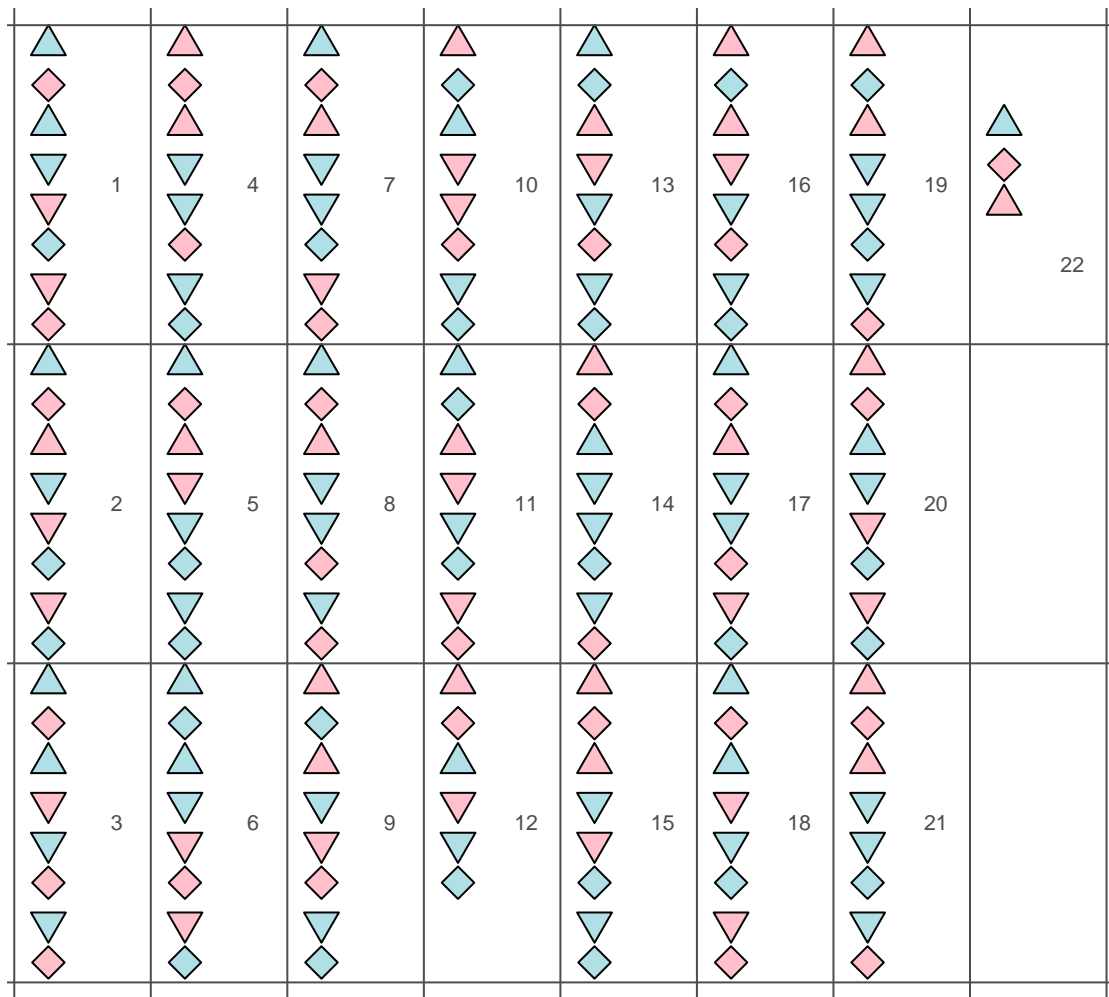

Supplement: Supplementary file 2 — Additional file 2: Figure S2. Body Fatness in Newborns (BFiN) study blocking plan. Across the 22 slides, 15 were completely balanced for gender with 4 male (coloured in powder blue) and 4 female (pink) replicates. Slides 5, 6, 13, 14, 19 were partially balanced with 5 females and 3 males, while slide 12 had 3 females and 5 males. Slide 22 had the 3 remaining samples. The blocking was also structured by percentage bodyfat. As this variable is continuous, the samples were mapped to yield approximately equivalent distributions across slides. On the figure, the lowest, middle and upper tertiles of percentage bodyfat are represented by a downward-facing triangle, a lozenge and upwards-facing triangle, respectively. Within a slide, the samples order was randomised to avoid correlation of position with gender or percentage bodyfat. [file 13148_2022_1277_MOESM2_ESM.pdf]

Fluorescence Intensity

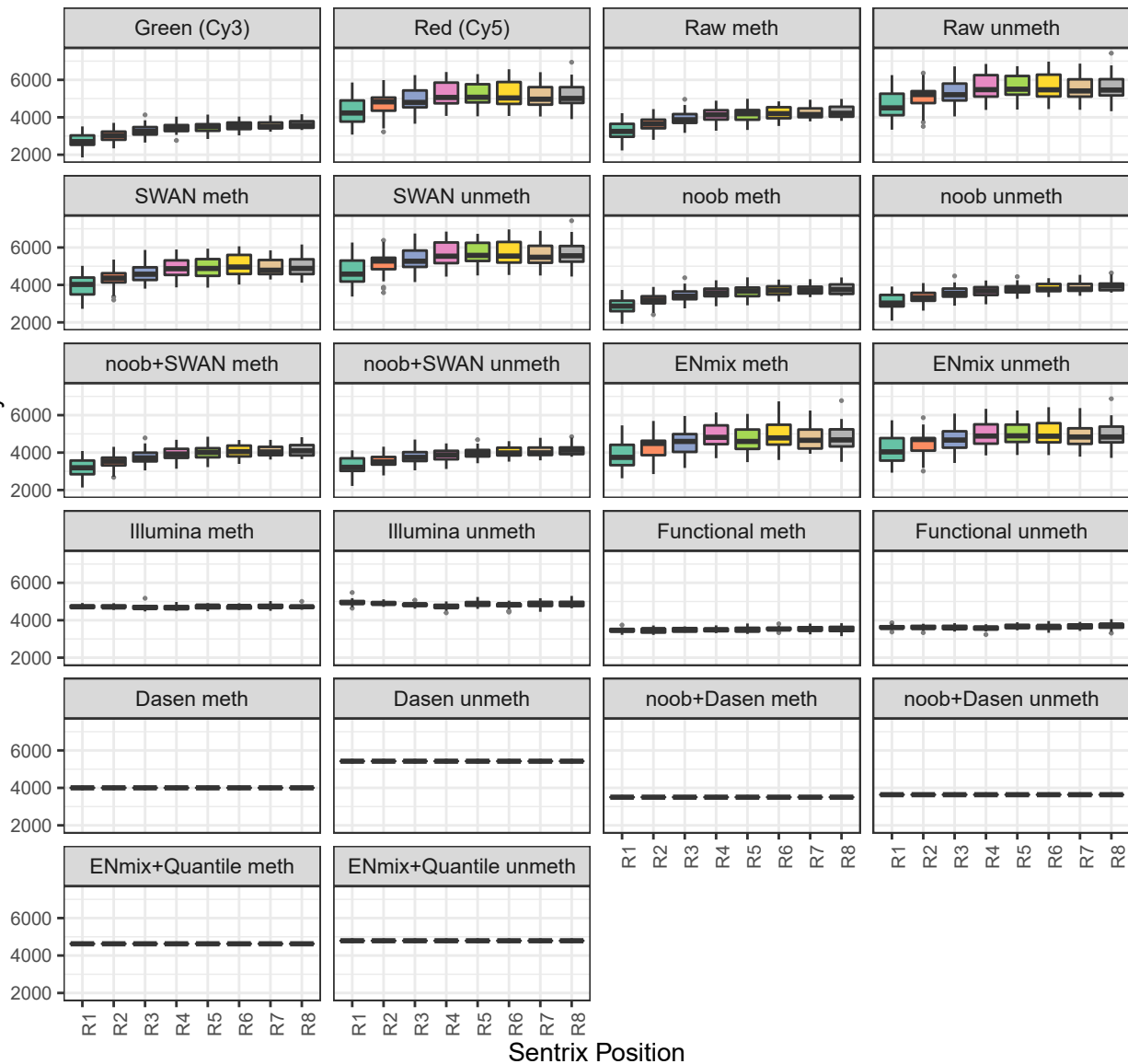

Position

R1 R2 R3 R4 R5 R6 R7 R8

Supplement: Supplementary file 5 — Additional file 5: Figure S5. BFiN fluorescence intensity slide positional effect is reduced with preprocessing methods. Infinium green (Cy3 dye) and red (Cy5 dye) fluorescent intensities are formulated into methylated (meth) and unmethylated (unmeth) signals. These meth and unmeth signals are used to calculate β and M values. If the 169 Beadchips in the BFiN set are grouped by row (R) position on the glass slide, there is evidence that the distribution of fluorescent intensities is associated with position. As for the EpiSCOPE dataset, this effect diminishes with preprocessing methods (see Fig. 4). [file 13148_2022_1277_MOESM5_ESM.pdf]

# EpiSCOPE

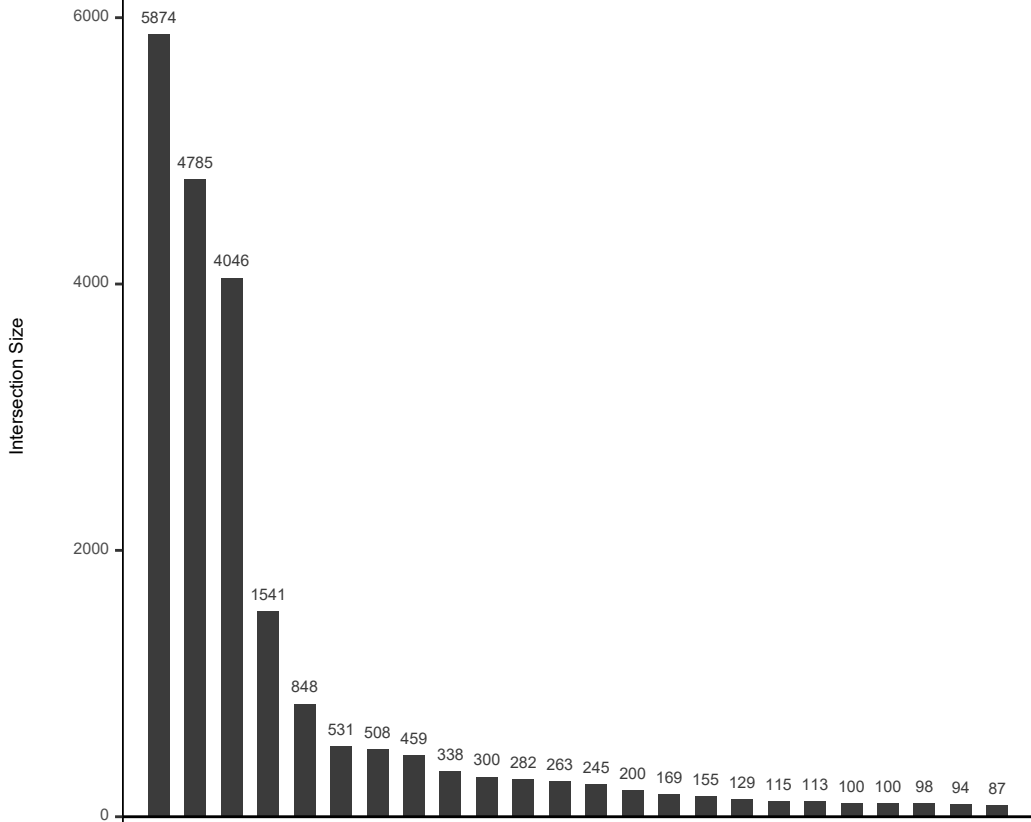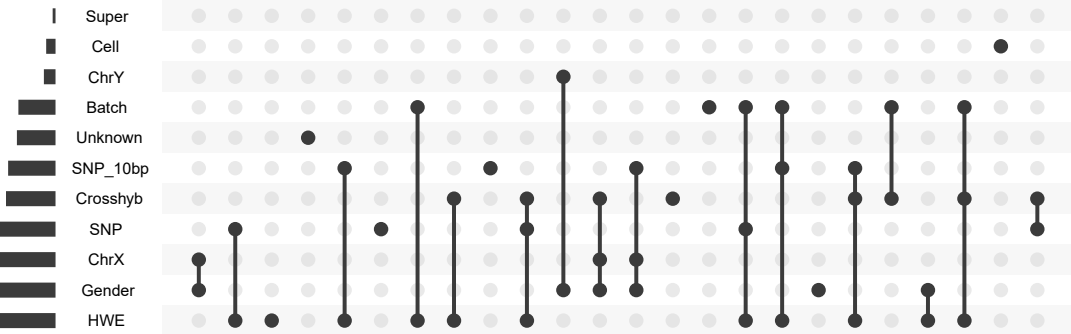

Set Size

12000 9000 6000 3000 0

Supplement: Supplementary file 6 — Additional file 6: Figure S6. EpiSCOPE modal probe associations. Each probe identified as having a modal distribution within the EpiSCOPE data was tested for association with various factors. This upset plot groups the probes significant for each factor and by their most numerous intersections across factors. The factors considered were superbatch (Super), estimated cell composition (Cell), chromosome X or Y location (ChrX, ChrY), remaining batch-effect after the most batch-effect prone slides were removed (Batch), a common single nucleotide polymorphism at the CpG site (SNP) or within 10 bp proximal (SNP_10 bp), cross-hybridisation prone (Crosshyb), gender (Gender) and probes in Hardy-Weinberg equilibrium (HWE). A proportion of modal probes could not be associated with any factor considered (Unknown). The two most common intersections were modal probes on the X chromosome and associated with gender (presumably as a result of X chromosome inactivation) and CpG sites at the site of a common SNP having ratios consistent with the Hardy–Weinberg principle for expected allele frequencies. [file 13148_2022_1277_MOESM6_ESM.pdf]

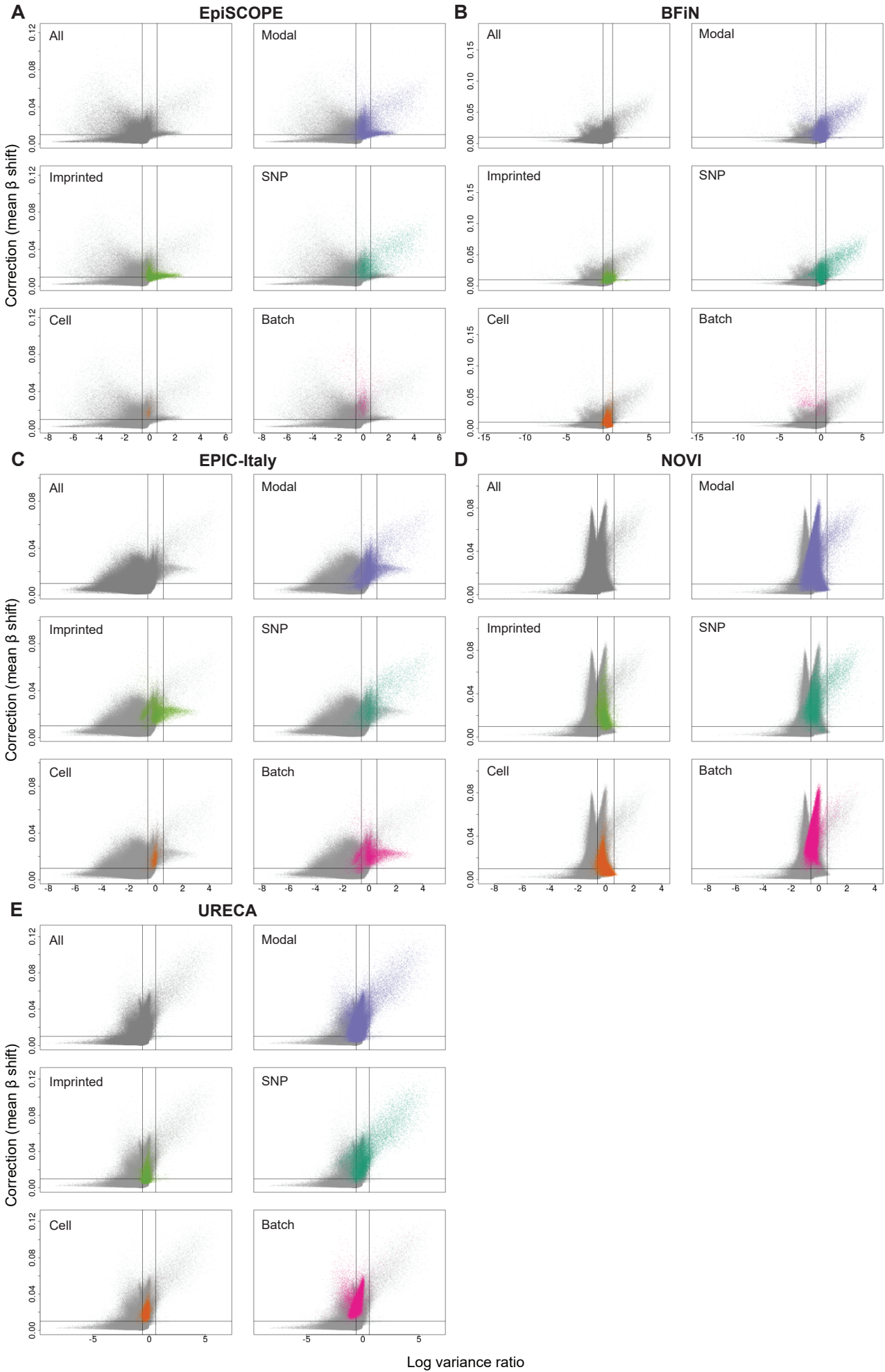

Supplement: Supplementary file 11 — Additional file 11: Figure S11. Log-variance ratio and mean β shift plots for all five datasets. Refer to Fig. 9 for further description of the plots. The panels are the data from EpiSCOPE (a), BFiN (b) as presented in Fig. 9, as well as EPIC-Italy (c), NOVI (d), and URECA (e). [file 13148_2022_1277_MOESM11_ESM.pdf]

Batch-effect susceptible probes

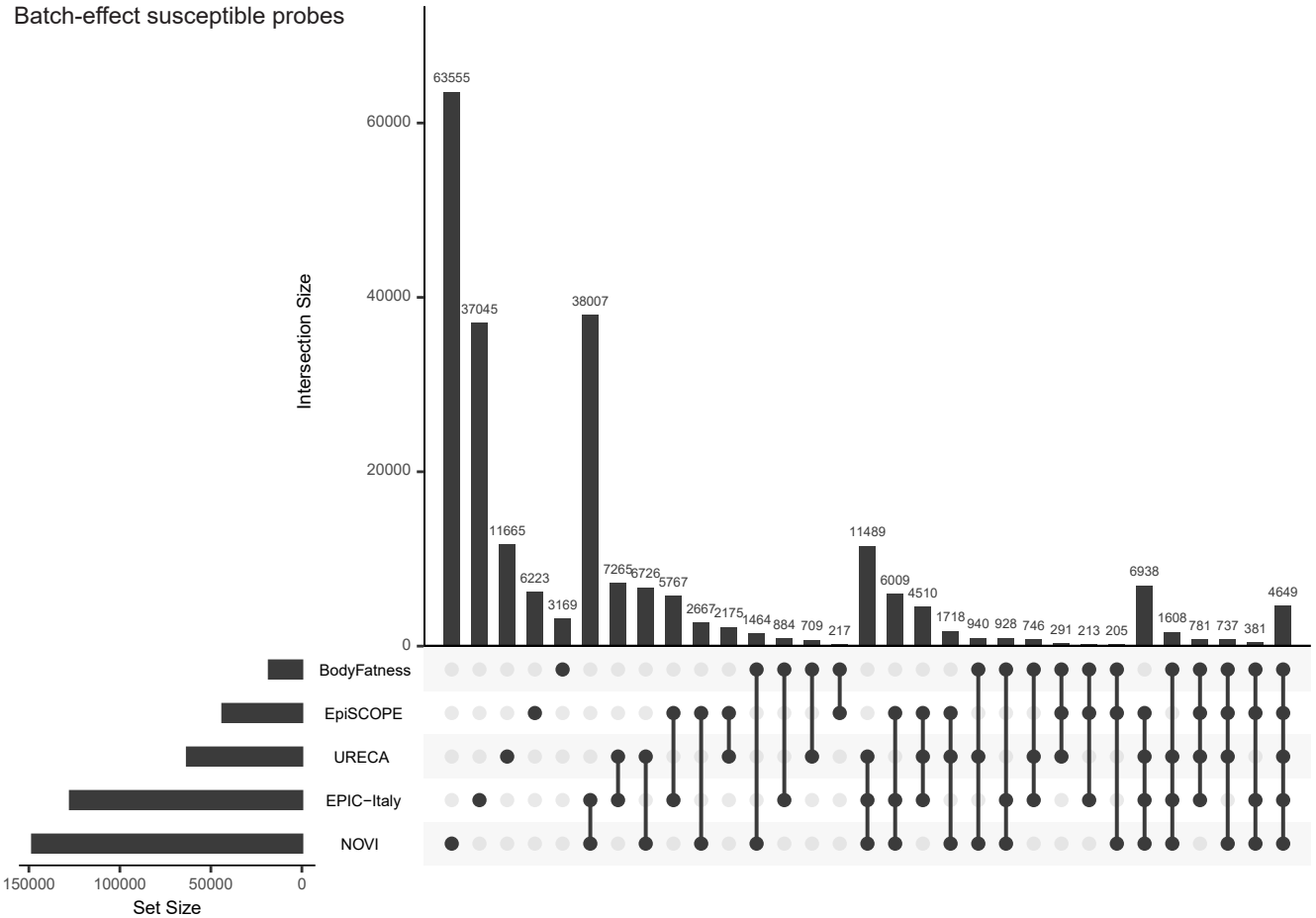

Supplement: Supplementary file 12 — Additional file 12: Figure S12. Intersection of batch-effect susceptible probes across the five datasets. The upset plot presents the number of batch-effect susceptible probes per dataset as well as their intersections. A total of 4649 probes were common to all datasets. [file 13148_2022_1277_MOESM12_ESM.pdf]

# Erroneously-corrected probes

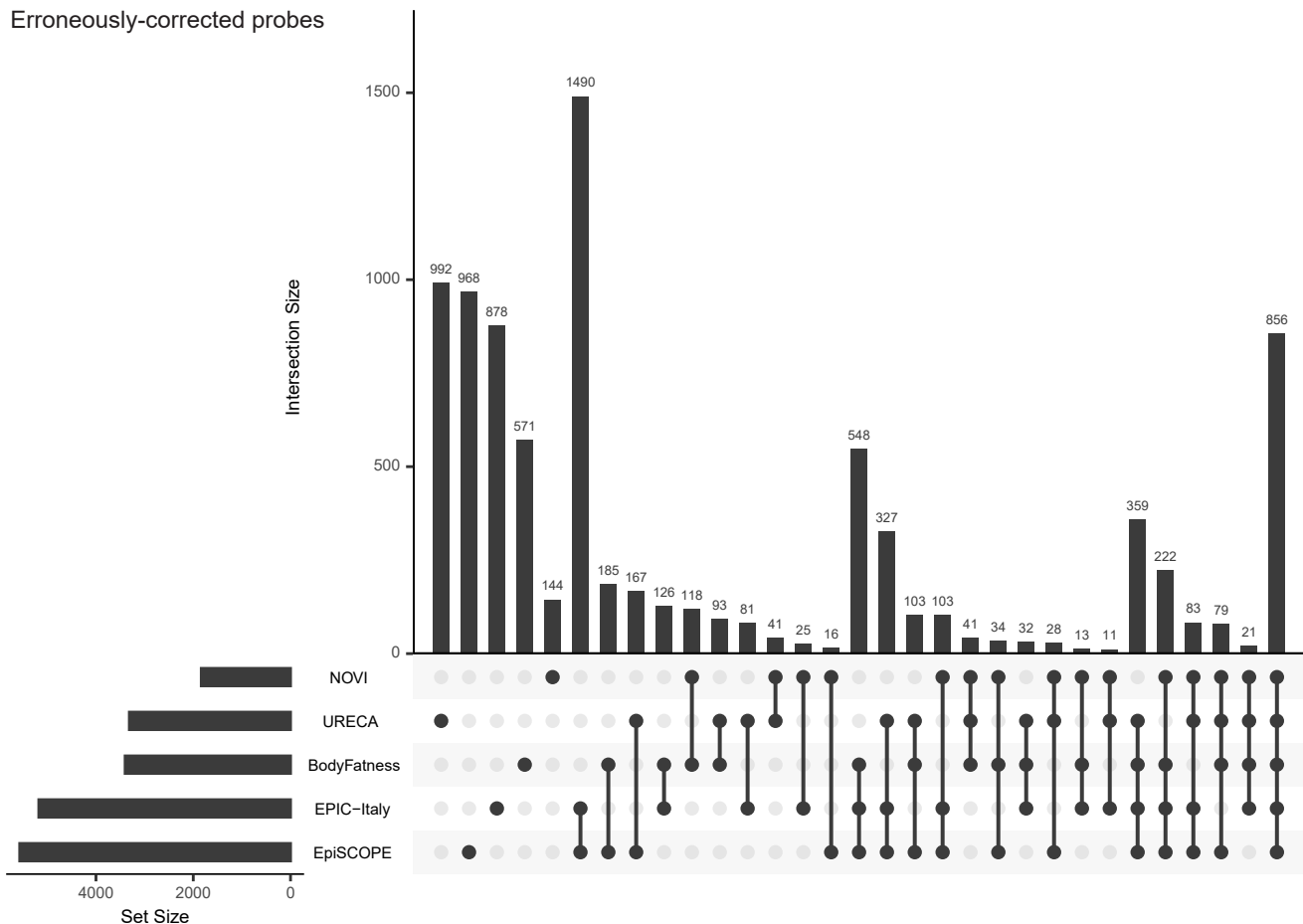

Supplement: Supplementary file 13 — Additional file 13: Figure S13. Intersection of erroneously corrected probes across the five datasets. The upset plot presents the number of erroneously corrected probes per dataset as well as their intersections. A total of 856 probes were common to all datasets. [file 13148_2022_1277_MOESM13_ESM.pdf]

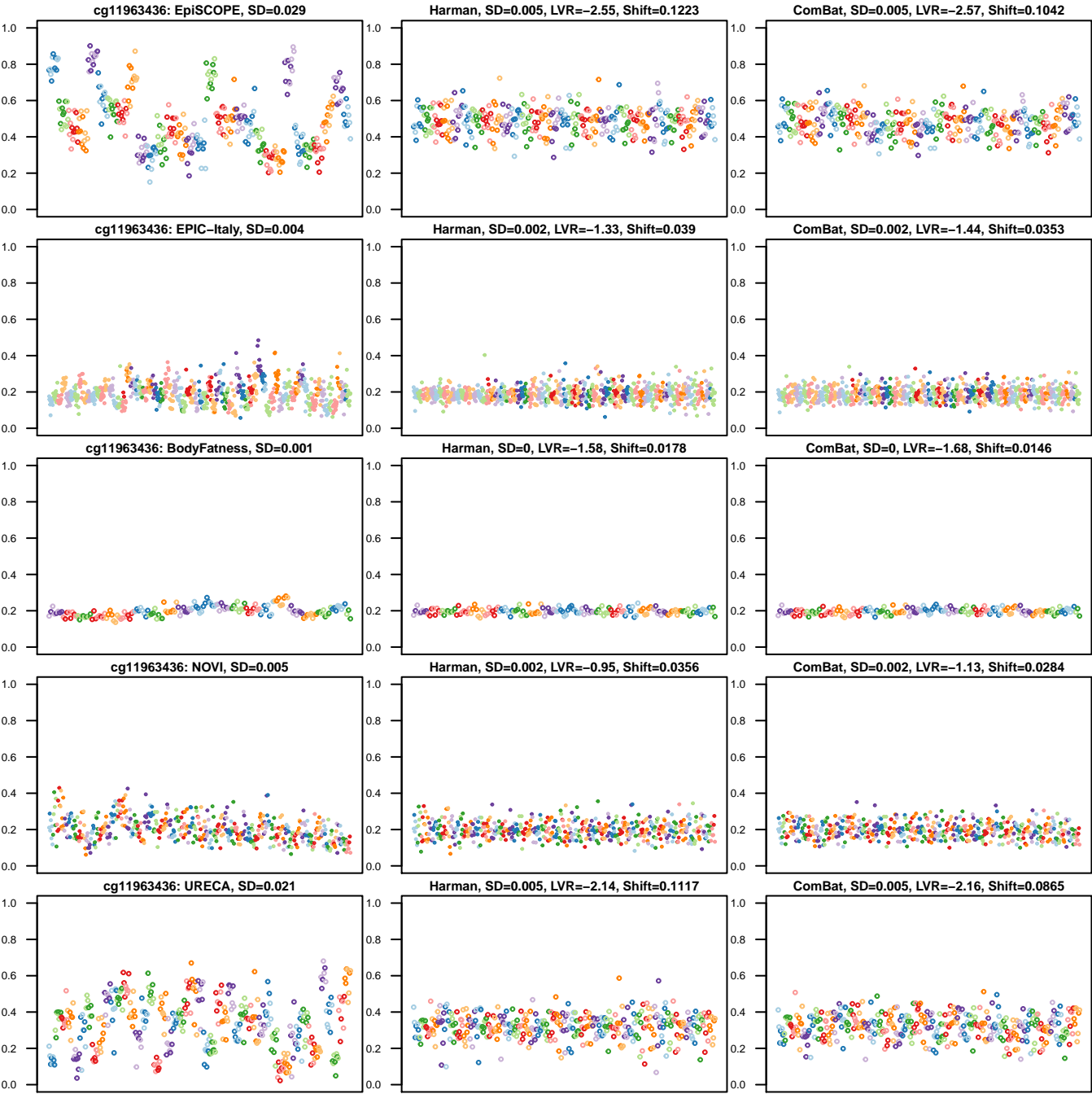

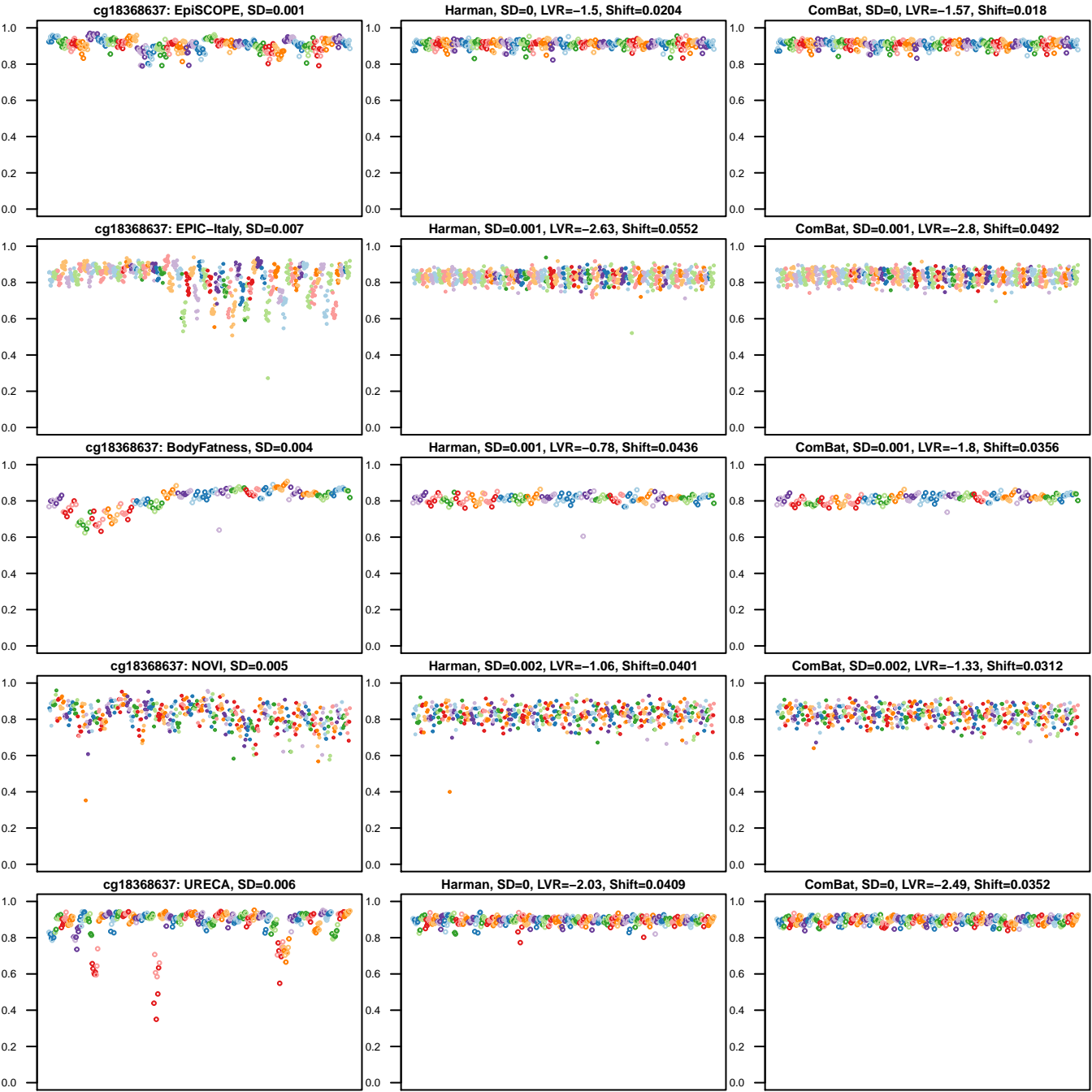

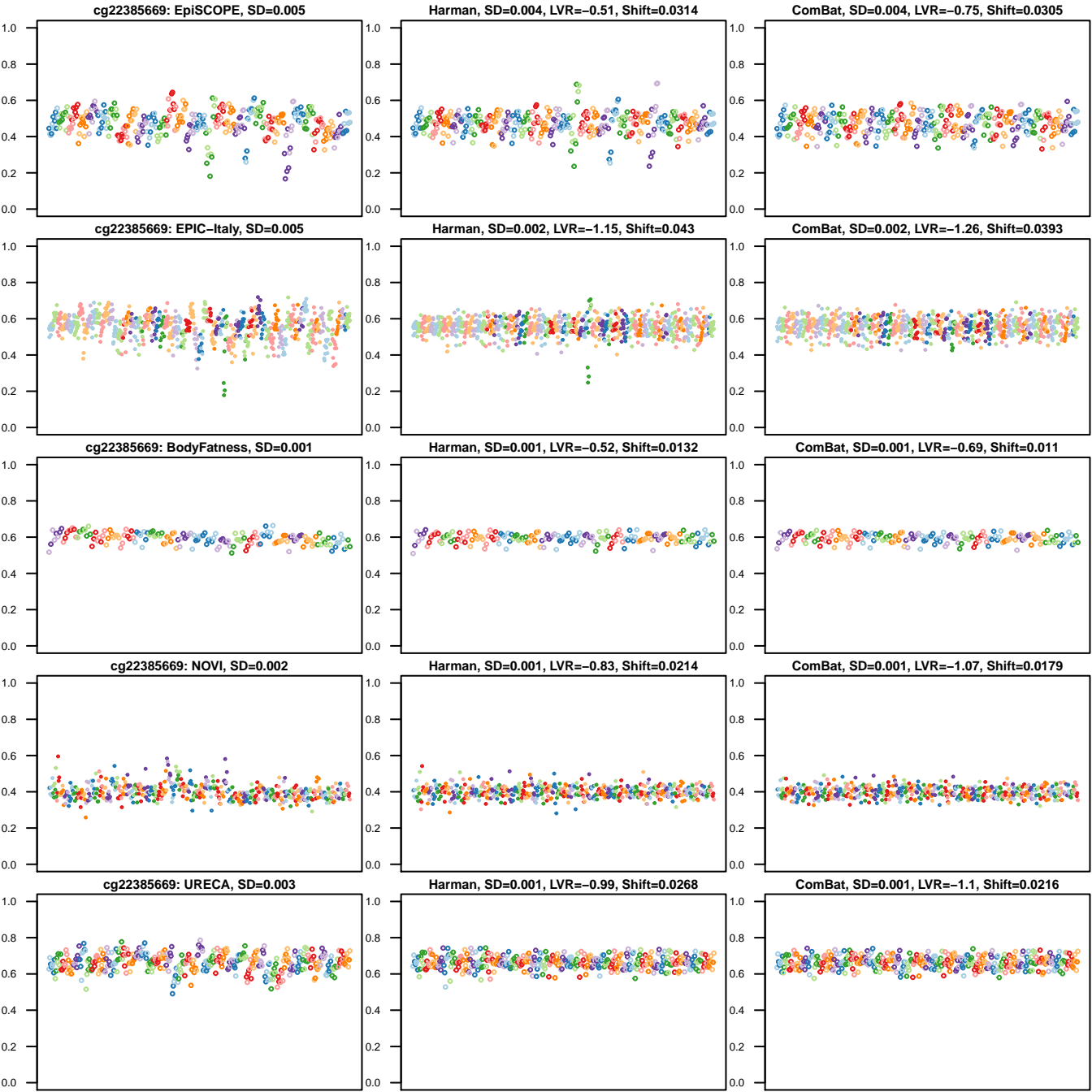

Supplement: Supplementary file 14 — Additional file 14: Figure S14. Three batch-effect susceptible probes reported as top EWAS findings. The three probes, (cg11963436, cg18368637, cg22385669), show obvious batch-effect in all the five datasets considered. Each scatter plot compares methylation across slides (X axis) with the methylation β value (Y-axis). The datapoints are the totality of the arrays from each study, sorted and coloured by slide number. The panel is ordered column-wise from left to right as original, Harman-corrected and ComBat-corrected data. In each instance, the standard deviation (SD) of the data was reduced, the log-variance ratio (LVR) was considerably below 0 and the mean β shift (Shift) was greater than 0.01. [file 13148_2022_1277_MOESM14_ESM.pdf]
