# Supplementary material for: Batch-effect detection, correction and characterisation in Illumina HumanMethylation450 and MethylationEPIC BeadChip array data
Source: Clin Epigenetics. 2022 Apr 29;14:58. doi: 10.1186/s13148-022-01277-9 (PMC9055778; doi:10.1186/s13148-022-01277-9)
Supplement: Supplementary file 3 — Additional file 3: Figure S3. Full set of control probes for the EpiSCOPE study. Further detail on the controls is provided by Illumina in the BeadArray Controls Reporter Software Guide document. [file 13148_2022_1277_MOESM3_ESM.pdf]

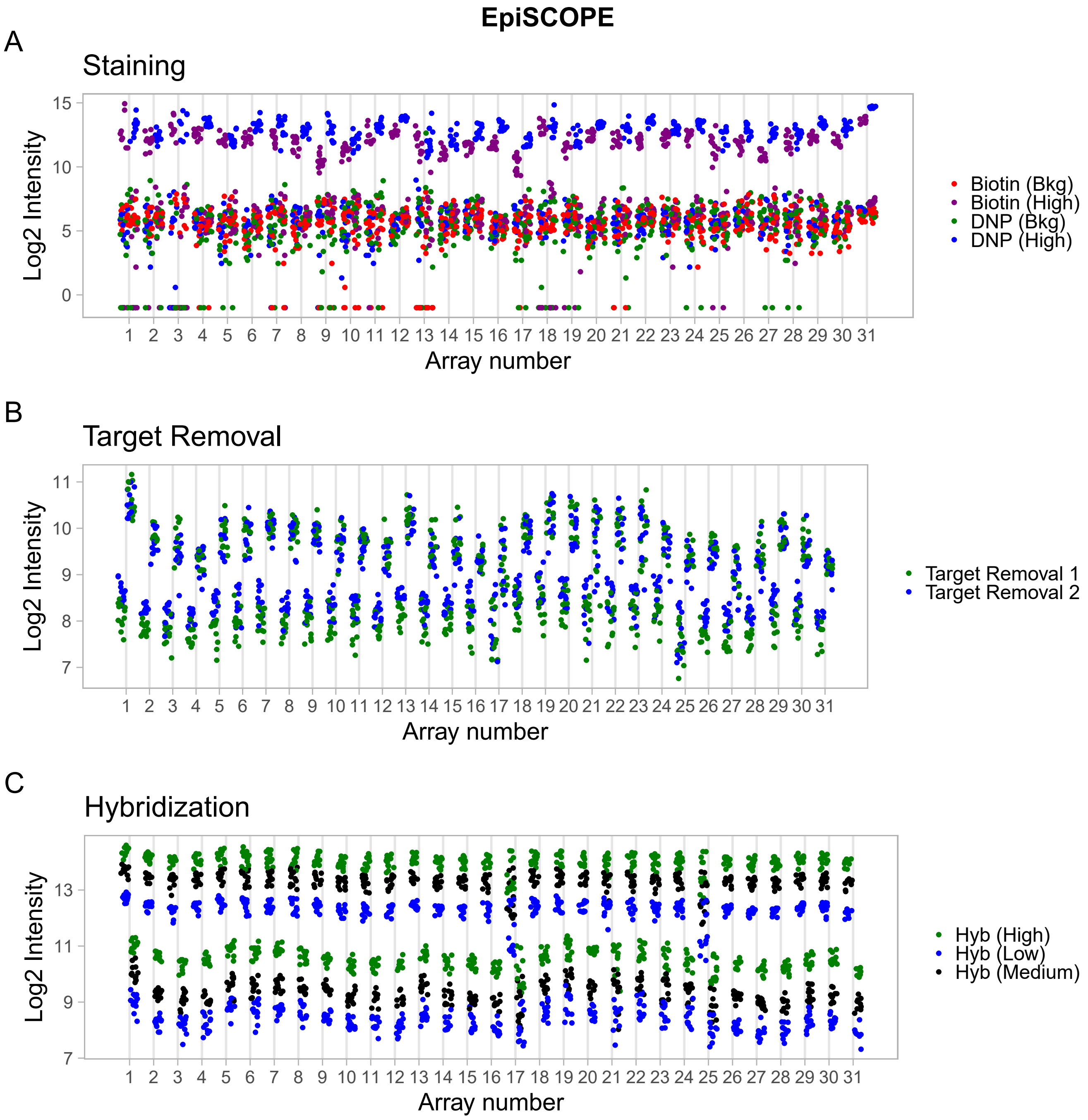
EpiSCOPE
A
Staining
Log2 Intensity
15
10
5
0
1 2 3 4 5 6 7 8 9 10 11 12 13 14 15 16 17 18 19 20 21 22 23 24 25 26 27 28 29 30 31
Array number
Biotin (Bkg)
Biotin (High)
DNP (Bkg)
DNP (High)
B
Target Removal
Log2 Intensity
11
10
9
8
7
1 2 3 4 5 6 7 8 9 10 11 12 13 14 15 16 17 18 19 20 21 22 23 24 25 26 27 28 29 30 31
Array number
Target Removal 1
Target Removal 2
C
Hybridization
Log2 Intensity
13
11
9
7
1 2 3 4 5 6 7 8 9 10 11 12 13 14 15 16 17 18 19 20 21 22 23 24 25 26 27 28 29 30 31
Array number
Hyb (High)
Hyb (Low)
Hyb (Medium)

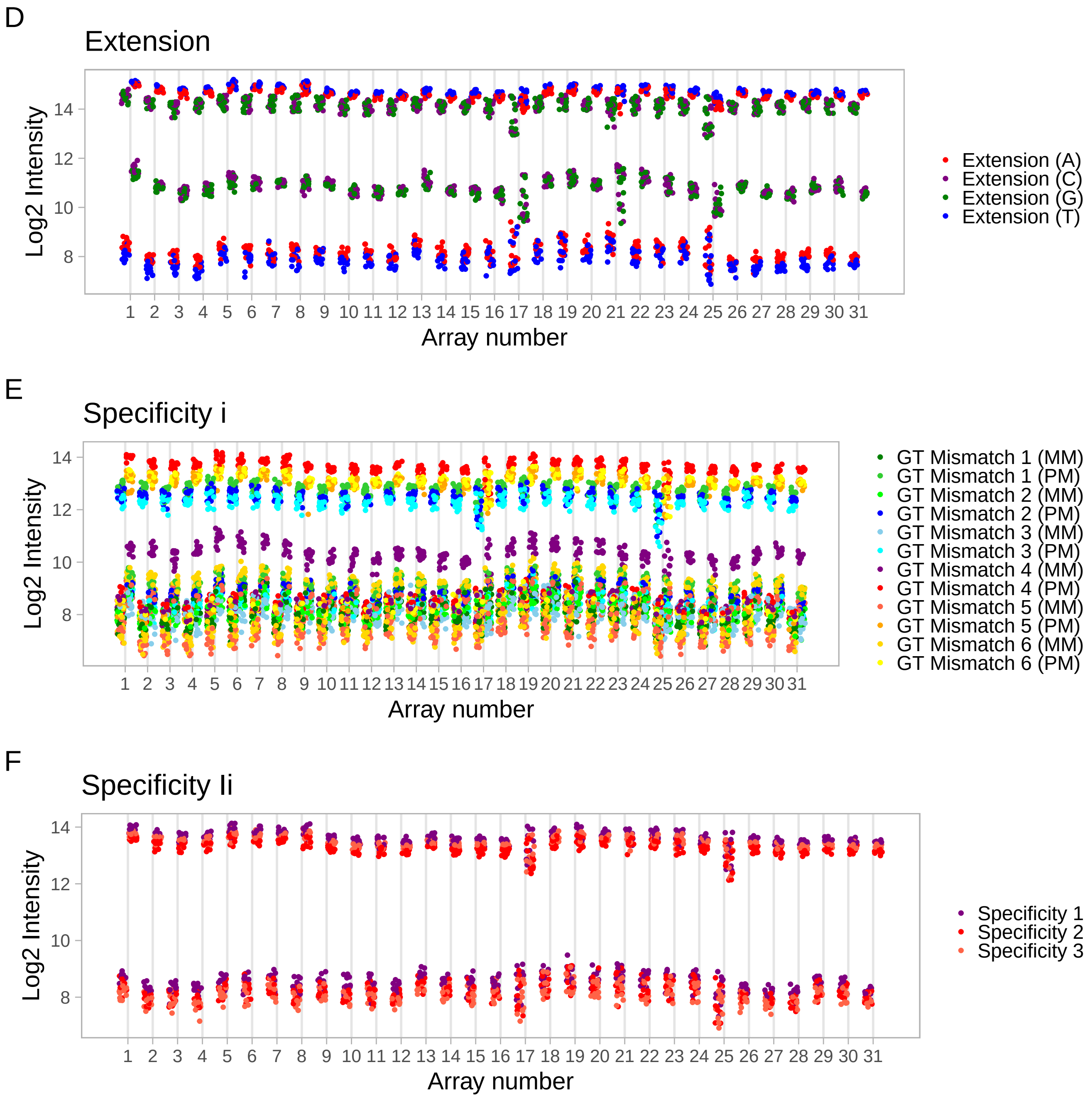

D
Extension
Log2 Intensity
8
10
12
14
1 2 3 4 5 6 7 8 9 10 11 12 13 14 15 16 17 18 19 20 21 22 23 24 25 26 27 28 29 30 31
Array number
Extension (A)
Extension (C)
Extension (G)
Extension (T)
E
Specificity i
Log2 Intensity
8
10
12
14
1 2 3 4 5 6 7 8 9 10 11 12 13 14 15 16 17 18 19 20 21 22 23 24 25 26 27 28 29 30 31
Array number
GT Mismatch 1 (MM)
GT Mismatch 1 (PM)
GT Mismatch 2 (MM)
GT Mismatch 2 (PM)
GT Mismatch 3 (MM)
GT Mismatch 3 (PM)
GT Mismatch 4 (MM)
GT Mismatch 4 (PM)
GT Mismatch 5 (MM)
GT Mismatch 5 (PM)
GT Mismatch 6 (MM)
GT Mismatch 6 (PM)
F
Specificity Ii
Log2 Intensity
8
10
12
14
1 2 3 4 5 6 7 8 9 10 11 12 13 14 15 16 17 18 19 20 21 22 23 24 25 26 27 28 29 30 31
Array number
Specificity 1
Specificity 2
Specificity 3

G

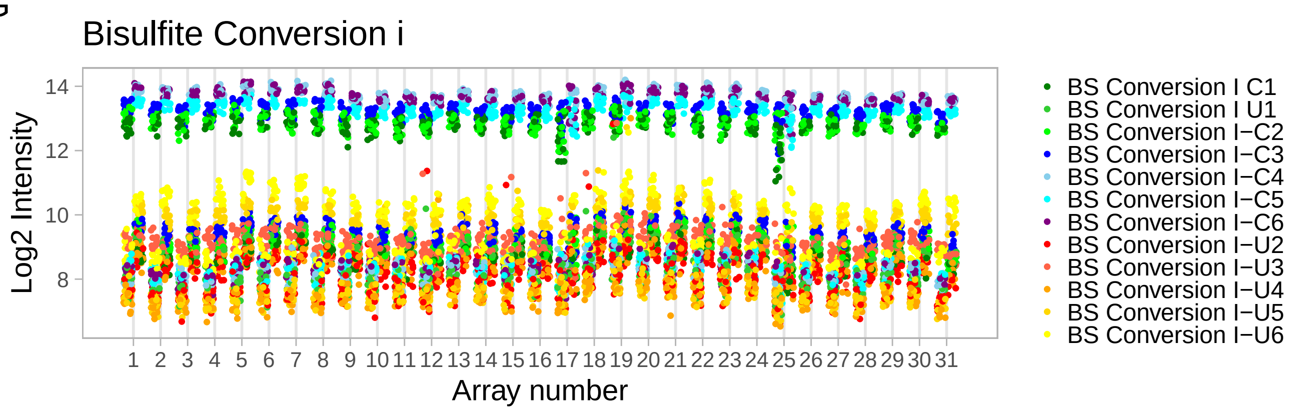
Bisulfite Conversion i
Log2 Intensity
8
10
12
14
1 2 3 4 5 6 7 8 9 10 11 12 13 14 15 16 17 18 19 20 21 22 23 24 25 26 27 28 29 30 31
Array number
BS Conversion I C1
BS Conversion I U1
BS Conversion I−C2
BS Conversion I−C3
BS Conversion I−C4
BS Conversion I−C5
BS Conversion I−C6
BS Conversion I−U2
BS Conversion I−U3
BS Conversion I−U4
BS Conversion I−U5
BS Conversion I−U6

H

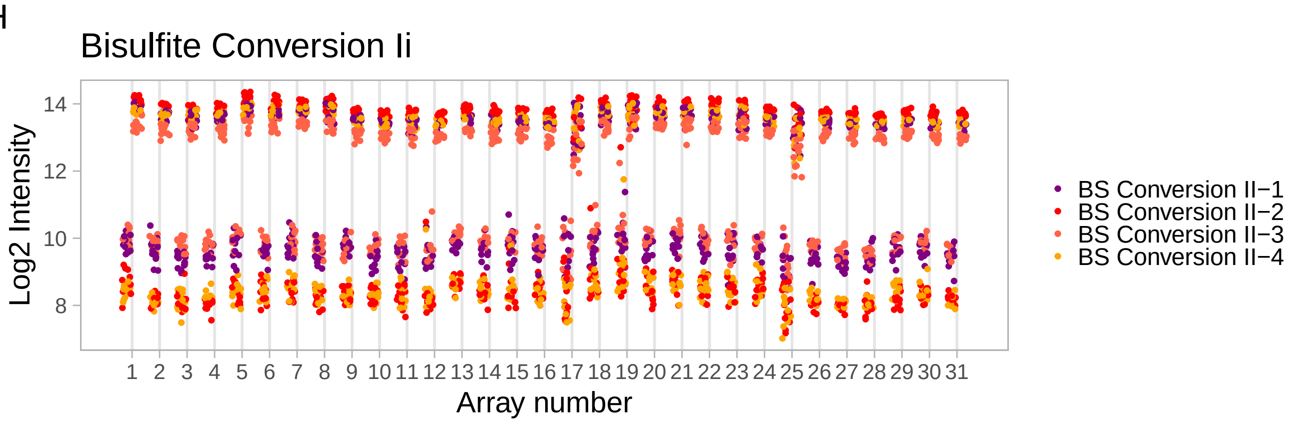
Bisulfite Conversion Ii
Log2 Intensity
8
10
12
14
1 2 3 4 5 6 7 8 9 10 11 12 13 14 15 16 17 18 19 20 21 22 23 24 25 26 27 28 29 30 31
Array number
BS Conversion II−1
BS Conversion II−2
BS Conversion II−3
BS Conversion II−4

I

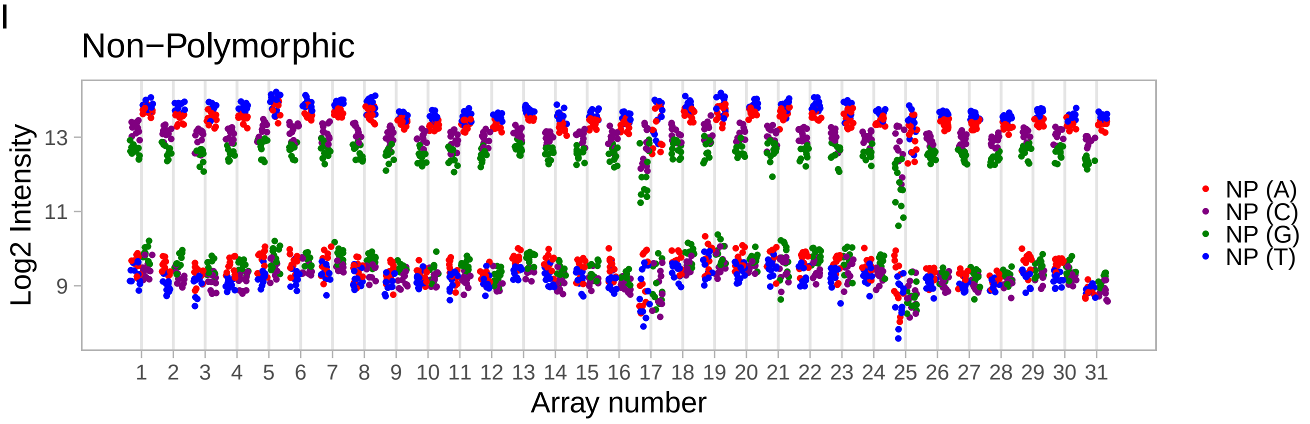
Non−Polymorphic
Log2 Intensity
9
11
13
1 2 3 4 5 6 7 8 9 10 11 12 13 14 15 16 17 18 19 20 21 22 23 24 25 26 27 28 29 30 31
Array number
NP (A)
NP (C)
NP (G)
NP (T)

J

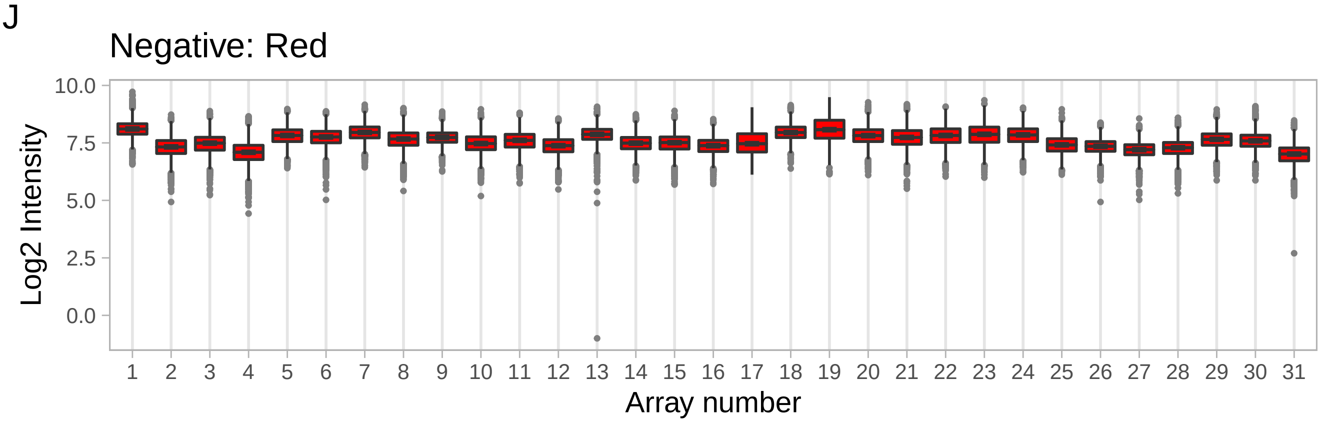
Negative: Red
Log2 Intensity
10.0
7.5
5.0
2.5
0.0
1 2 3 4 5 6 7 8 9 10 11 12 13 14 15 16 17 18 19 20 21 22 23 24 25 26 27 28 29 30 31
Array number

K

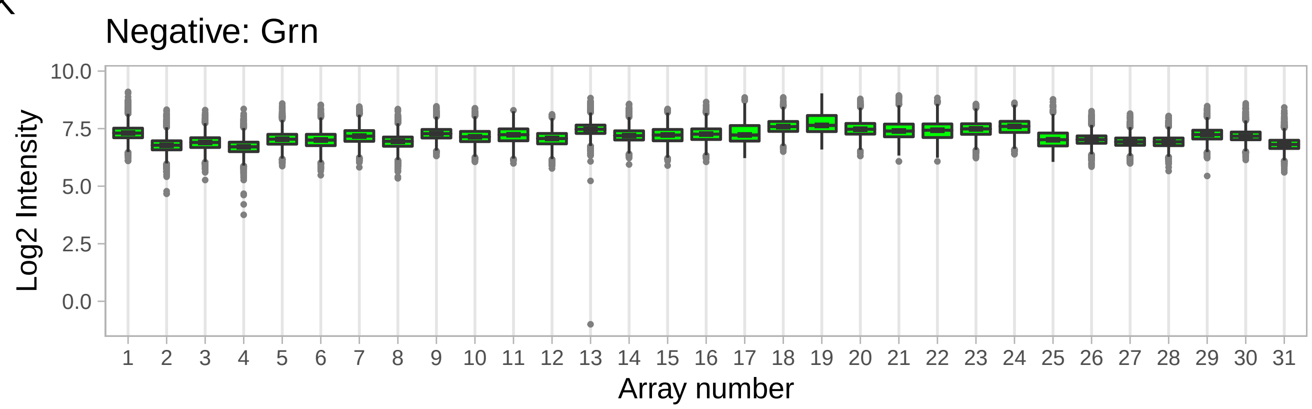
Negative: Grn
Log2 Intensity
10.0
7.5
5.0
2.5
0.0
1 2 3 4 5 6 7 8 9 10 11 12 13 14 15 16 17 18 19 20 21 22 23 24 25 26 27 28 29 30 31
Array number
