# Supplementary material for: Batch-effect detection, correction and characterisation in Illumina HumanMethylation450 and MethylationEPIC BeadChip array data
Source: Clin Epigenetics. 2022 Apr 29;14:58. doi: 10.1186/s13148-022-01277-9 (PMC9055778; doi:10.1186/s13148-022-01277-9)
Supplement: Supplementary file 4 — Additional file 4: Figure S4. Full set of control probes for the BFiN study. Further detail on the controls is provided by Illumina in the BeadArray Controls Reporter Software Guide document. [file 13148_2022_1277_MOESM4_ESM.pdf]

BFiN

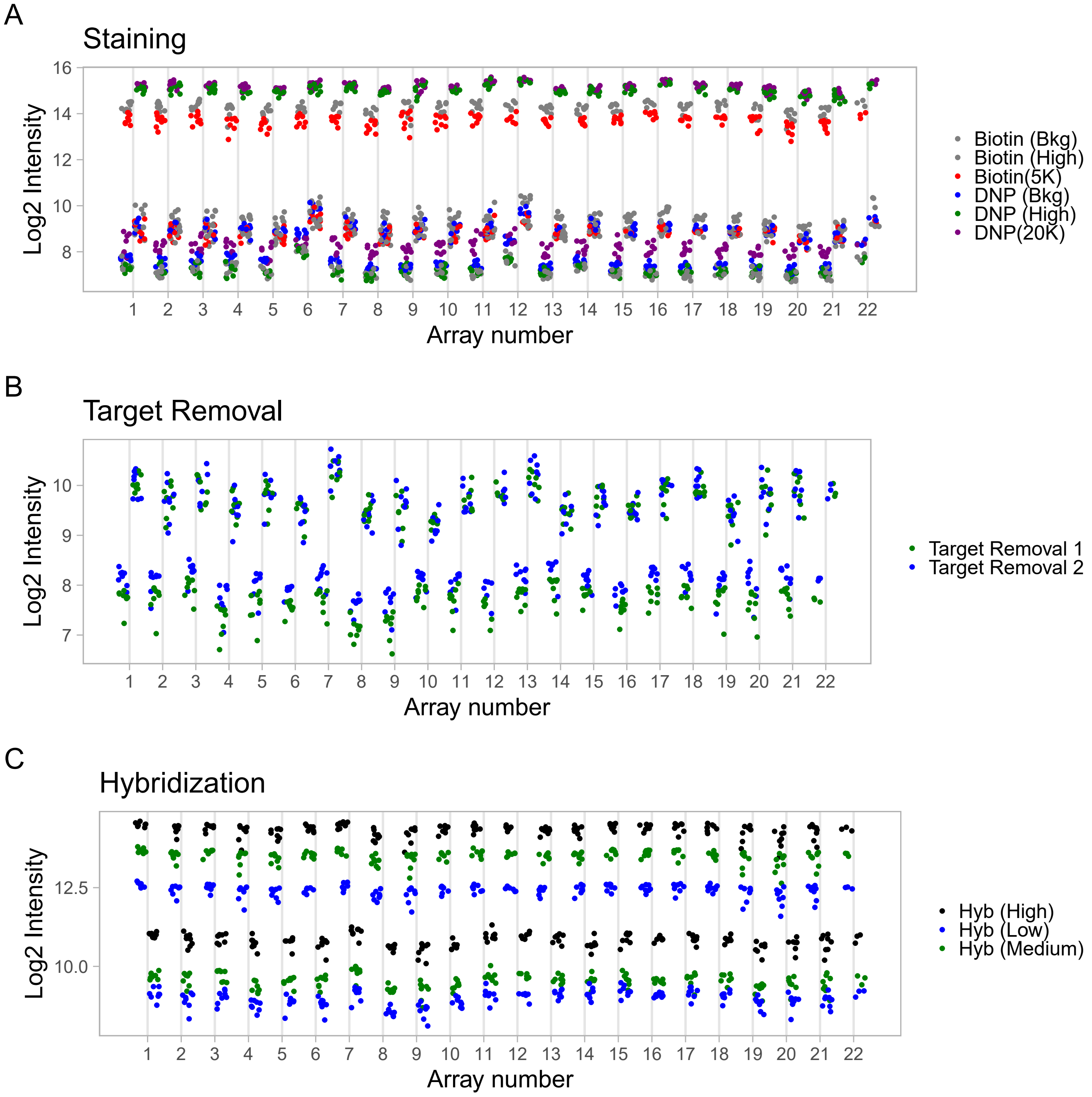
A
Staining
Log2 Intensity
16
14
12
10
8
Array number
Biotin (Bkg)
Biotin (High)
Biotin(5K)
DNP (Bkg)
DNP (High)
DNP(20K)
B
Target Removal
Log2 Intensity
10
9
8
7
Array number
Target Removal 1
Target Removal 2
C
Hybridization
Log2 Intensity
12.5
10.0
Array number
Hyb (High)
Hyb (Low)
Hyb (Medium)
1 2 3 4 5 6 7 8 9 10 11 12 13 14 15 16 17 18 19 20 21 22

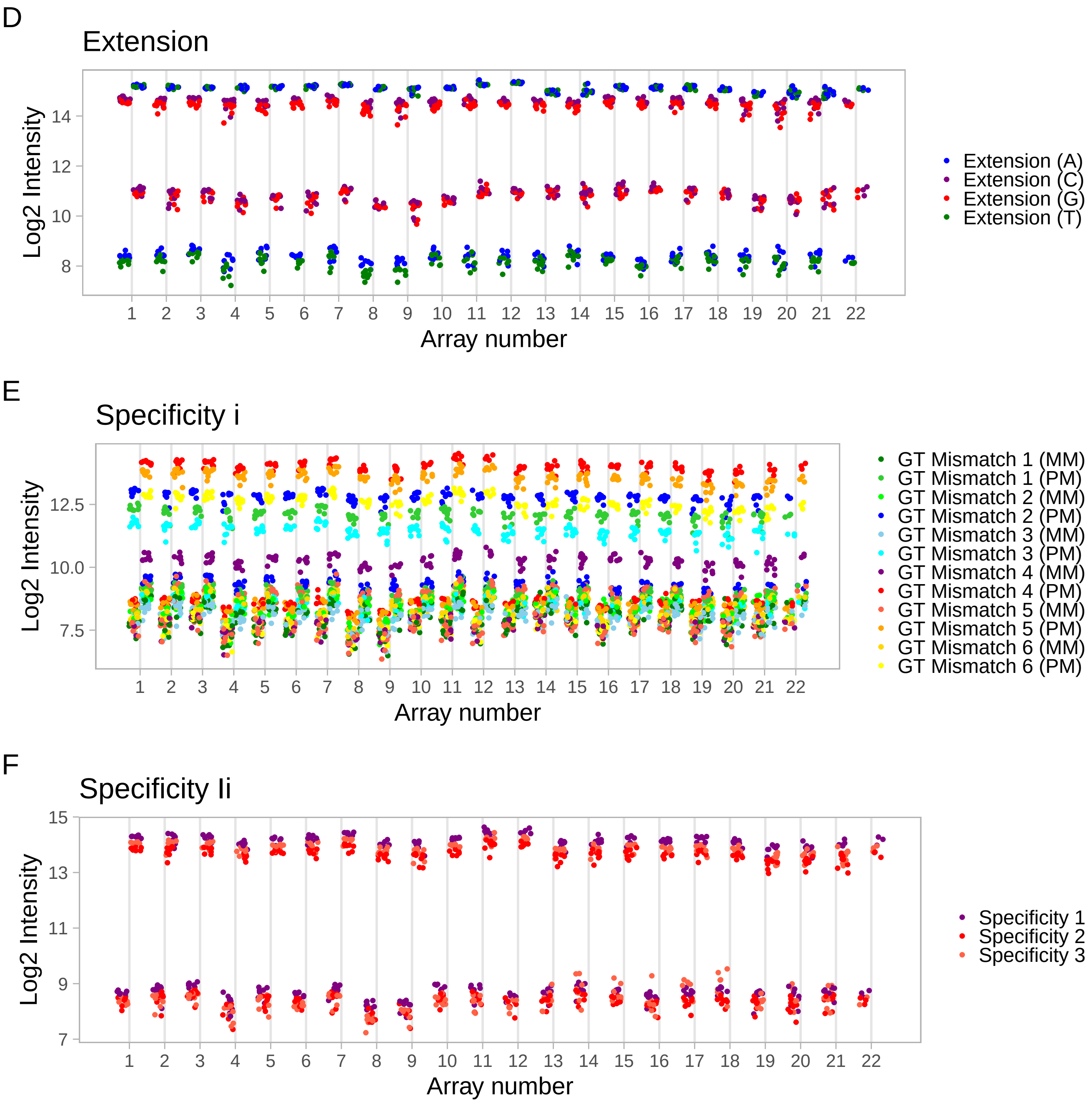

D
Extension
Log2 Intensity
8
10
12
14
1 2 3 4 5 6 7 8 9 10 11 12 13 14 15 16 17 18 19 20 21 22
Array number
Extension (A)
Extension (C)
Extension (G)
Extension (T)
E
Specificity i
Log2 Intensity
7.5
10.0
12.5
1 2 3 4 5 6 7 8 9 10 11 12 13 14 15 16 17 18 19 20 21 22
Array number
GT Mismatch 1 (MM)
GT Mismatch 1 (PM)
GT Mismatch 2 (MM)
GT Mismatch 2 (PM)
GT Mismatch 3 (MM)
GT Mismatch 3 (PM)
GT Mismatch 4 (MM)
GT Mismatch 4 (PM)
GT Mismatch 5 (MM)
GT Mismatch 5 (PM)
GT Mismatch 6 (MM)
GT Mismatch 6 (PM)
F
Specificity Ii
Log2 Intensity
7
9
11
13
15
1 2 3 4 5 6 7 8 9 10 11 12 13 14 15 16 17 18 19 20 21 22
Array number
Specificity 1
Specificity 2
Specificity 3

G

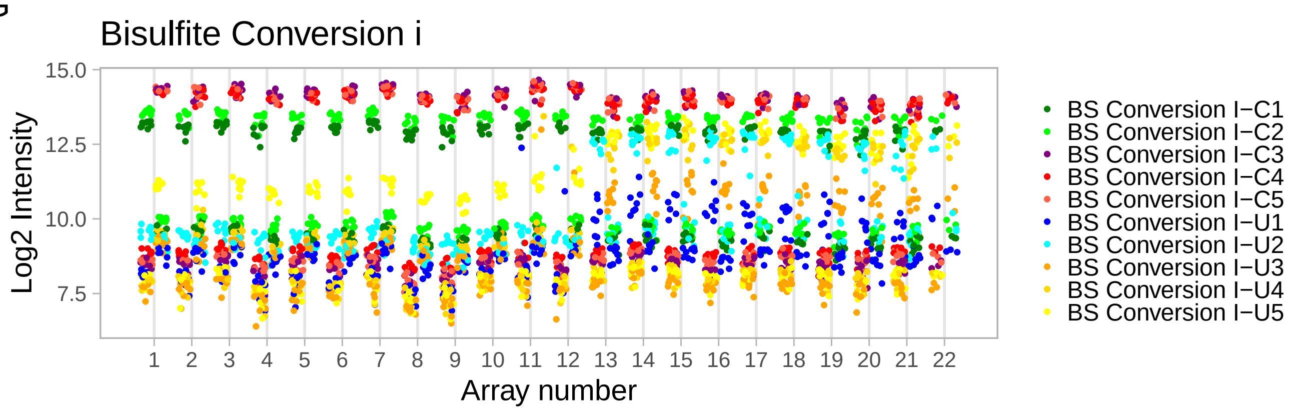

Bisulfite Conversion i
Log2 Intensity
15.0
12.5
10.0
7.5
1 2 3 4 5 6 7 8 9 10 11 12 13 14 15 16 17 18 19 20 21 22
Array number
BS Conversion I−C1
BS Conversion I−C2
BS Conversion I−C3
BS Conversion I−C4
BS Conversion I−C5
BS Conversion I−U1
BS Conversion I−U2
BS Conversion I−U3
BS Conversion I−U4
BS Conversion I−U5

H

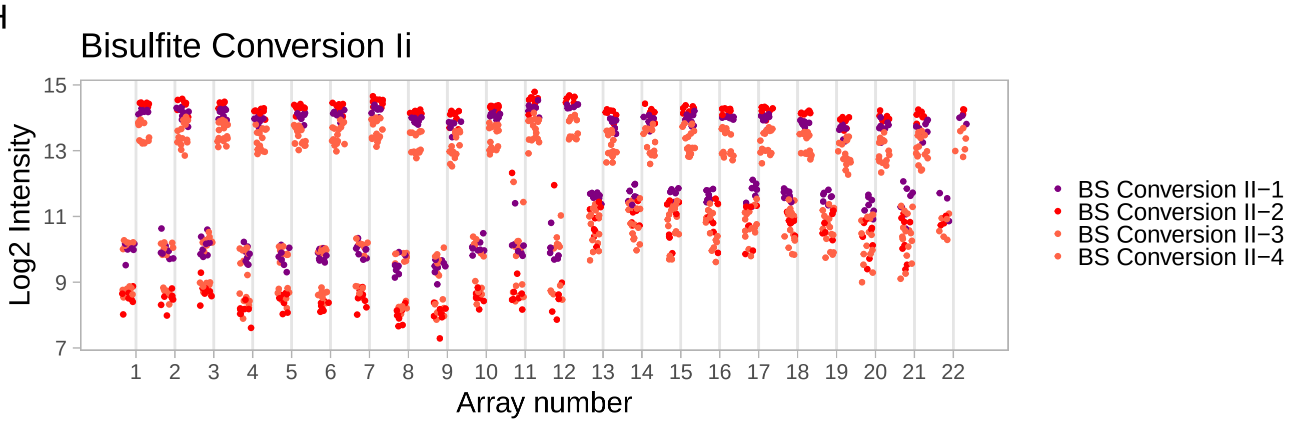

Bisulfite Conversion Ii
Log2 Intensity
15
13
11
9
7
1 2 3 4 5 6 7 8 9 10 11 12 13 14 15 16 17 18 19 20 21 22
Array number
BS Conversion II−1
BS Conversion II−2
BS Conversion II−3
BS Conversion II−4

I

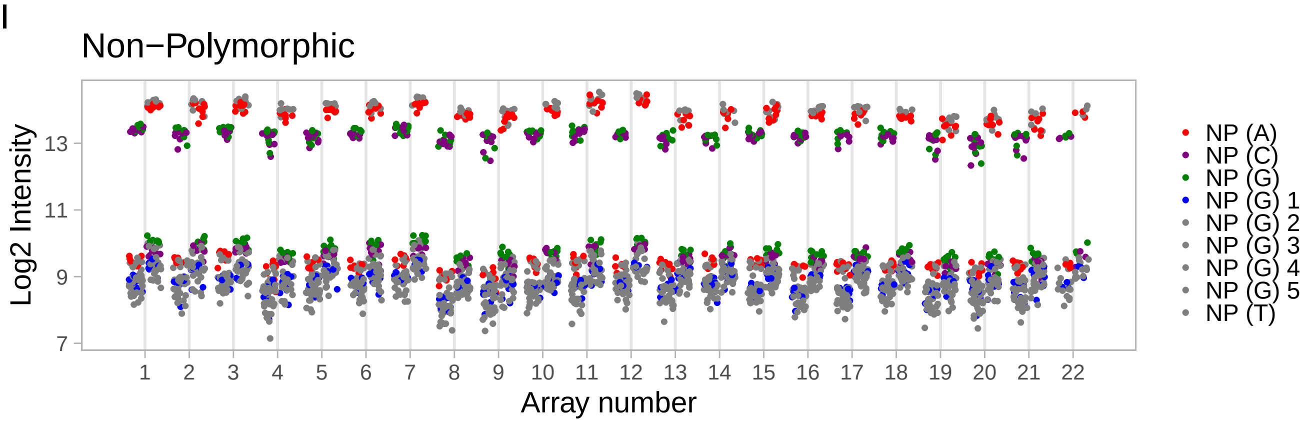

Non−Polymorphic
Log2 Intensity
13
11
9
7
1 2 3 4 5 6 7 8 9 10 11 12 13 14 15 16 17 18 19 20 21 22
Array number
NP (A)
NP (C)
NP (G)
NP (G) 1
NP (G) 2
NP (G) 3
NP (G) 4
NP (G) 5
NP (T)

J

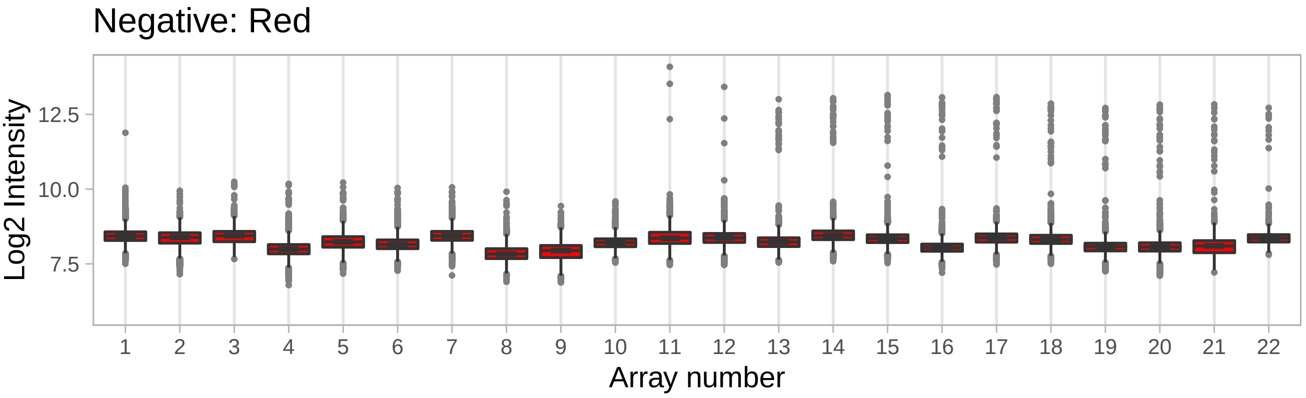
Negative: Red
Log2 Intensity
12.5
10.0
7.5
1 2 3 4 5 6 7 8 9 10 11 12 13 14 15 16 17 18 19 20 21 22
Array number

K

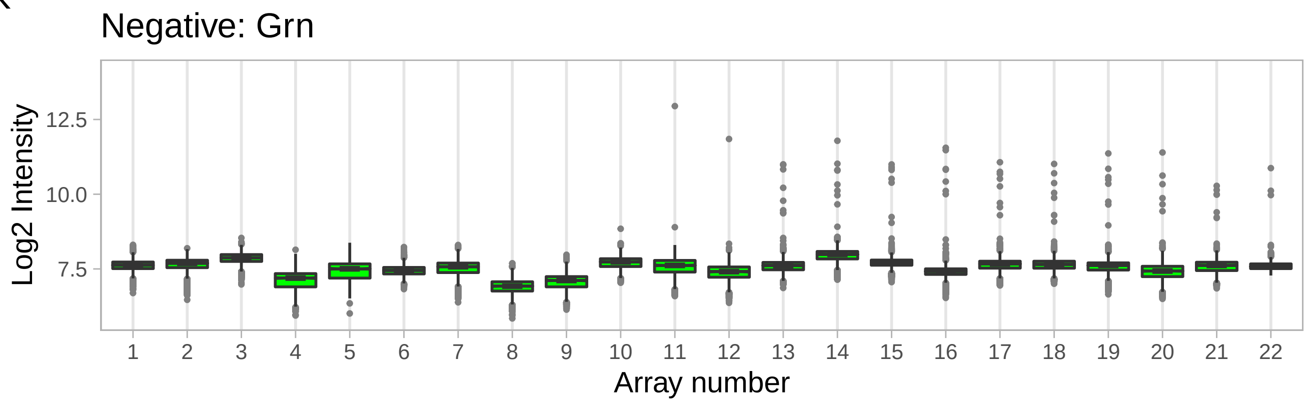
Negative: Grn
Log2 Intensity
12.5
10.0
7.5
1 2 3 4 5 6 7 8 9 10 11 12 13 14 15 16 17 18 19 20 21 22
Array number
