# Supplementary material for: Batch-effect detection, correction and characterisation in Illumina HumanMethylation450 and MethylationEPIC BeadChip array data
Source: Clin Epigenetics. 2022 Apr 29;14:58. doi: 10.1186/s13148-022-01277-9 (PMC9055778; doi:10.1186/s13148-022-01277-9)
Supplement: Supplementary file 7 — Additional file 7: Figure S7. BFiN modal probe associations The upset plot for BFiN data. Refer to Additional file 6: Fig. S6 for further description of the factors. [file 13148_2022_1277_MOESM7_ESM.pdf]

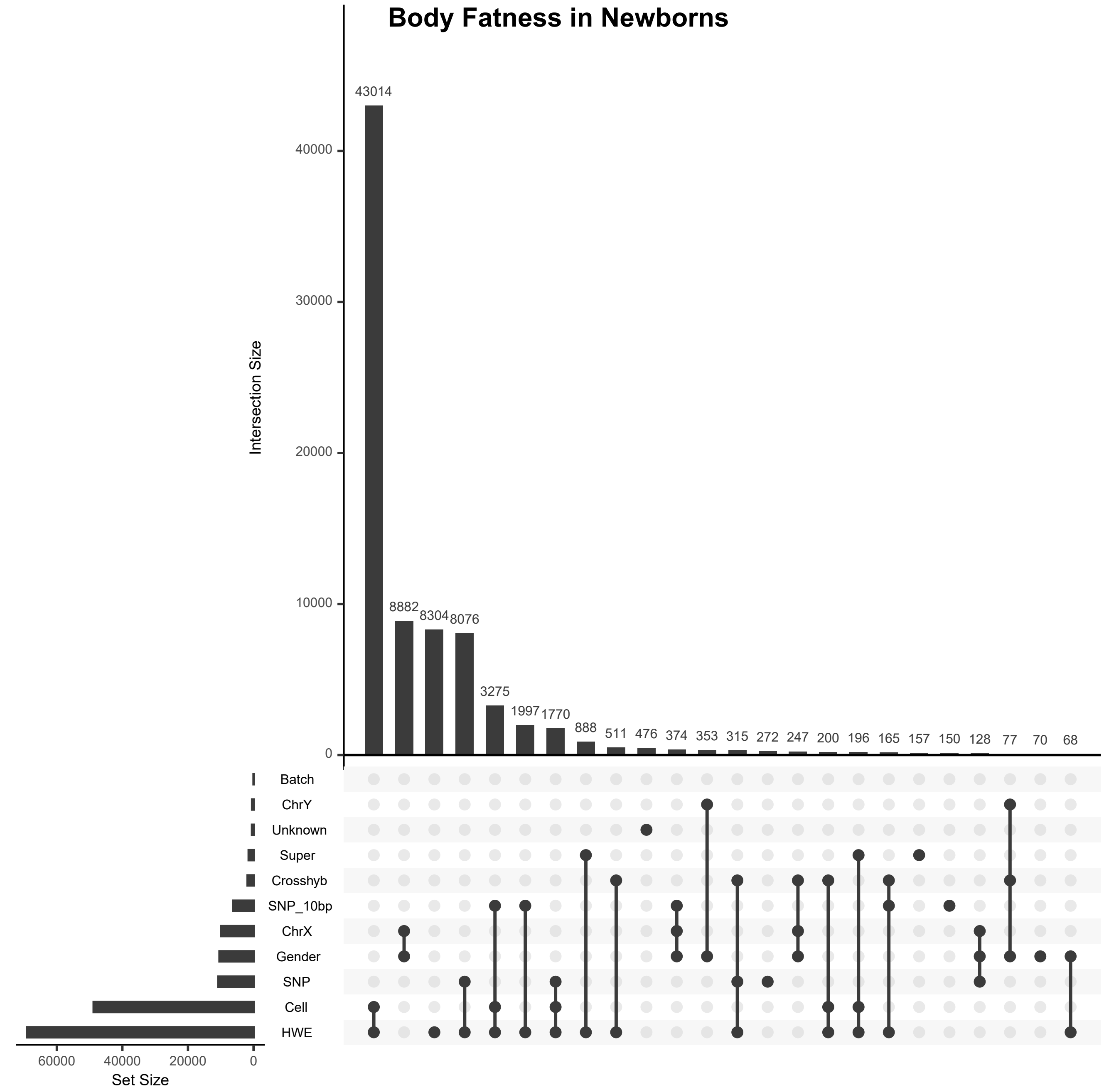

Body Fatness in Newborns
Intersection Size
0
10000
20000
30000
40000
43014
8882
8304
8076
3275
1997
1770
888
511
476
374
353
315
272
247
200
196
165
157
150
128
77
70
68
Batch
ChrY
Unknown
Super
Crosshyb
SNP_10bp
ChrX
Gender
SNP
Cell
HWE
60000
40000
20000
0
Set Size
