# Supplementary material for: Batch-effect detection, correction and characterisation in Illumina HumanMethylation450 and MethylationEPIC BeadChip array data
Source: Clin Epigenetics. 2022 Apr 29;14:58. doi: 10.1186/s13148-022-01277-9 (PMC9055778; doi:10.1186/s13148-022-01277-9)
Supplement: Supplementary file 8 — Additional file 8: Figure S8. Full set of control probes for the EPIC-Italy study. Further detail on the controls is provided by Illumina in the BeadArray Controls Reporter Software Guide document. [file 13148_2022_1277_MOESM8_ESM.pdf]

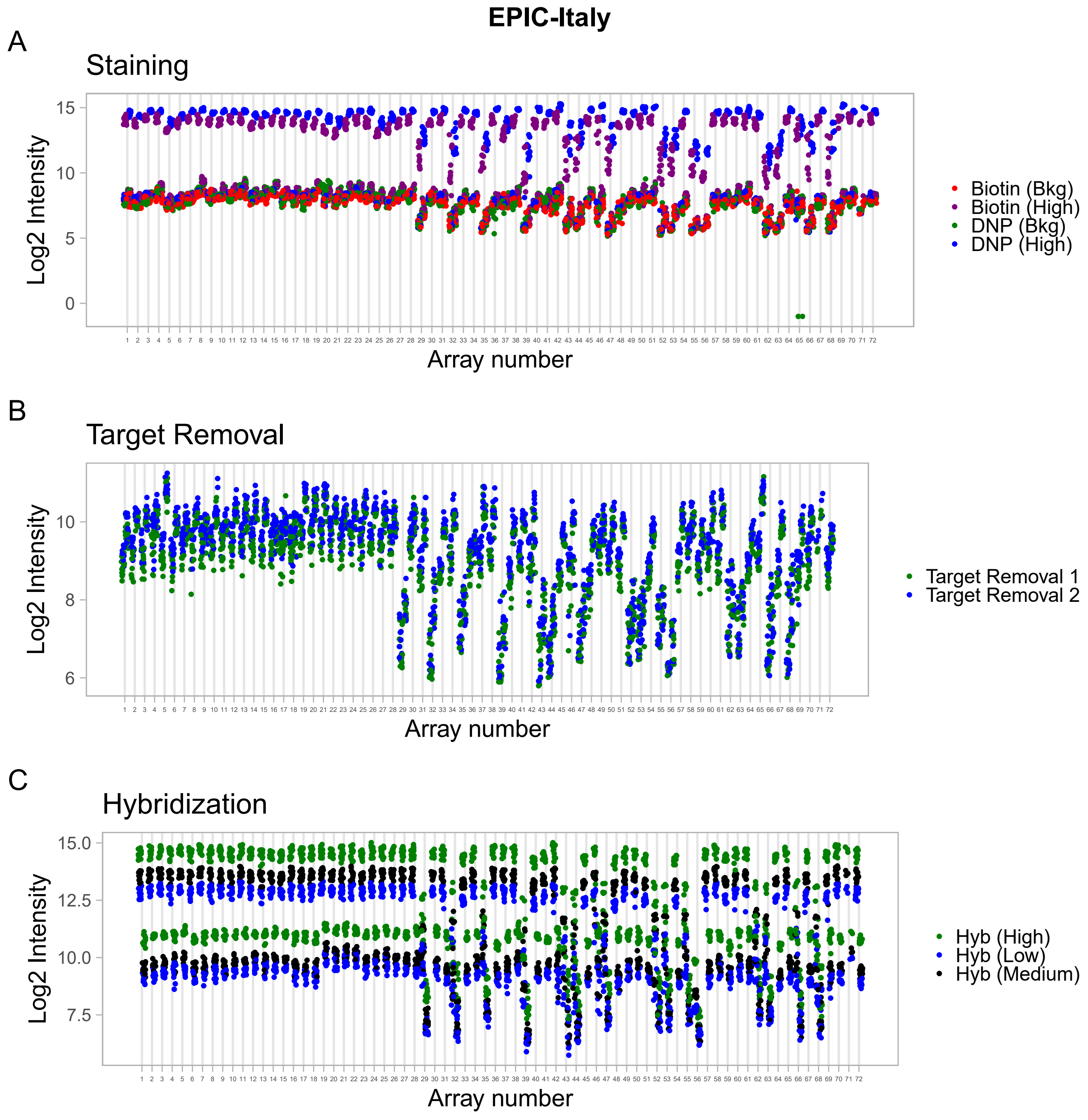
EPIC-Italy
A
Staining
Log2 Intensity
15
10
5
0
Array number
Biotin (Bkg)
Biotin (High)
DNP (Bkg)
DNP (High)
B
Target Removal
Log2 Intensity
10
8
6
Array number
Target Removal 1
Target Removal 2
C
Hybridization
Log2 Intensity
15.0
12.5
10.0
7.5
Array number
Hyb (High)
Hyb (Low)
Hyb (Medium)

D

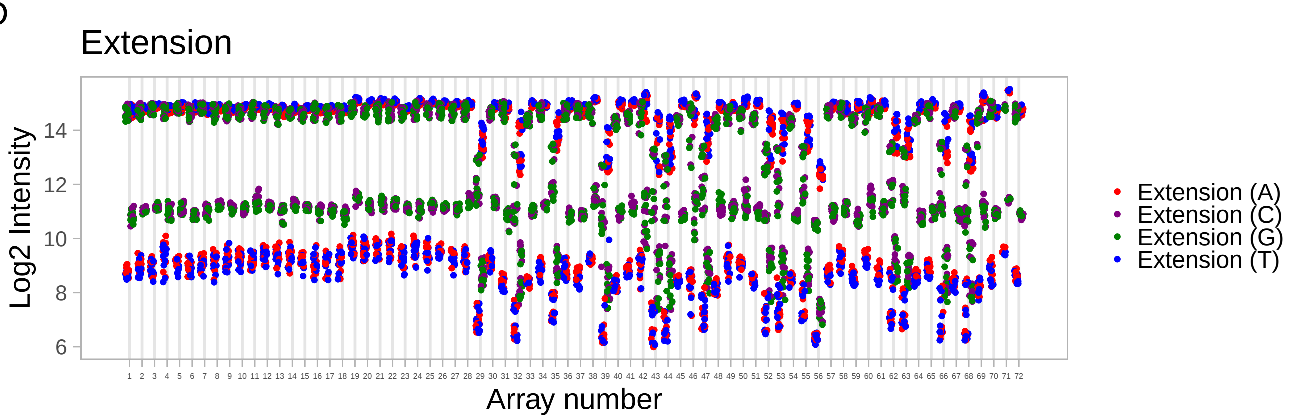
Extension
Log2 Intensity
6
8
10
12
14
Array number
Extension (A)
Extension (C)
Extension (G)
Extension (T)

E

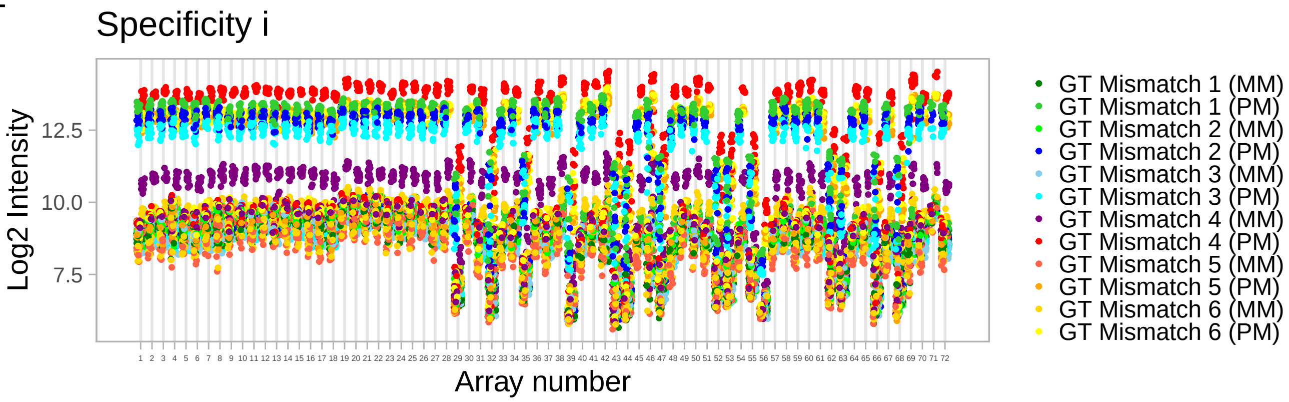
Specificity i
Log2 Intensity
7.5
10.0
12.5
Array number
GT Mismatch 1 (MM)
GT Mismatch 1 (PM)
GT Mismatch 2 (MM)
GT Mismatch 2 (PM)
GT Mismatch 3 (MM)
GT Mismatch 3 (PM)
GT Mismatch 4 (MM)
GT Mismatch 4 (PM)
GT Mismatch 5 (MM)
GT Mismatch 5 (PM)
GT Mismatch 6 (MM)
GT Mismatch 6 (PM)

F

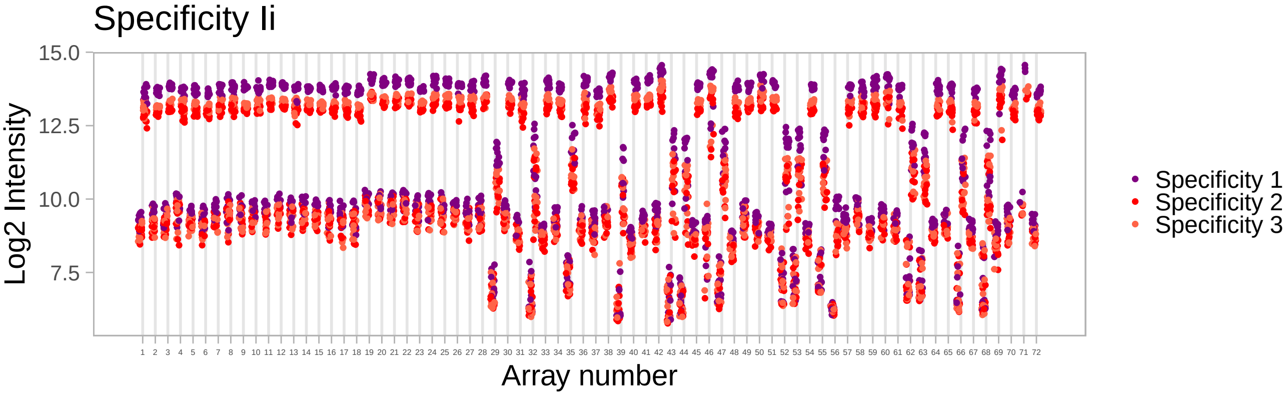
Specificity Ii
Log2 Intensity
7.5
10.0
12.5
15.0
Array number
Specificity 1
Specificity 2
Specificity 3

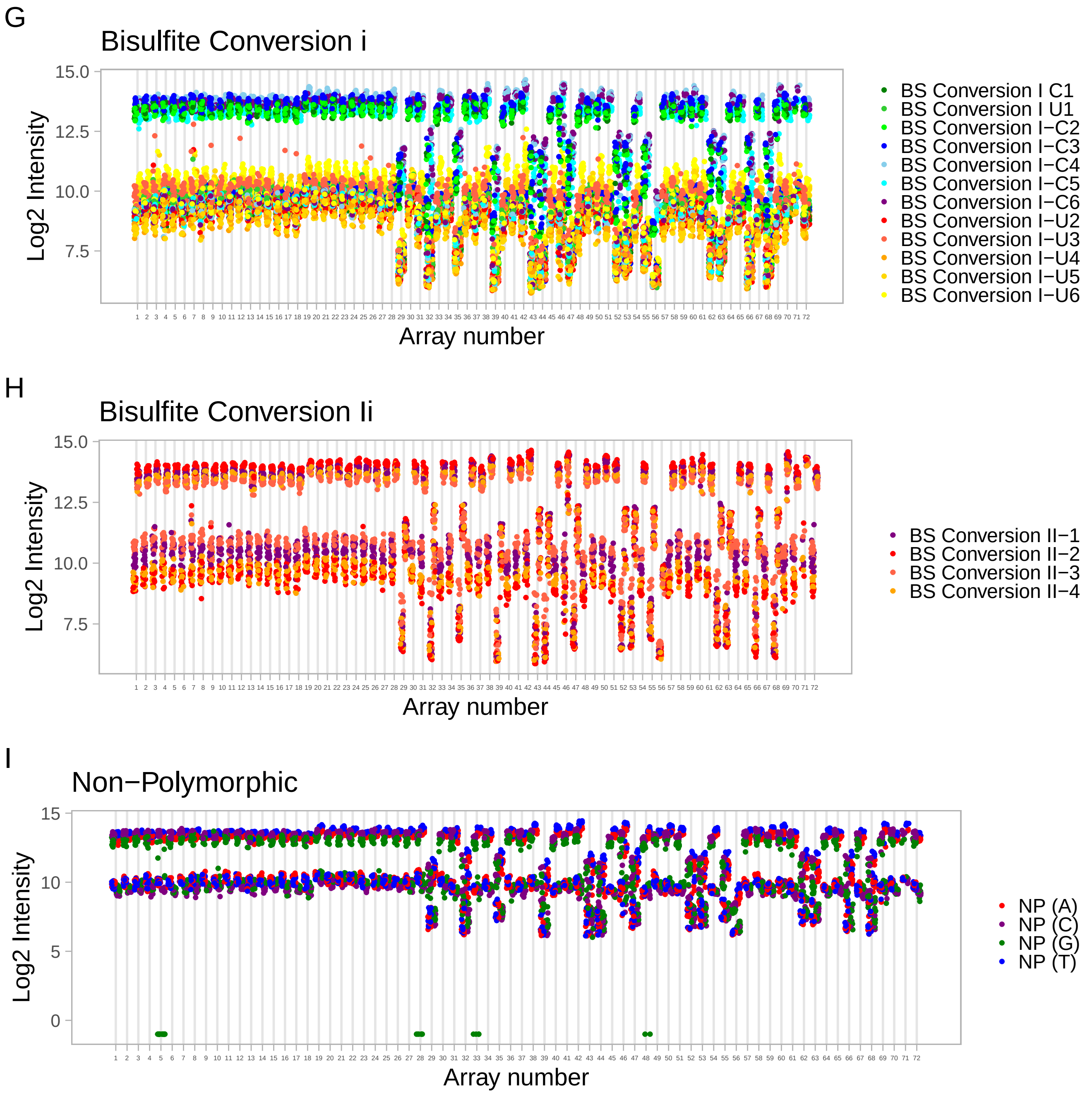
G
Bisulfite Conversion i
Log2 Intensity
15.0
12.5
10.0
7.5
Array number
BS Conversion I C1
BS Conversion I U1
BS Conversion I−C2
BS Conversion I−C3
BS Conversion I−C4
BS Conversion I−C5
BS Conversion I−C6
BS Conversion I−U2
BS Conversion I−U3
BS Conversion I−U4
BS Conversion I−U5
BS Conversion I−U6
H
Bisulfite Conversion Ii
Log2 Intensity
15.0
12.5
10.0
7.5
Array number
BS Conversion II−1
BS Conversion II−2
BS Conversion II−3
BS Conversion II−4
I
Non−Polymorphic
Log2 Intensity
15
10
5
0
Array number
NP (A)
NP (C)
NP (G)
NP (T)

J

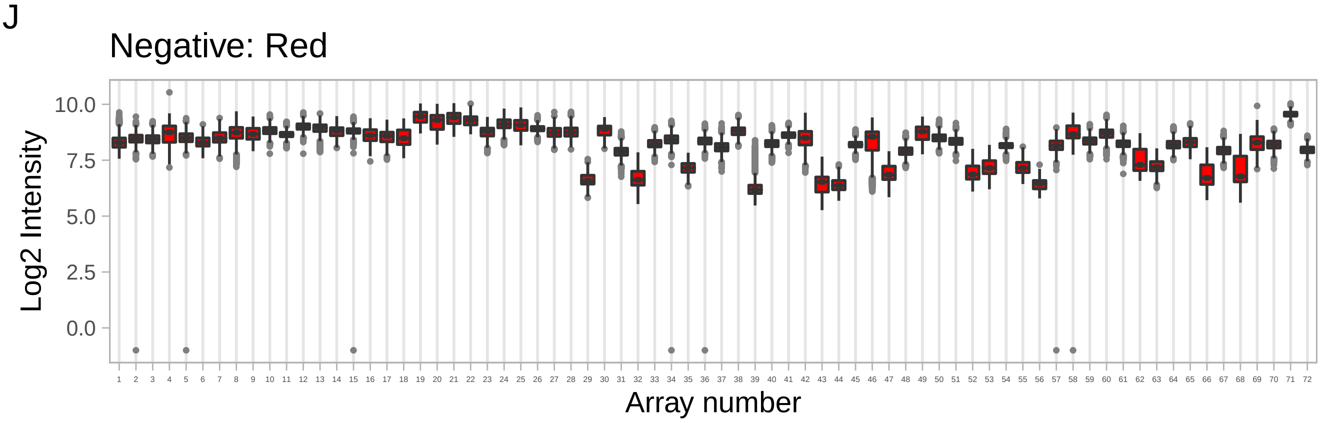
Negative: Red
Log2 Intensity
10.0
7.5
5.0
2.5
0.0
Array number

K

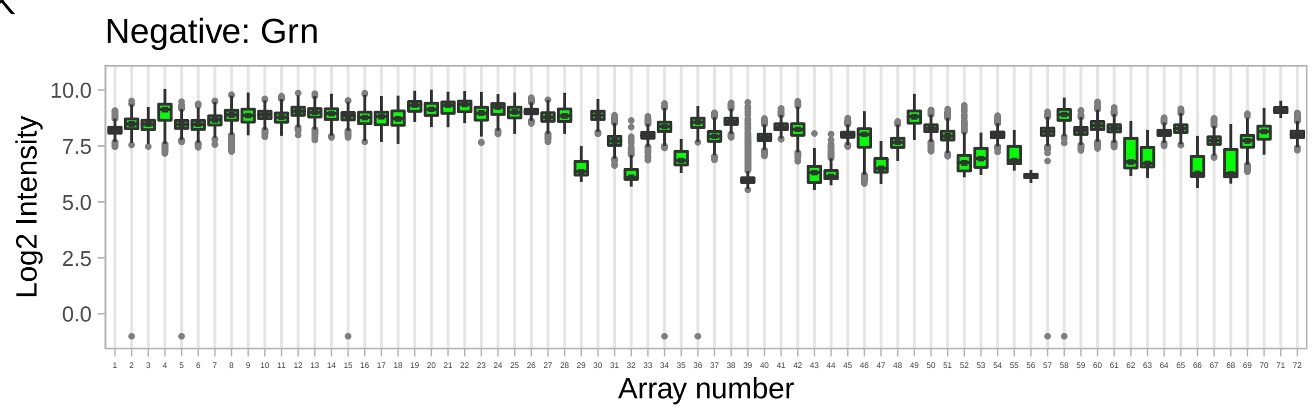
Negative: Grn
Log2 Intensity
10.0
7.5
5.0
2.5
0.0
Array number
