# Supplementary material for: Batch-effect detection, correction and characterisation in Illumina HumanMethylation450 and MethylationEPIC BeadChip array data
Source: Clin Epigenetics. 2022 Apr 29;14:58. doi: 10.1186/s13148-022-01277-9 (PMC9055778; doi:10.1186/s13148-022-01277-9)
Supplement: Supplementary file 9 — Additional file 9: Figure S9. Full set of control probes for the NOVI study. Further detail on the controls is provided by Illumina in the BeadArray Controls Reporter Software Guide document. [file 13148_2022_1277_MOESM9_ESM.pdf]

NOVI

A

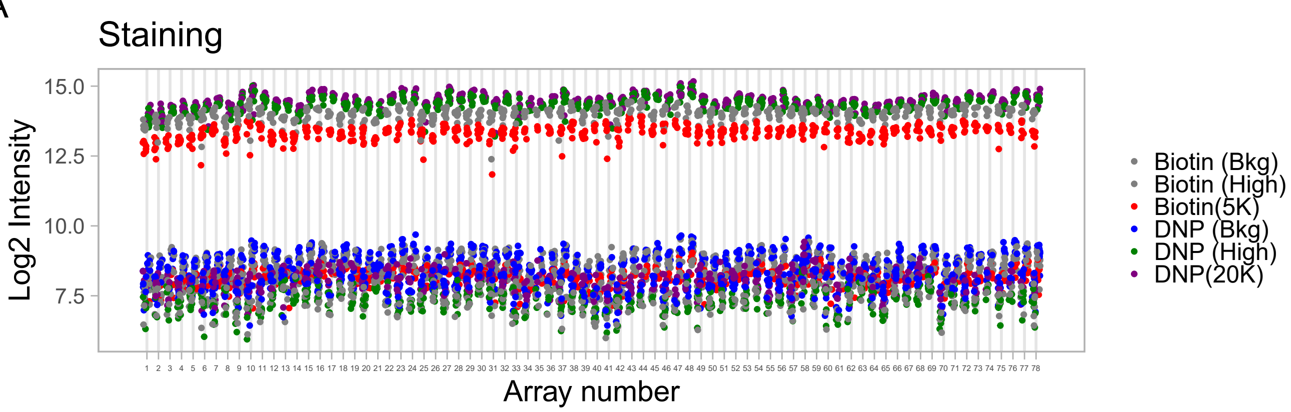

Staining
Log2 Intensity
15.0
12.5
10.0
7.5
Array number
Biotin (Bkg)
Biotin (High)
Biotin(5K)
DNP (Bkg)
DNP (High)
DNP(20K)

B

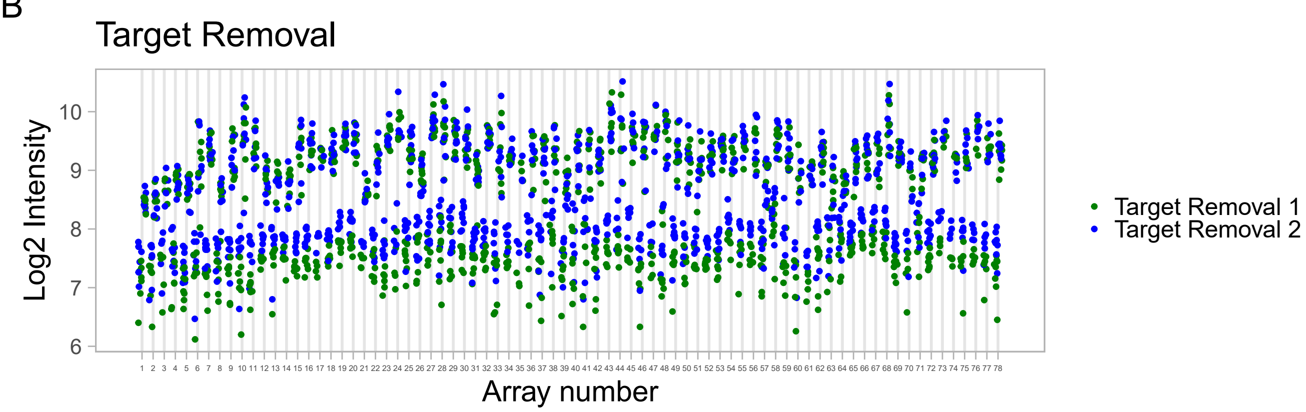

Target Removal
Log2 Intensity
10
9
8
7
6
Array number
Target Removal 1
Target Removal 2

C

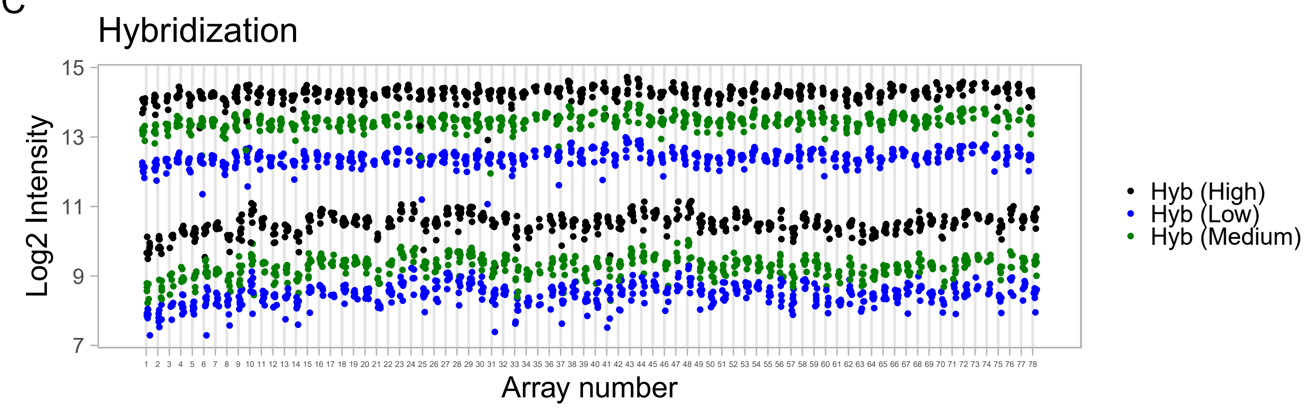

Hybridization
Log2 Intensity
15
13
11
9
7
Array number
Hyb (High)
Hyb (Low)
Hyb (Medium)

D

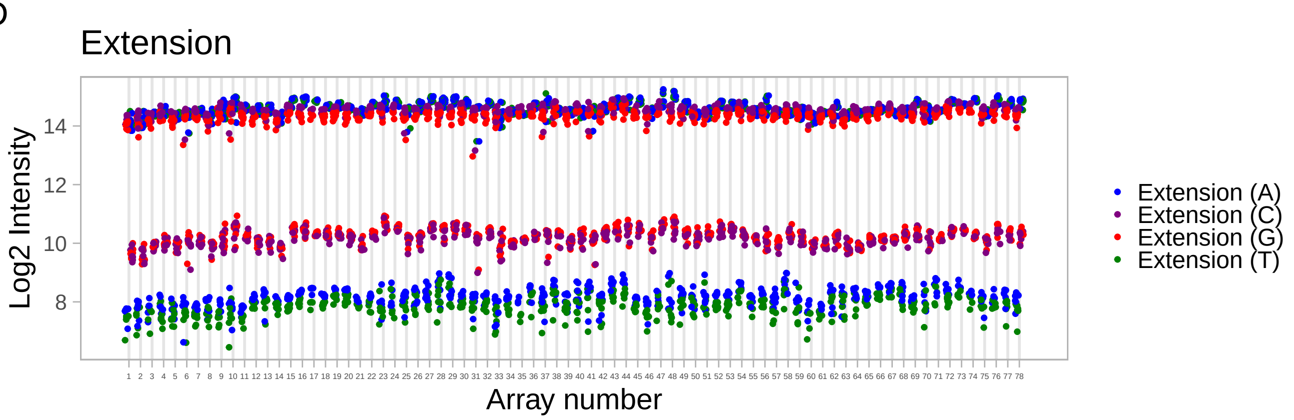

Extension
Log2 Intensity
14
12
10
8
Array number
Extension (A)
Extension (C)
Extension (G)
Extension (T)

E

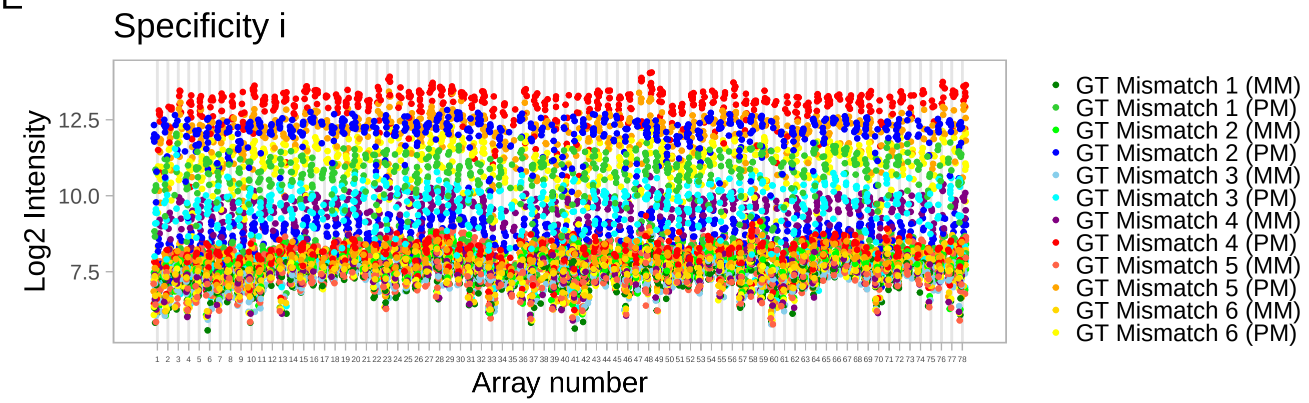

Specificity i
Log2 Intensity
12.5
10.0
7.5
Array number
GT Mismatch 1 (MM)
GT Mismatch 1 (PM)
GT Mismatch 2 (MM)
GT Mismatch 2 (PM)
GT Mismatch 3 (MM)
GT Mismatch 3 (PM)
GT Mismatch 4 (MM)
GT Mismatch 4 (PM)
GT Mismatch 5 (MM)
GT Mismatch 5 (PM)
GT Mismatch 6 (MM)
GT Mismatch 6 (PM)

F

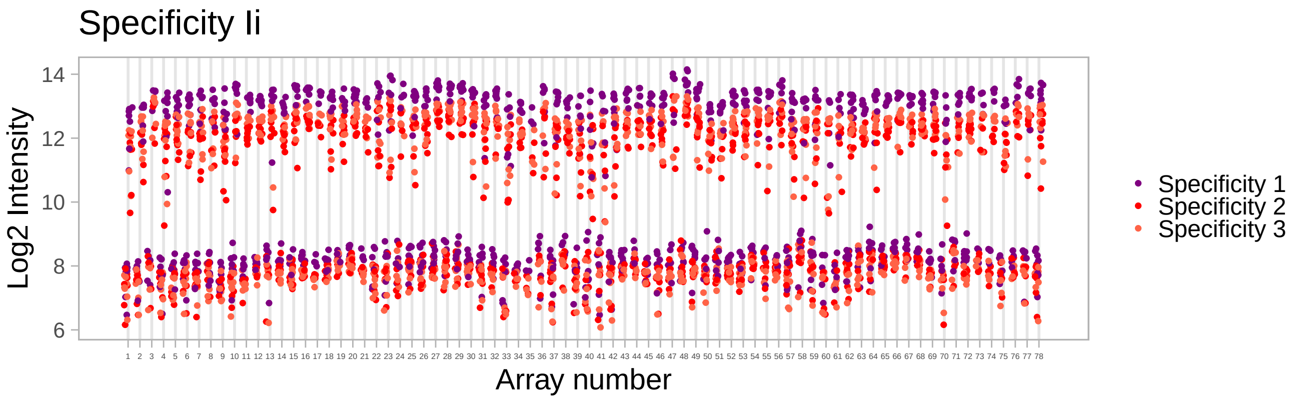

Specificity Ii
Log2 Intensity
14
12
10
8
6
Array number
Specificity 1
Specificity 2
Specificity 3

G

Bisulfite Conversion i

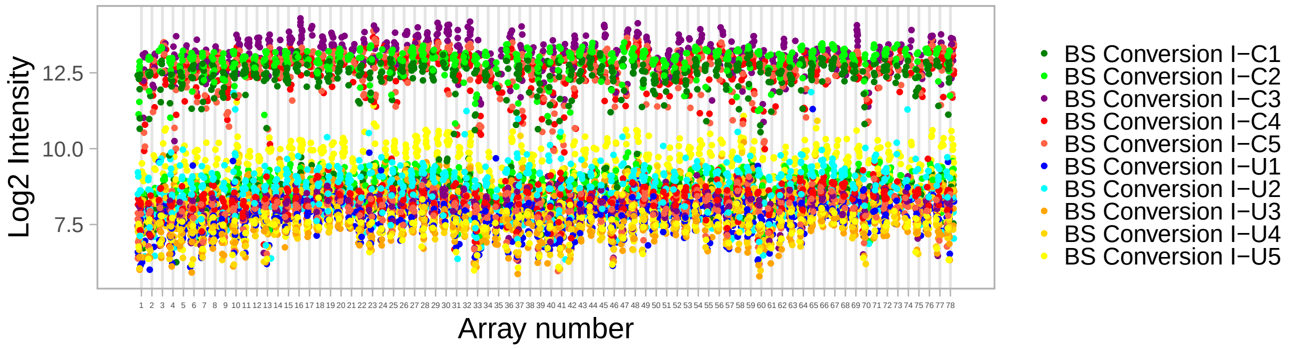

H

Bisulfite Conversion Ii

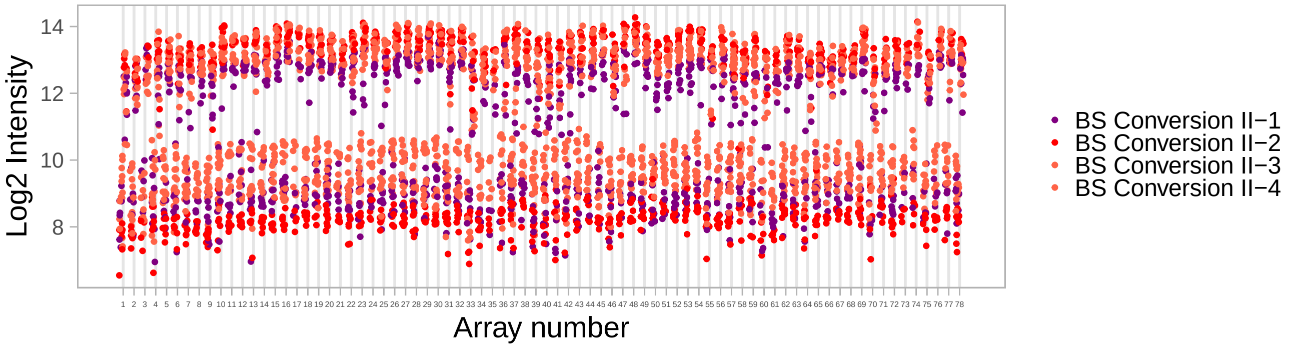

I

Non−Polymorphic

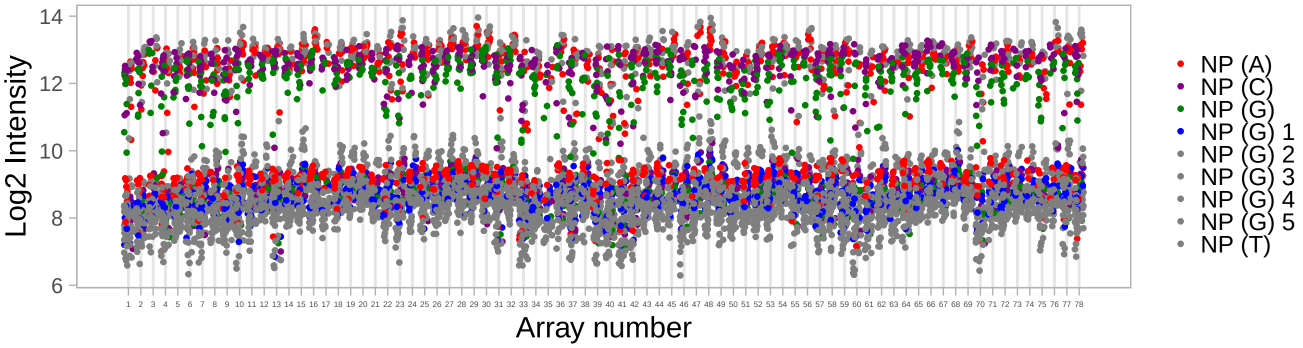

J

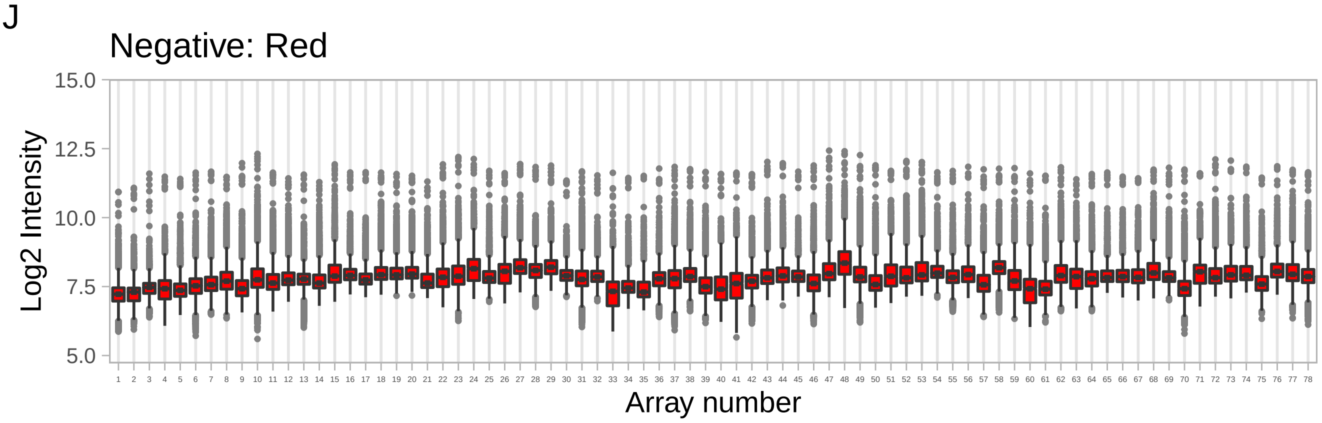
Negative: Red
Log2 Intensity
Array number

K

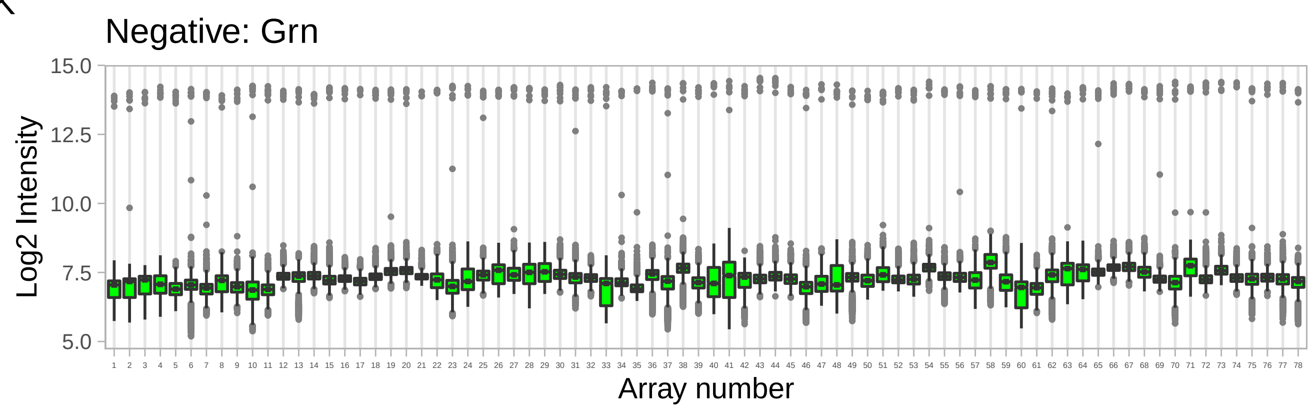
Negative: Grn
Log2 Intensity
Array number
